# Unfolding and Degradation of Micellar Immunodrug Carriers Derived From End Group Modified Aliphatic Poly(Carbonate)s with Acid‐Responsive Ketal Side Groups

**DOI:** 10.1002/adma.202511752

**Published:** 2025-12-12

**Authors:** Adrian V. Hauck, Michael Fichter, Laura J. Rosenberger, Jannis Willig, Alexander Fuchs, Felicia Schön, Paul Schneider, Carolina Medina‐Montano, Taufiq Ahmad, Jessica Erlenbusch, Pol Besenius, Leonard Kaps, Matthias Bros, Stephan Grabbe, Volker Mailänder, Jürgen Groll, Lutz Nuhn

**Affiliations:** ^1^ Institute of Functional Materials and Biofabrication, Center of Polymers for Life, Department of Chemistry and Pharmacy Julius‐Maximilians‐Universität Würzburg 97074 Würzburg Germany; ^2^ Department of Dermatology University Medical Center (UMC) of the Johannes Gutenberg‐University Mainz 55131 Mainz Germany; ^3^ Department of Chemistry Johannes Gutenberg‐University Mainz 55122 Mainz Germany; ^4^ Department for Medicine II University Medical Center Saarland 66421 Homburg/Saar Germany; ^5^ Max Planck Institute for Polymer Research 55128 Mainz Germany

**Keywords:** block copolymer micelle, FRET, immunodrug delivery, ketal, polycarbonates

## Abstract

Small‐molecule immunodrugs hold significant promise for immunotherapeutic applications including vaccination and cancer therapy. However, their clinical use is limited by severe side effects resulting from systemic distribution. To address this challenge, a biodegradable, acid‐responsive nanocarrier system designed for controlled immunodrug delivery in vivo is presented. It is based on polymeric micelles that disassemble in response to acidic environments, enabling site‐specific particle unfolding following endocytosis by antigen‐presenting cells. Its core structure features a hydrolysable aliphatic poly(carbonate) backbone, promoting both biocompatibility and biodegradability. The high precision of the applied ring‐opening polymerization allows for polymer end group functionalization, including the integration of fluorophores for Förster resonance energy transfer (FRET)‐based monitoring of particle integrity and unfolding. The observations reveal a pH‐dependent disassembly profile both in vitro and in vivo. Comparative studies with non‐responsive poly(carbonate)s demonstrate the superior performance of the acid‐responsive design in selectively disassembling under acidic conditions and enhancing polymer backbone degradation. Furthermore, covalent conjugation of a small molecule Toll‐like receptor 7/8 agonist promotes its controlled delivery in vitro and in vivo, resulting in improved immune cell uptake and regulated cytokine production. The findings underscore the potential of this biodegradable, acid‐responsive micellar nanocarrier system as precision delivery platform for safer and effective immunotherapeutics.

## Graphical abstract

An acid‐responsive polymeric micelle system based of aliphatic poly(carbonate)s with benzyl ketal side chains is designed for targeted immunodrug delivery. It can disassemble in endosomal environments, enabling site‐specific immunoactivation and enhanced biodegradability. Covalent end group conjugation of a TLR7/8 agonist regulates immune cell activation and cytokine regulation, offering a promising platform for safer, more effective immunotherapeutics.

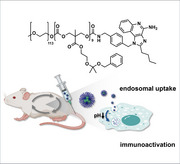

## 1 Introduction

Over the last decades immunotherapy has gained much attention as promising fourth pillar in oncology. Alongside established cancer treatments such as chemotherapy, radiotherapy, and surgery, immunotherapy offers the potential to complement these approaches as highly effective but less invasive and less toxic treatment modality.^[^ ^1^, ^2^ ^]^

Immunodrugs aim at activating the body's innate immune system via stimulation of pattern recognition receptors (PRRs) and serving either as direct tumor treatment or as adjuvant in cancer vaccines.^[^ ^3^, ^4^ ^]^ Promising candidates are Toll‐like receptor (TLR) agonists.^[^ ^5^, ^6^, ^7^ ^]^ TLRs located on cell membranes can be stimulated by lipopolysaccharides (TLR4), whereas TLRs apparent in endosomes and lysosomes are triggered by nucleic acids like poly(I:C)‐ (TLR3) or CpG‐oligonucleotides (TLR9), or synthetic small‐molecule imidazoquinolines (TLR7/8), to name a few.^[^ ^8^, ^9^ ^]^ A significant milestone in this field is the use of the TLR7/8 agonist IMDG as an adjuvant in Bharat Biotech's COVID‐19 vaccine, Covaxin (BBV152).^[^ ^10^, ^11^, ^12^ ^]^ However, the use of many immunotherapeutics remains limited due to severe side effects, including headache, fever and cytokine storm associated with uncontrolled body distribution of the applied adjuvant.^[^ ^13^, ^14^ ^]^ Besides, some small‐molecule immunodrugs also have a poor bioavailability due to their hydrophobic nature.^[^ ^15^ ^]^ The use of polymeric nanocarriers has the potential to overcome these limitations. In addition to simple drug solubilization, nanocarriers can amplify on‐target immune activation and suppress adverse events.^[^ ^16^, ^17^ ^]^

Among other polymer‐based nanocarriers, polymeric micelles represent a particularly promising platform. They are readily accessible in terms of formulation and sterilization and can be produced on a nanometer size‐scale, perfectly suitable for biomedical applications.^[^ ^18^, ^19^ ^]^ The amphiphilic nature of polymeric micelles allows for the encapsulation of hydrophobic cargos within their cores, improving the immunodrugs’ bioavailability.^[^ ^20^ ^]^ Additionally, polymeric micelles increase circulation times by reducing renal clearance, and they can facilitate the transport of active cargos into the tumor microenvironment (TME) and its draining lymph nodes, minimizing off‐target immune responses.^[^ ^21^, ^22^ ^]^ Since polymeric micelles are composed of individual polymer chains, they can have the tendency to form undesired aggregates with other amphiphilic biomacromolecules. Thus, a stimulus‐responsive hydrophilization is desired. Such unique properties allow these micelles to provide an ideal balance between stability and degradability.

To promote on‐target drug release, nanoparticles can be designed to respond to environmental changes (pH, redox potential and enzymes) in target tissues or to external stimuli (temperature, light, radiation, ultrasound).^[^ ^23^ ^]^ The decrease of the pH value compared to the physiological environment (pH 7.4) is of particular interest since it occurs during endosomal (pH 5.5–6.0) and lysosomal (pH 4.5–5.0) cell uptake as well as in the TME (pH 6.0–7.0).^[^ ^24^, ^25^ ^]^ Compared to other acid‐responsive motives (imines, hydrazones, orthoesters, esteracetals) ketal functionalities are especially compelling because of their high stability in physiological environments but rapid hydrolysis at contemplated pH levels (pH 4.5–6.0).^[^ ^26^, ^27^, ^28^, ^29^ ^]^ Moreover, while polymeric versions of hydrazones or imines liberate toxic by‐products of polyamines, polyhydrazines or polyaldehydes, the ketal functionality falls apart into polyols that are of less concern for biomedical applications.

In previous works, we reported on the development of micellar nanocarriers formulated from amphiphilic block copolymers, based on polyethylene glycol (PEG) and aliphatic poly(carbonates).^[^ ^15^, ^30^, ^31^, ^32^, ^33^ ^]^ While PEG serves as hydrophilic shell providing a protecting stealth effect,^[^ ^34^ ^]^ the aliphatic poly(carbonate) block with benzyl side groups builds the hydrophobic core of the polymeric micelles. Its poly(carbonate) backbone assures gradual hydrolytic biodegradability,^[^ ^35^ ^]^ while acid‐sensitive on‐target carrier decomposition can be promoted by incorporating pH‐responsive ketal functionalities into the poly(carbonate) block side chains.^[^ ^36^ ^]^ They lead to micelle disassembly under acidic conditions. A transient stability can be achieved for such micelles with benzyl ketals that remain stable in the blood stream but undergo rapid micelle unfolding in acidic environments. However, physical encapsulation of hydrophobic drugs as guest molecules into the hydrophobic cores cannot guarantee their safe delivery.^[^ ^37^, ^38^, ^39^, ^40^ ^]^ Too often, such micelles suffer from premature drug leakage during systemic application, which is particularly fatal for highly active immunostimulators.^[^ ^13^, ^14^ ^]^ Thus, we aim at implementing in this study an alternative approach by precision delivery through covalent conjugation of the small molecule immunodrug to the pH‐responsive micellar core.

In the present work, we therefore make use of the precise control offered by the ring‐opening polymerization of cyclic carbonates to obtain well‐defined block copolymers with modifiable end groups. This allows us to expand our system of benzyl ketal functionalized aliphatic poly(carbonates)^[^ ^36^ ^]^ by performing precise polymer end group functionalizations for the covalent conjugations of fluorescence dyes and the highly promising immunodrug IMDQ. In a head‐to‐head study these micellar nanocarriers could be compared to a non‐responsive benzyl ester functionalized polycarbonate system.^[^ ^15^ ^]^ The end group introduction of fluorescent probes allows for real‐time monitoring of the particles both in vitro and in vivo, providing insight into their body distribution. Moreover, co‐formulation of Förster resonance energy transfer (FRET) dye‐labeled block copolymers facilitates the quantitative evaluation of their FRET ratio as an indicator of micelle integrity, offering deeper insight into the micelles’ unfolding behavior in vitro and in vivo. Additionally, the covalent conjugation of the TLR7/8 agonist IMDQ at the block copolymers’ end groups facilitated the drug's administration, enhancing its formulation safety profile and efficacy. A superior performance of the ketal‐functionalized aliphatic polycarbonates compared to the non‐responsive aliphatic polycarbonate control system emphasizes the beneficial advantages of the presented acid‐responsive micellar nanocarrier, highlighting its high potential for precision immunodrug delivery applications.

## 2 Results and Discussion

**Figure 1:**
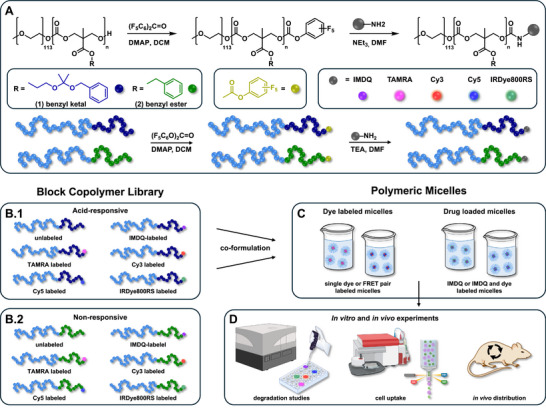
Overview of the block copolymer library with different end group labels for versatile polymeric micelle formulations. A) The end groups of the two block copolymers, the benzyl ketal functionalized poly(ethylene glycol)_113_‐*b*‐poly(MTC‐OEtKBn)_n_ (1) and the benzyl ester functionalized poly(ethylene glycol)_113_‐*b*‐poly(MTC‐OBn)_m_ (2), were functionalized via a two‐step synthesis, first introducing an active PFP‐carbonate end group, followed by the substitution of pentafluorophenol with monoamine functionalized drugs or dyes. B) Following this strategy, a block copolymer library with different end group labels was synthesized from the acid‐responsive block copolymers (B.1) and the non‐responsive control system (B.2). C) The co‐formulation of differently end group‐labeled block copolymers results in polymeric micelles with versatile combinations of dyes and/or drugs inside the polymeric micelles core. D) This approach allows for performing multiple in vitro and in vivo experiments to study their immunostimulatory as well as unfolding properties (created with BioRender).

To gain a comprehensive insight into the performance of polymeric micelles derived from acid‐responsive poly(ethylene glycol)_113_‐*b*‐poly(MTC‐OEtKBn)_19_ (**1**) and to highlight their advantage over the non‐responsive counterpart poly(ethylene glycol)_113_‐*b*‐poly(MTC‐OBn)_21_ (**2**), a block copolymer library was synthesized featuring various end group modifications (**Figure** 1). This was achieved via activated pentafluorophenyl (PFP) carbonate chemistry, enabling the covalent attachment of monoamine‐functionalized molecules to the block copolymers’ chain ends (Figure 1A) (in principle, pentafluorophenyl activation can also be applied to transesterification by alcohols or aminolysis with secondary amines, however, elevated reaction temperature conditions and prolonged reaction times would then be needed that could affect the integrity of the polycarbonate). Following this strategy, the TLR 7/8 agonist IMDQ and a series of fluorescence dyes bearing a single primary amine (TAMRA, Cy3, Cy5, IRDye800RS) were covalently conjugated to the block copolymers’ chain ends (Figure 1B). By varying the combination of labeled block copolymers, a diverse set of polymeric micelle formulations was generated (Figure 1C). In addition to the single dye‐ and IMDQ‐labeled particles, co‐formulation of Cy3‐ and Cy5‐labeled block copolymers provided a FRET‐based monitoring of the micelles’ integrity. Furthermore, co‐formulating dye‐ and IMDQ‐labeled block copolymers allowed for multifaceted experimental designs (Figure 1C), facilitating simultaneous analysis of IMDQ‐induced immune stimulation, nanoparticle biodistribution and unfolding as well as cell uptake both in vitro and in vivo (Figure 1D).

### 2.1 Block Copolymer Synthesis

The pH responsive block copolymer poly(ethylene glycol)_113_‐*b*‐poly(MTC‐OEtKBn)_n_ (**1**) with acid degradable ketal side groups and the non‐responsive control system poly(ethylene glycol)_113_‐*b*‐poly(MTC‐OBn)_m_ (**2**) with benzyl ester side groups were both synthesized by base‐catalyzed ring opening polymerization (ROP). To achieve controlled polymerization progress, it is crucial to work under dry conditions. Therefore, the macroinitiator mPEG_113_‐OH and the respective monomer (MTC‐OEtKBn or MTC‐OBn) were dried by azeotropic distillation with benzene three times. The polymerizations were performed in dichloromethane (DCM) and 1,8‐diazabicyclo(5.4.0)undec‐7‐ene (DBU) was used as catalyst. Both components were dried over calcium hydride and distilled freshly before use. To further ensure high control over the polymerizations, they were conducted at −20 °C. Thereby, it was possible to synthesize two polymers, with similar numbers of repeating units, that were suited for direct head‐to‐head comparison. According to the ^1^H NMR spectrum (Figure S1, Supporting Information), the carbonate block of poly(ethylene glycol)_113_‐*b*‐poly(MTC‐OEtKBn)_n_ (**1**) provided *n* =  19 repeating units. For poly(ethylene glycol)_113_‐*b*‐poly(MTC‐OBn)_m_ (**2**) *m* =  21 repeating units were found (Figure S4, Supporting Information). Gel permeation chromatography (GPC) and matrix assisted laser desorption ionization time of flight (MALDI‐ToF) mass spectrometry (MS) confirmed quantitative block copolymer chain growth from the macroinitiator and conserved narrow dispersities (*Đ* = 1.04) for both block copolymers (Figures S2,S3 and S5,S6, Supporting Information, note that the smaller molecular weight species (note that the smaller molecular weight species found by MALDI‐ToF MS at half of the original molecular weight can be assigned to the double charged species, identified by an isotope pattern differing by 0.5 Da instead of 1.0 Da – compare Figures S3,S6 (Supporting Information), as well as all further collected data for the various end group modified block copolymers, too).

To assess the storage stability of the synthesized poly(ethylene glycol)_113_‐*b*‐poly(MTC‐OEtKBn)_19_ block copolymers (**1**), we further investigated their thermal properties. Thermogravimetric analysis (TGA) results, revealed that the benzyl ketal‐functionalized carbonate block copolymer, poly(ethylene glycol)_113_‐*b*‐poly(MTC‐OEtKBn)_19_ remains thermally stable up to ≈200 °C (Figure S7, Supporting Information). Beyond this temperature, a decomposition of the benzyl ketal functionalities begins, followed by degradation of the carbonate block between 260 °C and 300 °C, and finally thermal decomposition of the PEG block in the range of 380–420 °C (Figure S7, Supporting Information). These findings highlight the suitable thermal stability of the material under inter conditions. Further thermal analysis was conducted using differential scanning calorimetry (DSC) (Figure S8, Supporting Information). The macroinitiator poly(ethylene glycol)_113_ exhibited typical semicrystalline behavior, with a melting observed between 55 °C and 70 °C during heating and crystallization between 35 °C and 45 °C during cooling. In contrast, the homopolymer poly(MTC‐OEtKBn) displayed a glass transition around –5 °C to +5 °C (heating) and –10 °C to 0 °C (cooling), along with a weak melting transition near 140–150 °C and crystallization between 105 °C and 125 °C. The block copolymer poly(ethylene glycol)_113_‐*b*‐poly(MTC‐OEtKBn)_19_ exhibited both the glass transition of the poly(MTC‐OEtKBn) block and the melting/crystallization transitions of the PEG block, albeit at slightly reduced temperatures. Specifically, the glass transition occurred around –15 °C to 0 °C during heating and –10 °C to 0 °C during cooling, while the melting and crystallization transitions of the PEG block were observed at 40 °C–60 °C and 10 °C–30 °C, respectively. Collectively, these thermal analyses suggest that the block copolymer can be stored in a semi‐crystalline state, which contributes to its overall thermal robustness and long‐term stability.

### 2.2 End Group Functionalization

**Figure 2:**
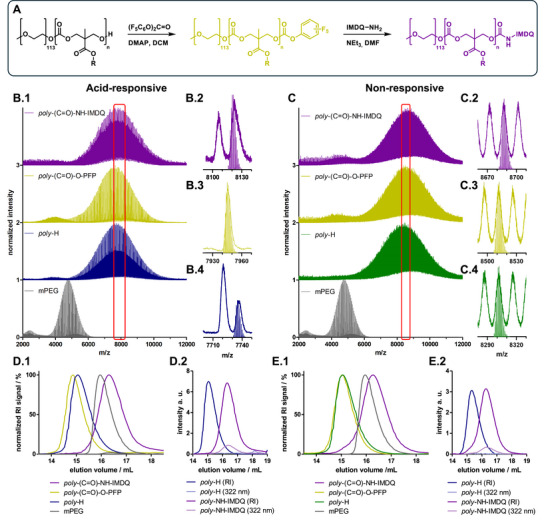
Exemplary end group functionalization of mPEG_113_‐*b*‐p(MTC‐OEtKBn)_n_ (1) and mPEG_113_‐*b*‐p(MTC‐OBn)_m_ (2) providing IMDQ conjugation to the acid‐responsive block copolymer (left) and the non‐responsive control system (right). A) Synthesis scheme for the two‐step end group functionalization. First an activated PFP‐carbonate end group is generated, followed by aminolysis of the PFP‐carbonate with IMDQ‐NH_2_. B.1) MALDI‐ToF mass spectra of acid‐responsive block copolymers (1) at different functionalization stages next to the spectrum of the macroinitiator mPEG_113_‐OH. The red box indicates the regions magnified in B.2–B.4. These excerpts show the mPEG_113_‐*b*‐p(MTC‐OEtKBn)_8_ (1) simulation cationized with K^+^, carrying the respective end group next to the corresponding MALDI‐ToF measurement. Simulations for mPEG_113_‐*b*‐p(MTC‐OBn)_13_ (2) cationized with K^+^, carrying respective end groups and the corresponding measurements are shown in C.1–C.4. The normalized GPC elugrams (RI‐detection) of polymers at different functionalization stages are shown in D.1 for the acid‐responsive system and in E.1 for the non‐responsive control system. Furthermore, RI‐traces are compared to UV‐traces at the IMDQ specific detection wavelength at 322 nm for unmodified and IMDQ‐conjugated polymers in D.2 for the acid‐responsive system and in E.2 for the non‐responsive control system.

The high precision of the base‐catalyzed ROP provided block copolymers with accessible hydroxyl end groups for both the acid‐responsive block copolymer (**1**) and the non‐responsive control system (**2**). They could be further modified in a two‐step synthesis process, which is exemplarily shown for the covalent attachment of TLR 7/8 agonist IMDQ in **Figure** **2**. First, an activated PFP‐carbonate end group was introduced to the block copolymers hydroxyl chain end by treatment with bis(pentafluorophenyl)‐carbonate (Figure 2A). The reaction was conducted in dry DCM and 4‐dimethylaminopyridine (DMAP) was used as catalyst. After isolating both polymers, they were characterized by ^1^H and ^19^F NMR spectroscopy as well as GPC (Figures S9–S14, Supporting Information). The collected polymers were then used in a second step for aminolysis of the activated carbonate end group substituting the pentafluorophenyl group with the monoamine functionalized IMDQ. This reaction was performed in dry *N*,*N*‐dimethylformamide (DMF) and catalyzed with triethylamine (TEA). The isolated end group functionalized block copolymers were characterized by GPC and MALDI‐ToF mass spectrometry as well as ^1^H and ^19^F NMR spectroscopy (Figures S20–S24, Supporting Information).

A comparison of the MALDI‐ToF mass spectra in Figure 2B (acid‐responsive block copolymer **1**) and Figure 2C (non‐responsive control system **2**) is shown for block copolymers with shorter carbonate block lengths, and clear shifts of each polymer species after each functionalization step were observed. Compared to the macroinitiator mPEG_113_‐OH, the block copolymer mass distributions were overall distinctively shifted toward higher molecular weights. Examining the individual peaks of the spectra allows for a precise tracking of the mass shifts after each functionalization step. Figure 2B.2–2B.4, 2C.2–2C.4 shows the isotope simulations for mPEG_110_‐*b*‐p(MTC‐OEtKBn)_8_ (**1**) and mPEG_113_‐*b*‐p(MTC‐OBn)_13_ (**2**) (cationized with K^+^) carrying the respective end group next to the corresponding MALDI‐ToF measurement. The direct overlay of measurements and simulations provide strong evidence for a successful quantitative end group modification.

In addition, successful quantitative end group functionalization could also be evaluated by GPC (Figure 2D.1–2D.2 and 2E.1–2E.2). Compared to the mPEG_113_‐OH macroinitiator, the GPC elugrams of the acid‐responsive block copolymers (Figure 2D.1) and the non‐responsive control polymers (Figure 2E.1) show strong shifts toward smaller elution volumes, corresponding to higher molecular weights. The same observation could be made for the PFP‐carbonate end group functionalized polymers. In contrast, the IMDQ‐labeled polymers shifted again toward lower molecular weights. This observation can be associated to the poor solubility behavior of IMDQ itself in THF, yielding a collapse of the IMDQ‐carrying polymers in the GPC solvent. While the hydroxyl end group and the PFP carbonate end group are well swollen in THF suggesting a swollen random polymer coil, the incorporation of the IMDQ group reduces the hydrodynamic volume resulting in an increased retention time. Alternatively, GPC measurements of the IMDQ end group functionalized block copolymers in hexafluoroisopropanol as eluent and good solvent for IMDQ restored again the typical increase in molecular weight for both block copolymer species compared to the mPEG_113_‐OH macroinitiator (Figures S16,S21, Supporting Information).

Moreover, for an unambiguous proof that these polymers were carrying IMDQ, the UV‐detector of the GPC was set to IMDQ's absorbance at 322 nm (Figure 2D.2, 2E.2). While the initial block copolymers without IMDQ were only found by RI detector, and no UV absorbance could be recorded for them at 322 nm, the IMDQ‐labeled block copolymers could now be successfully characterized by both RI detector and UV detector at 322 nm. Moreover, ^1^H and ^19^F NMR spectroscopy further verified the removal of the pentafluorophenyl carbonate and successful conjugation of the IMDQ group to the end groups (Figures S18,S19 and Figures S23,S24, Supporting Information quantified for shorter block copolymer versions). In combination with the MALDI‐ToF mass spectrometry data, these measurements prove the successful end group IMDQ conjugation of the two block copolymers.

In analogy, the dye‐labeling with monoamine functionalized fluorophores (TAMRA, Cy3, Cy5, IRDye800RS) was performed and the labeled polymers were analyzed by GPC and MALDI‐ToF mass spectrometry, as discussed for the IMDQ conjugates before (Figures S25–S40, Supporting Information – TAMRA end group modification could also be further verified by ^1^H and ^19^F NMR spectroscopy, compare Figures S27,S28, Supporting Information). Moreover, in all MALDI‐ToF spectra an agreement between recorded data and simulated isotope distributions could be found for each polymer, while by GPC analysis a similar polymer collapse could only be detected after IRDye800RS conjugation (following the poor solubility of this dye in the GPC solvents), while all other fluorophores themselves are well soluble and, thus, provided the typical decrease in elution volume or increase in molecular weight as expected (Figure S25–S40, Supporting Information). The synthesized polymer library provides a substantial platform for subsequent head‐to‐head comparison of the two block copolymers and their derived polymeric micelles, allowing for comprehensive structure‐property analyses of their drug delivery properties and degradation profiles.

### 2.3 Backbone Degradation Study and Critical Micelle Concentration (CMC)

**Figure 3:**
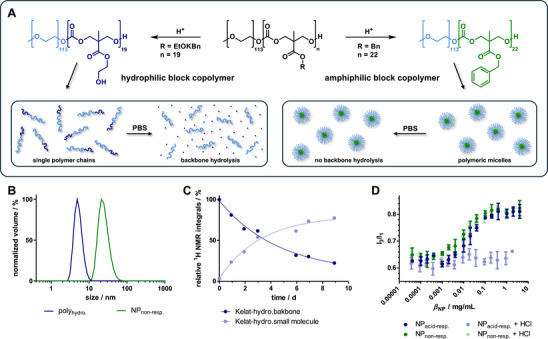
A) Schematic degradation process of acid‐responsive poly(ethylene glycol)_113_‐*b*‐poly(MTC‐OEtKBn)_19_ (1) and non‐responsive poly(ethylene glycol)_113_‐*b*‐poly(MTC‐OBn)_21_ (2). In case of the acid‐responsive system, acidification leads to ketal hydrolysis while the non‐responsive control system remains intact. In a second degradation step, only the hydrophilized block polymer chains undergo backbone hydrolysis under physiological conditions (PBS at pH 7.4 and 37 °C). The amphiphilic block copolymers, on the other hand, form stable micelles that prevent polycarbonate backbone hydrolysis. B) DLS measurement of formulated polymers. The amphiphilic block copolymers form nanoparticles (NP_non‐resp._) while for the hydrophilized block copolymer (poly_hydro._) single polymer chains were found. C) Results of the hydrolytic backbone degradation study provided by the relative ^1^H NMR integrals of polymer backbone signals and small molecule fragments recorded over time. D) CMC determination showing pyrene's I_3_/I_1_ fluorescence intensities plotted against block copolymer concentration *β*_NP_ for both samples before and after acidification. While for the non‐responsive block copolymer no difference was observed before and after treatment with acid, the acid‐responsive block copolymer loses its self‐assembly behavior and provides no CMC anymore after treatment with acid.

Besides controlled side group degradation upon ketal side chain hydrolysis (compare Figure S41, Supporting Information), the acid‐responsiveness of the new nanocarrier system also promotes gradual hydrolytic backbone degradation of the aliphatic poly(carbonate) backbone. As a result of acidic treatment, the ketal side groups turn into more hydrophilic hydroxyl groups that facilitate backbone hydrolysis. To get a deeper insight into this process, we investigated the backbone hydrolysis of both nanocarriers after acidification. For this purpose, the polymers were first treated with trifluoroacetic acid (TFA) triggering ketal hydrolysis in case of the acid‐responsive system (**1**) (**Figure** **3**A left, confirmed by ^1^H NMR spectroscopy and GPC analysis in Figures S42,S43, Supporting Information), while the non‐responsive control system (**2**) did not react under these conditions (Figure 3A right, confirmed by ^1^H NMR spectroscopy and GPC analysis in Figures S44,S45, Supporting Information). After isolating both polymers, they were formulated into micelles by a solvent evaporation method in deuterated PBS. The self‐assembly into polymeric micelles was confirmed by dynamic light scattering (DLS) (compare Figure S53, Supporting Information). As shown in Figure 3B, only the intact amphiphilic block copolymers of the non‐responsive control system formed nanoparticles (NP_non‐resp._) with sizes between 10 and 100 nm. For the side group degraded hydrophilic block copolymers (poly_hydro._) smaller sizes were found below 10 nm that can be assigned to single polymer chains. This observation is in line with the acidic micelle degradation of this nanocarrier, we reported earlier.^[^ ^36^ ^]^ Subsequently, the hydrolytic stability of the poly(carbonate) backbone was investigated for these polymers under physiological conditions (pH 7.4, ϑ **=** 37 °C) by ^1^H NMR spectroscopy. While for the micelle‐forming block copolymers only the PEG shell of polymeric micelles is detectable by NMR spectroscopy (Figure S46B.1, Supporting Information), the hydrophilized block copolymers provide fully solubilized polymers that can be analyzed completely (Figure S46A.1, Supporting Information). The polymers also remain fully water‐soluble at elevated temperature, thus, confirming the absence of any cloud‐point properties for these polymers (compare Supporting Information Figure S50, Supporting Information). Over time the detected poly(carbonate) backbone signals decreased while increasing signals of the hydrolyzed small molecule diols were found (note that the ethylene glycol ester side group is not affected). The formation of these small molecule signals and the degraded poly(carbonate) backbone can be plotted over time (Figure 3C). Assuming first order kinetics of the polycarbonate hydrolysis, one can derive a half‐life of the polycarbonate of 3.9 d (Figure S47, Supporting Information), which corresponds well to our observations of aliphatic polycarbonate‐based nanocarriers with varying hydrophilicities in water.^[^ ^15^, ^31^, ^41^, ^42^, ^43^ ^]^ In contrast to these findings, no degradation products were observed for the non‐responsive control system (Figure S46B.1, Supporting Information). After nine days, both polymers were isolated and once more analyzed by ^1^H NMR spectroscopy, now dissolved in DMSO‐d_6_ as good solvent to resolve all protons of the samples. Only PEG signals were found for the hydrophilized polymer with degraded carbonate backbone (Figure S46A.2, Supporting Information). For the non‐responsive control on the other hand, all protons of the poly(MTC‐OBn) block were retained (Figure S46B.2, Supporting Information). GPC analysis confirmed these findings. While for the degraded carrier the GPC trace was congruent with the mPEG_113_‐OH trace (Figure S48, Supporting Information), only a minor shift toward smaller molecular weights was observed for the non‐responsive control sample (Figure S49, Supporting Information). Presumably, the hydrophilized poly(carbonate)s exhibit a greater affinity for water, thereby facilitating backbone hydrolysis. In contrast, the hydrophobic core of the intact polymeric micelles repels water, impeding poly(carbonate) hydrolysis. This could also be further confirmed by studying the thermal stability of the formulated benzyl ketal micelle in PBS at pH 7.4 (compared to side group degradable, non‐self‐assembling hydrophilic block copolymers) at elevated temperatures (Figure S50, Supporting Information), where no difference in scattering count rate could be found, which is advantageous for long‐term storages. Altogether, compared to other acid‐responsive systems, our nanocarrier not only enables stimulus responsive micelle degradation but also optimizes the backbone degradation profile. These findings go along well with the hydrolytic degradation profile of aliphatic polycarbonate nanocarriers sensitive to the local balance of hydrophobic‐to‐hydrophilic compositions.^[^ ^15^, ^31^, ^41^, ^42^, ^43^ ^]^ As for the here introduced micellar delivery system, the intrinsic pH‐sensitive ketal‐functionality provides an improved safety profile that guarantees hydrolytic biodegradation and eliminates the risk of undesired aggregate formation upon pH‐responsive nanoparticle unfolding.

Before testing the carriers drug delivery performances, it was important to assure the polymeric micelles stability under diluted conditions, as required for in vitro and in vivo experiments. Therefore, the critical micelle concentration (CMC) of both nanoparticle formulations was determined. For this purpose, pyrene was encapsulated into the polymeric micelles and its fluorescence was analyzed. The intensity ratio of the I_3_ and I_1_ emission bands depends on the polarity of the pyrene environment. This allows to distinguish whether pyrene is encapsulated in the hydrophobic core of a polymeric micelle or whether it is dissolved in the aqueous solvent as a result of block copolymer self‐assembly. A dilution series of pyrene‐loaded polymeric micelles was prepared in PBS, containing 0.6 µm pyrene and the fluorescence of these samples was measured. The ratio of the vibronic emission bands I_3_/I_1_ can be plotted against the nanoparticle concentration (*β*_NP_), as shown in Figure 3D. For the acid‐responsive block copolymer micelles, a CMC of 205 nmol L^−1^ (2.40 µg mL^−1^) was found (Figure S51C.1, Supporting Information), while for the non‐responsive control sample, a CMC of 65.6 nmol L^−1^ (0.674 µg mL^−1^) was determined (Figure S51C.2, Supporting Information). This small difference in CMC can be expected as the bulkier ketal side chain may lead to a less compact *π*–*π*‐stacking, as reported for other micelles with aromatic cores, resulting in a slightly higher CMC.^[^ ^44^, ^45^ ^]^ However, the nanomolar CMCs allow for applications of both carriers under diluted conditions. Disassembly should therefore only be caused by triggering the degradation mechanism of the acid‐responsive nanocarrier. This was further confirmed by acidification of the pyrene‐loaded polymeric micelles. For the non‐responsive control system, no significant change was observed (Figure 3D). Only in case of the acid‐responsive nanoparticles, the decreased pH value led to a concentration independent, low I_3_/I_1_ ratio confirming the acid‐responsive disassembly of these polymeric micelles.

Moreover, the morphology of the micellar self‐assemblies was evaluated by transmission electron microscopy (TEM). Spherical nanoparticles were found of similar sizes and narrow distributions could be identified (Figure S52, Supporting Information) in analogy to the collected dynamic light scattering measurements (Figure 3B). The diameters of randomly selected spherical particles were measured yielding an average particle size of 25 ± 5 nm (Figure S52, Supporting Information). In analogy to our previous findings,^[^ ^36^ ^]^ the particles appeared again slightly smaller compared to the dynamic light scattering (DLS) data. This may be attributed to solvent removal during transmission electron microscopy (TEM) sample preparation. Alternatively, limited contrast arising from the PEG corona of the micelles could contribute to this discrepancy. Nevertheless, both characterization methods provided comparable size distributions, indicating good agreement between the techniques.

### 2.4 Cell Uptake and Immune Stimulation

**Figure 4:**
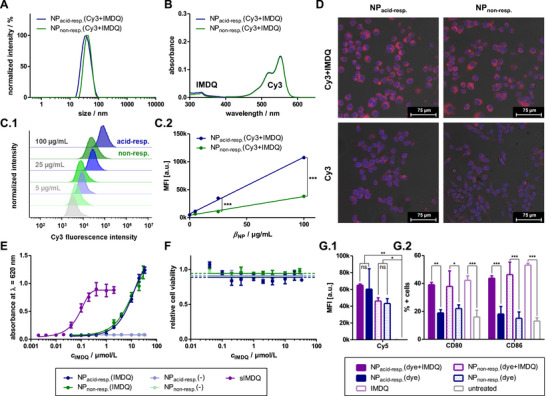
A) DLS measurement of Cy3+IMDQ‐loaded acid degradable nanoparticles (NP_acid‐resp._) and the non‐responsive control system (NP_non‐resp._). B) UV/vis absorption spectra (1:10 in DMSO) of the same nanoparticles showing comparable Cy3 and IMDQ loading. C) Flow cytometry results of RAW‐Blue macrophages incubated with Cy3+IMDQ‐labeled nanoparticles. In C.1 the Cy3 fluorescence intensity is shown for cells treated with IMDQ+Cy3‐labeled nanoparticles at three tested particle concentrations (100, 25, 5 µg mL^−1^). C.2) The mean fluorescence intensity shows a linear dependency on the particle concentration (*n* =  3, one way ANOVA with post‐hoc analysis *p* < 0.05, **p < 0.01, ***p < 0.001). D) Confocal fluorescence microscopy images of Cy3‐labeled nanoparticles with and without IMDQ loading. E) QUANTI‐Blue assay of macrophages incubated with IMDQ‐labeled nanoparticles and free IMDQ (*n* =  4). F)The viability of cells used for the QUANTI‐Blue^TM^ assay was analyzed by MTT assay (*n* =  4). G) Flow cytometry results of BMDCs incubated with acid degradable nanoparticles and the non‐responsive control system. In G.1, the particle uptake of Cy5‐labeled polymeric micelles with and without IMDQ‐loading is shown (*n* =  2, one way ANOVA with post‐hoc analysis *p* < 0.05, **p < 0.01). In G.2, the maturation of BDMC incubated with TAMRA labeled nanoparticles with and without IMDQ‐loading was evaluated based on the upregulation of CD80 and CD86 surface proteins (*n* =  3, one way ANOVA with post‐hoc analysis *p* < 0.05, **p < 0.01, ***p < 0.001).

Next, the micelles’ in vitro performance was tested in a series of cell experiments. Micelle uptake by RAW‐Blue macrophages was investigated by flow cytometry and confocal fluorescence microscopy. For this purpose, only Cy3‐labeled polymers of (**1**) and (**2**) were first formulated into micelles. To address the effect of adjuvant codelivery, Cy3‐ and IMDQ‐conjugated block copolymers were coformulated and tested. The formulations were prepared by mixing the modified block copolymers with non‐modified carrier material in acetone followed by the addition of sterile PBS. After acetone evaporation overnight, the self‐assembled micelle particles were filtered through sterile hydrophilized PTFE syringe filters (pore size 0.2 µm). An aliquot of each sample was analyzed by DLS confirming nanometer sized particles (**Figure** **4**A; Figure S54, Supporting Information). Drug and dye loading were evaluated by UV/vis spectroscopy (Figure 4B). For IMDQ‐conjugated samples a drug loading of 23.3 µmol L^−1^ (93% loading efficiency, acid‐responsive) and 23.2 µmol L^−1^ (93% loading efficiency, non‐responsive) was found (Figure S55A, Supporting Information). Furthermore, Cy3‐labeling was comparable for all nanoparticle samples (Figure S55B, Supporting Information). Cell uptake of all formulations was tested by treating macrophages at three different particle concentrations (100, 25, and 5 µg mL^−1^). Flow cytometry measurements revealed a dose‐dependent particle uptake, where a linear dependency between concentration and cell uptake could be derived for all samples. The co‐formulation of Cy3 and IMDQ‐modified polymers led to a stronger Cy3 fluorescence for the acid‐responsive system compared to the non‐responsive control, indicating an improved internalization for the acid‐responsive nanocarrier (Figure 4C). Altogether, for both carriers the IMDQ co‐delivery led to a more pronounced cell uptake compared to the samples without adjuvant (Figure S56C, Supporting Information). This trend is consistent with earlier observations^[^ ^15^, ^46^ ^]^ and can be explained by immune cell activation and receptor upregulation, resulting in accelerated endocytosis. Our findings could be further confirmed by confocal fluorescence microscopy revealing an internalization of the micelles inside cells (Figure 4D; Figure S57, Supporting Information). Again, a more pronounced Cy3 signal was found for IMDQ‐conjugated micellar nanoparticles compared to those micelle formulations without the adjuvant.

Driven by these results, we performed a QUANTI‐Blue assay to evaluate the capacity of the IMDQ‐loaded formulations to activate the NF‐κB pathway of the RAW‐Dual macrophages. For this purpose, the acid‐responsive nanocarrier and the non‐responsive control system were formulated with and without IMDQ‐conjugation and characterized by DLS and UV/vis spectroscopy (Figure S58, Supporting Information). As reference, soluble IMDQ was used. Compared to free drug, a decreased immune activity was found for both IMDQ‐loaded formulations (Figure 4E). However, the two IMDQ‐loaded nanoformulations enhanced the NF‐κB activity at higher doses. Altogether, they are able to stimulate immunity to a comparable extent within the micromolar range, similar to other reported covalent IMDQ nanoformulations.^[^ ^4^, ^41^, ^46^, ^47^, ^48^, ^49^ ^]^ The reduction in IMDQ activity that has also been reported for those other nanoformations before may be attributed to the chemical modification necessary for its covalent attachment to the block copolymer chain end, but makes the conjugates still highly suitable for in vivo applications. Besides, the immune stimulation assay showed no intrinsic activity of the unloaded carrier materials, confirming their absence of intrinsic immnostimulatory properties.

In addition to assessing the nanoparticles’ immunostimulatory potential, the cells’ viability was tested by performing an MTT assay (Figure 4F). For both carriers, no cytotoxicity was observed within the tested concentration range, regardless of IMDQ‐loading, underlining the high compatibility of the nanoformulations for biological applications.

To get a deeper insight into the nanocarriers drug delivery performance, we investigated the micelles’ interaction with primary immune cells and checked for uptake and immune cell stimulation using murine bone marrow‐derived dendritic cells (BMDCs). For this purpose, Cy5‐ and Cy5+IMDQ‐labeled nanoparticles were formulated from both carriers and first investigated for their cell uptake (Figure S59, Supporting Information). As reference, soluble IMDQ was used. The samples and the reference were added to BMDC cell suspensions at a final IMDQ concentration of 4 µmol L^−1^. Unloaded micelles were used analogously. After incubating BMDCs with nanoparticle samples and reference respectively, uptake was evaluated by flow cytometry. For cells treated with Cy5 labeled nanoparticles, a distinct shift in the Cy5 mean fluorescence intensity (MFI) was found, confirming pronounced particle uptake (Figure 4G.1). At the same time, the IMDQ reference and untreated cells gave very low signals, confirming that the increased MFI was a result of nanoparticle uptake. Compared to the previously investigated particle uptake by macrophages, no difference was found between IMDQ‐loaded and unloaded micelles, highlighting the individual behavior of different cell types. To that respect, variations in TLR 7 expression have been reported in literature for macrophages versus dendritic cells, both on mRNA levels as well as by surface receptor affinity of anti‐TLR 7 antibodies, that could explain why the IMDQ‐loaded acid‐responsive benzyl ketal functionalized micelles provides preferentially higher uptake into macrophages.^[^ ^50^, ^51^ ^]^

Next, IMDQ‐triggered maturation of BMDCs was determined by evaluating the upregulation of CD80 and CD86 surface markers (Figure 4G.2). For that purpose, cells were incubated with TAMRA‐ and TAMRA+IMDQ‐labeled nanoparticles at an IMDQ concentration of 5 µmol/L (Figure S60, Supporting Information). According to flow cytometry analyses, only IMDQ‐loaded samples induce significant upregulation of CD80 and CD86 (Figure 4G.2). As these surface proteins are crucial for co‐stimulating T cells, the observation suggests potential activation of the adaptive immune system within complex biological environments. Again, no difference between acid‐responsive and non‐responsive nanoparticles was observed. In contrast to the results from the QUANTI‐Blue assay, which suggested a reduced immune activation conferred by IMDQ formulations, a comparable immune cell maturation was found for micelle‐mediated IMDQ delivery and the soluble drug, while micelles without IMDQ exerted no stimulatory effect. In addition to surface marker maturation, cytokine secretion was evaluated from the cell culture supernatants using a cytometric bead array (CBA). A distinctive upregulation was found for cells treated with IMDQ‐containing formulations yielding an upregulation of TNF‐α and IL‐6 secretion, underlining the potency of the adjuvant and the successful micelle‐mediated delivery for the immunomodulating drug (Figure S61, Supporting Information).

Overall, these experiments demonstrate the capability of both block copolymer end group modified nanocarriers to deliver the active immunostimulatory cargo into different immune cells and to induce their maturation effectively. The acid‐responsive and backbone degradable block copolymer micelle is as potent as the non‐responsive carrier system but with superior unfolding features.

### 2.5 Endocytosis‐driven Intracellular Micelle Unfolding

**Figure 5:**
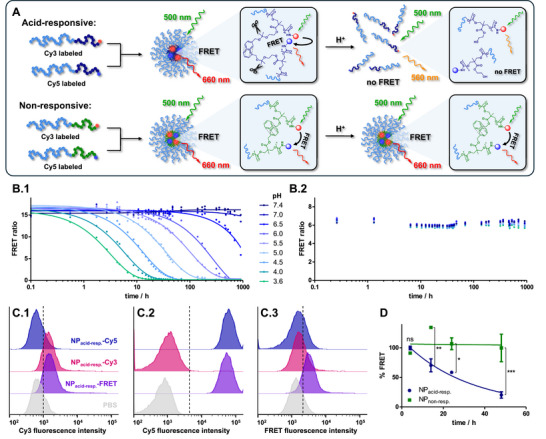
FRET studies for intracellular unfolding of acid‐responsive nanoparticles (NP_acid‐resp._) bearing ketal side groups. A) Schematic overview of Cy3‐ and Cy5‐labeled block copolymers used as FRET probes to evaluate micelle unfolding upon acidification of acid‐responsive nanoparticles, while the non‐responsive control micelles remain stable. B) Time dependent particle degradation study at different pH levels for acid‐responsive nanoparticles (B.1) and the non‐responsive control system (B.2). C) Flow cytometry fluorescence intensity histograms of macrophages treated with Cy5, Cy3 or FRET pair‐labeled acid‐responsive nanoparticles next to macrophages treated with PBS. The different channels displayed in C.1–C.3 show a shift of the histograms as expected for respective dye‐labeling. D) Time dependent FRET degradation in macrophages. For acid‐responsive nanoparticles a declining FRET ratio was found over time, indicating intracellular particle unfolding upon endocytosis while the non‐responsive control system remains intact (*n* =  3, one way ANOVA with post‐hoc analysis *p* < 0.05, **p < 0.01, ***p < 0.001).

To demonstrate the responsiveness of the nanocarrier system with ketal side groups in a biological environment, its endocytosis‐driven micellar unfolding was investigated in macrophages. For this purpose, the fluorophores Cy3 and Cy5 were used as FRET probe to monitor particle disassembly. The co‐formulation of polymers individually labeled with one of these dyes into micellar nanoparticles leads to a FRET that can be analyzed by fluorescence spectroscopy (**Figure** **5**A). In general, excitation of such micelles at a Cy3 specific wavelength (≈500 nm) affords the nonradiative transfer of the absorbed energy to the acceptor dye, yielding a Cy5 characteristic emission (≈600 nm). For the acid‐responsive nanoparticles, the acidification induces micelle unfolding and the fluorophores separate affording a decrease in FRET efficiency. After complete disassembly, only the Cy3 characteristic emission (≈560 nm) remains. In contrast, acidification of the non‐responsive system does not affect the micellar integrity, and the FRET signal remains intact. Such FRET‐based monitoring is highly valuable for quantifying micellar integrity during cell uptake via flow cytometry measurements.

To evaluate the pH‐responsiveness of the FRET pair‐labeled polymers, we first formulated them into micelles in water at a carrier concentration of 1.0 mg mL^−1^. To exclude interference by direct acceptor dye excitation or fluorescence overlap of the two dyes, single dye‐labeled micelles were tested as well. Initially, fluorescence emission measurements were performed in a black 96‐well plate using a TECAN Spark plate reader. To analyze particle stability at multiple pH values, different buffers were used (PBS: pH 7.4–6.5, or acetate buffer: pH 5.5–3.6). For each sample and pH value, 180 µL of the respective buffer were added to the well plate in triplicates. Afterwards, 20 µL of sample solution were added, leading to a final particle concentration of 0.1 mg mL^−1^. Immediately after mixing, fluorescence spectra were measured (*λ*_ex_  =  500 nm). For both carriers, FRET pair‐labeled samples exhibit intense Cy5 emission at all tested pH values (Figure S62A.1+B.1, Supporting Information). In contrast, Cy3 only‐labeled samples emitted a Cy3 characteristic emission profile (Figure S62A.2+B.2, Supporting Information), while Cy5 only‐labeled nanoparticles revealed no significant fluorescence (Figure S62A.3+B.3, Supporting Information). These observations allow to clearly distinguish between FRET and Cy3 emission and exclude any direct Cy5 excitation at 500 nm excitation wavelength. To investigate the stability of the FRET pair‐labeled nanoparticles under varying conditions, fluorescence measurements were repeatedly conducted over 38 d. The FRET ratio was calculated from the emission maxima at 676 and 568 nm and plotted against time (Figure 5B). For the acid‐responsive nanoparticles, a gradual reduction of the FRET ratio was observed in acidic environments (Figure 5B.1). The complete disappearance of the FRET signal took ≈10 h at pH  3.6 and gradually slowed down in milder environments. At physiological conditions (pH  7.4), the nanocarriers remained fully stable over 38 d. In contrast, for the non‐responsive carrier system, a constantly high FRET ratio was found at all tested pH levels over the whole measurement period (Figure 5B.2). These findings are in line with previous observations and confirm the stimulus responsiveness of the acid‐responsive carrier system.^[^ ^15^, ^36^ ^]^ Moreover, having a closer look at the FRET‐derived rate constants for each pH value, one can confirm once more that the observed ketal hydrolysis follows a single‐proton‐catalyzed hydrolysis mechanism, as earlier reported (Figure S63, Supporting Information).^[^ ^36^ ^]^ The obtained degradation kinetics further correspond well to the ^1^H NMR degradation studies conducted in D_2_O under acidic conditions, too (Figure S41, Supporting Information) and, thus, demonstrate the suitability of the FRET probe to evaluate the integrity and decomposition of polymeric micelles.

Before testing endocytosis‐driven intracellular particle unfolding in macrophages, it was necessary to get a better insight into the micelles’ stability and to confirm their behavior in complex biological environments. Since polymeric micelles are not covalently stabilized, polymer chains might be in an equilibrium between assembled and freely dissolved state. Therefore, the exchange of single polymer chains between individual polymeric micelles was investigated to get insight into these dynamics. For this purpose, Cy3 and Cy5 single dye‐labeled micelles were formulated in PBS (pH 7.4) separately. The obtained nanoparticles were then combined, yielding a mixture of single dye‐labeled micelles that did not exhibit a FRET signal. Only upon polymer chain exchange between the micelles, FRET would occur over time. As reference, dual dye‐labeled polymeric micelles were also analyzed. As depicted in Figure S64 (Supporting Information), fluorescence measurements revealed a strong Cy3 emission for the mixture of single dye‐labeled polymeric micelles (*λ*_ex_  =  500 nm), resulting in a low FRET ratio. Compared to this, a high FRET ratio was found for the FRET pair‐labeled micelles. In between the measurements, the samples were stored at 37 °C. Remarkably, over time the low FRET ratio was preserved for the mixed micelles, thus, no significant polymer chain exchange between individual micelles was observed, even after one week (Figure S64C, Supporting Information). This indicates a high stability of acid‐responsive and non‐responsive nanocarriers at physiological conditions. Subsequently, micelle stability was further tested in blood plasma. For this purpose, FRET pair‐labeled nanoparticles were incubated in human blood plasma at 37 °C (Figure S65, Supporting Information). It was found that both carriers are comparably stable as they exhibited pronounced FRET ratios even after one week. Acidification with 10vol% hydrochloric acid (1 m) led to a reduced but still distinct FRET ratio for the non‐responsive nanoparticles. In stark contrast, the FRET ratio of the acid degradable nanocarrier dropped to near zero immediately upon acidification, confirming its rapid degradation also in more complex environments (Figure S65C, Supporting Information).

Based on these observations, we were now able to monitor whether an intracellular nanocarrier unfolding would occur upon endocytosis by recording the FRET ratio. To verify that our flow cytometry settings are applicable, RAW‐Blue macrophages were first incubated with single dye‐labeled and FRET pair‐labeled nanoparticles (Figure S66A–B, Supporting Information). As shown in Figure 5C for the acid degradable nanoparticles (and in Figure S66C (Supporting Information) for non‐responsive nanoparticles), the fluorescence intensity distributions detected in the individual flow cytometry channels provided only signals according to each dye‐labels as expected. These measurements also confirmed the distinctiveness of the single dye signals from the FRET signals by our flow cytometry setup, thus, providing the basis for the subsequent time‐dependent experiments. In a follow‐up experiment, the FRET pair‐labeled particles could therefore be monitored over time (Figure S67A,B, Supporting Information). RAW‐Blue macrophages were pulsed with acid‐responsive and non‐responsive FRET pair‐labeled nanoparticles for 4 h. Subsequently, the cells were washed with 1.0 mL PBS. Afterwards, cells were either analyzed directly by flow cytometry or incubated with 1.0 mL fresh cell culture medium for another 10, 20, or 44 h. The monitored fluorescence signals declined in all flow cytometry channels over time (Figure S67C,D, Supporting Information), which is a result of progressing cell division associated with a reduced number of nanoparticles per cell. To assess the integrity of the micelles inside the macrophages, the FRET ratio was therefore calculated from the FRET channel MFI divided by the Cy3 channel MFI (for both channels the MFI of PBS‐treated cells was subtracted first). Figure 5D shows the progression of the FRET ratio for acid degradable nanoparticles compared to non‐responsive nanoparticles. While the non‐responsive control system exhibited a constantly high FRET ratio, a gradual decay in the FRET ratio was found for the acid‐responsive nanoparticles. In analogy to our previous experiments at endosomal pH conditions (Figure 5B), these observations clearly indicate particle unfolding upon endocytosis exclusively for the acid‐responsive nanocarrier system, underlining the superiority of this drug delivery platform. These observations suggest an improved drug accessibility in immune cells and a better safety profile of the acid‐responsive nanocarrier according to its degradation in the target environments.

### 2.6 In Vivo Particle Distribution, Degradation, and Immunostimulation

To test whether endocytosis‐driven micellar unfolding of acid‐responsive nanoparticles also occurs in vivo, further experiments were conducted to investigate the particle distribution and degradation in mice. Since visible light at low wavelengths cannot penetrate the skin of test animals, the previously used FRET pair, Cy3/Cy5, was not suitable for such experiments. Therefore, we decided to couple Cy5 and the near infrared fluorescent dye IRDye800RS to the end groups of the block copolymers (**1**) and (**2**), as this combination enables excitation by higher wavelengths which better penetrate through the skin.^[^ ^52^ ^]^

We therefore co‐formulated the acid‐responsive Cy5‐ and IRDye800RS‐labeled block copolymers into micelles and first excited them with the Cy5 characteristic excitation wavelength (*λ*_ex_  =  550 nm) and, indeed, we observed a substantially reduced emission of the Cy5 donor dye in analogy to the FRET couple before. However, the anticipated emission of the IRDye800RS remained absent (Figure S68, Supporting Information). To that respect the near infrared dye does not serve as typical acceptor for FRET‐based emission but rather as highly effective FRET‐quencher that completely blocks the fluorescence when both dyes are in close proximity. This quenching is reversible, since acidification of the particle sample immediately resulted in Cy5 fluorescence recovery due to micellar unfolding. Thus, the Cy5‐ and IRDye800RS dye pair can be used to assess micelle integrity in vivo based on the extent of Cy5 quenching (Figure S68, Supporting Information). We hypothesize that this is indeed a more valuable tracer for in vivo micellar unfolding because of its sensitive positive evolution of the Cy5 signal which could otherwise not occur after in vivo administration.

To further prove this property, we repeated the cell uptake of the micelles into RAW‐Blue macrophages, but now applying the single Cy5 and IRDye800RS dye labeled micelles as well as the double labeled FRET‐quencher system (Figure S69, Supporting Information). After incubation for 24 hours, we recorded the fluorescence of the Cy5 dye and the IRDye800RS (Figures S70–S72, Supporting Information). Consistent with our previous observations, selective recovery of the Cy5 fluorescence was exclusively detected in the cells treated with the acid‐labile benzyl ketal micelles, whereas the non‐responsive benzyl ester control samples exhibited sustained quenching of Cy5 emission (Figures S70,S71, Supporting Information). These results provide compelling evidence that, under the applied intracellular conditions, the benzyl ketal micelles undergo acid‐triggered disassembly, facilitating the dissociation of Cy5‐labeled polymers from the IRDye800RS‐containing polymers. Confocal laser scanning microscopy further revealed that the extent of Cy5 fluorescence recovery within cells approached levels observed in control samples containing only Cy5‐labeled polymers (Figure S72, Supporting Information). This finding is in agreement with our flow cytometry data obtained from FRET‐labeled micelles, which indicated that ≈50% of the micelles underwent successful disassembly and fluorophore separation (Figure 5D). Altogether, these observations confirm the functional integrity and intracellular responsiveness of the acid‐cleavable micellar system.

Subsequently, in vivo particle distribution and degradation experiments could be conducted as comparative study for examining the difference between the acid‐responsive micellar nanoparticles and the non‐responsive control system. For both carriers, three groups were prepared. The first group consisted of polymeric micelles labeled with Cy5 only (a), while for the second group the micelles were labeled with Cy5 and IRDye800RS (b), and the third group included micelles labeled with Cy5 and the TLR7/8 agonist IMDQ (c). This sample set allowed for comprehensive comparison of the two carrier systems in terms of their in vivo distribution (a), micellar unfolding and biodegradation (b), and drug delivery performance (c). For all samples the overall carrier concentration that was applied to mice was 4.0 mg mL^−1^, with targeted Cy5 labeling of 50 µmol L^−1^. For IRdye800RS‐labeled samples, an equimolar dye loading was targeted (50 µmol L^−1^) while for IMDQ‐conjugated samples an adjuvant loading of ≈50 µg mL^−1^ could be achieved. As references a mixture of soluble Cy5 and soluble IMDQ was applied, as well as PBS. To ensure comparable dye and drug loading, all samples were carefully analyzed by UV/vis spectroscopy (Figure S69, Supporting Information).

**Figure 6:**
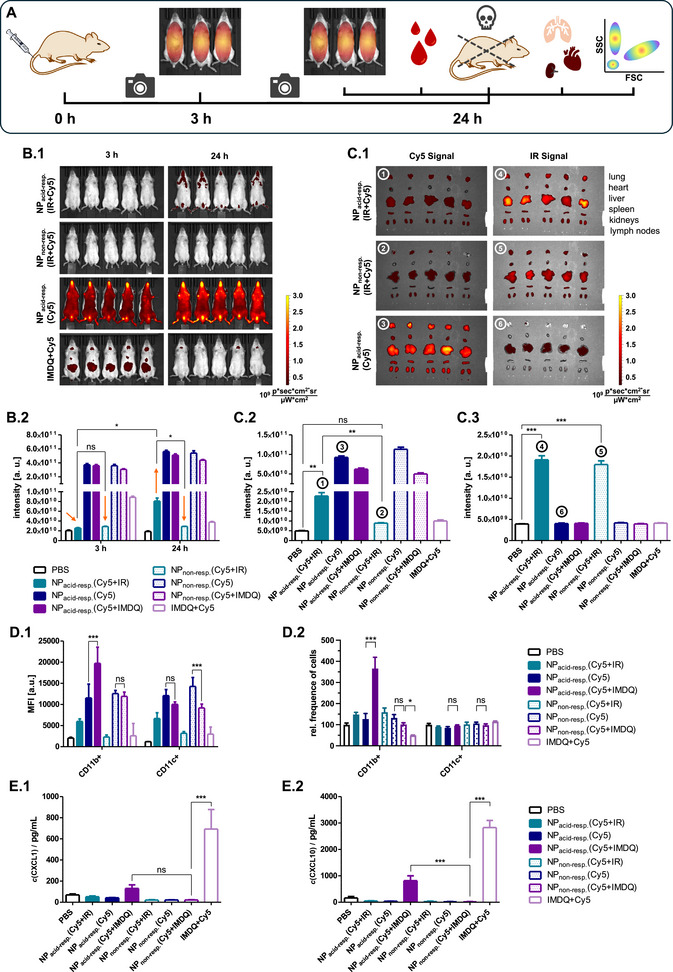
In vivo distribution, degradation and immunostimulation study. A) Schematic illustration of the experimental setup. Samples were injected intravenously into the tail vein of 5–8‐week‐old male C57BL/6N‐Tyrc‐Brd/BrdCrCrl mice. After 3 and 24 h whole body IVIS images were recorded. After 24 h the mice were sacrificed, blood samples were taken, and major organs were harvested. Splenocytes were analyzed by flow cytometry. B) Selected IVIS images of whole animals after 3 and 24 h (B.1) and the quantification of Cy5 radiant efficiency after 3 and 24 h (B.2) (*n* =  5, PBS group *n* = 3, one way ANOVA with post‐hoc analysis *p* < 0.05, **p < 0.01, ***p < 0.001). C) Selected IVIS images of major organs (C.1) and the quantification of the Cy5 radiant efficiency (C.2) as well as the IRdye800RS radiant efficiency (C.3) in spleens (*n* =  5, PBS group *n* = 3, one way ANOVA with post‐hoc analysis *p* < 0.05, **p < 0.01, ***p < 0.001). D) Flow cytometry results of splenocytes showing the Cy5 mean fluorescence intensity (MFI) in different subpopulations (D.1) and the relative frequency of immune cells found in the spleen after treatment (D.2) revealing that the acid‐responsive nanoparticles preferentially increase the number of CD11b+ immune cells and are favorably taken up by these immune cells, too (*n* =  5, PBS group *n* = 3, one way ANOVA with post‐hoc analysis *p* < 0.05, **p < 0.01, ***p < 0.001). E) Secretion of selected cytokines CXCL1 (E.1) and CXCL10 (E.2) revealing reduced activity of the nano‐formulations compared to free IMDQ but some activity recovery in case of acid‐responsive nanoparticles (*n* =  5, PBS group *n* = 3, one way ANOVA with post‐hoc analysis *p* < 0.05, **p < 0.01, ***p < 0.001).

All samples were injected intravenously (i.v., 150 µL) into the tail vein of 5–8‐week‐old male B6‐albino mice (C57BL/6N‐Tyrc‐Brd/BrdCrCrl) (*n* = 5; PBS ref. *n* = 3), following the approval by the local ethics authorities (Landesuntersuchungsamt (LUA) Rhineland‐Palatinate, Germany) under the reference number G 20‐1‐123. Mice were imaged using an in vivo imaging system (IVIS) at predetermined timepoints 3 and 24 h following i.v. injection. To analyze both, the in vivo distribution of the micelles and their integrity, the IRdye800RS fluorescence (*λ*_ex_  =  745 nm, exbw  =  20 nm, *λ*_em_  =  820 nm, embw  =  20 nm) as well as the Cy5 emission (*λ*_ex_  =  640 nm, exbw  =  20 nm, *λ*_em_  =  680 nm, embw.: 20 nm) were detected. After the last IVIS imaging at 24 h, blood was collected via the facial vein and the mice were sacrificed to harvest major organs. In order to quantify individual organ‐fluorescence, lungs, hearts, livers, spleens, kidneys and inguinal lymph nodes were imaged subsequently. Furthermore, the spleen as a major site of endocytically active immune cells was processed for flow cytometry analysis, and the blood samples were analyzed for their cytokine levels (**Figure** **6**A).

In Figure 6B.1 selected IVIS images are shown for mice treated with dual dye‐labeled acid‐responsive nanoparticles (NP_acid‐resp._(Cy5+IR)) and the non‐responsive control samples (NP_nono‐resp._(Cy5+IR)) next to mice treated with Cy5 only‐labeled acid‐responsive nanoparticles (NP_acid‐resp._(Cy5)) or the soluble Cy5 and IMDQ reference (Cy5+IMDQ). In case of the administration of Cy5 only‐labeled particles the IVIS images showed strong Cy5 fluorescence after 3 h. This signal remained stable for 24 h, whereas the signal for the soluble dye rapidly vanished due to fast renal clearance. This highlights the prolonged circulation time of the PEGylated micelle formulation, which was also observed for the non‐responsive control formulation (Figure S73, Supporting Information), corroborating their advantageous in vivo stealth‐like circulation properties.

In case of the co‐formulated Cy5 and IRdye800RS micelles, the Cy5 fluorescence was completely quenched at 3 h, emphasizing the particles integrity in the blood stream. After 24 h, however, some Cy5 fluorescence emission was detected for the acid‐responsive carrier system suggesting successful micellar particle unfolding in vivo. In comparison, no Cy5 emission was found for the non‐responsive control sample, highlighting the unique degradation in vivo features of the micelles with ketal side chains. The IR signal remained stable for both particles confirming preserved blood circulation (Figure S73, Supporting Information). The extent of the Cy5 signal recovery was quantified based on the IVIS images in Figure 6B.2. The average radiant efficiency revealed a consistently high Cy5 signal for Cy5 only‐ and Cy5+IMDQ‐labeled particles after 3 and 24 h, respectively. In contrast, the co‐formulation of Cy5 and IRDye800RS resulted in complete Cy5 quenching after 3 h, reducing the signal to the level of the PBS control (orange arrows). For the acid‐responsive sample, however, this value increased threefold within the subsequent 21 h, while it remained constantly low for the non‐responsive control sample. These observations indicate sustained micelle unfolding and nanoparticle degradation exclusively for the acid‐responsive nanoparticles.

In Figure 6C.1 selected IVIS images of the harvested organs are shown for the dual dye‐labeled acid‐responsive nanoparticles (NP_acid‐resp._(Cy5+IR)) and the non‐responsive control sample (NP_non‐resp._(Cy5+IR)) next to Cy5 only‐labeled acid‐responsive nanoparticles (NP_acid‐resp._(Cy5)). Nanoparticle distribution was evaluated using the Cy5 and the IRDye800RS emission. The Cy5 signal of Cy5 only‐labeled particles revealed typical particle accumulation in lung, heart, liver, spleen and kidneys as highly vascularized organs while only minor signals were found in lymph nodes (also see Figure S74, Supporting Information). These data underline the micelles’ prolonged circulation in the blood stream within the observed time frame. For the dual dye‐labeled particles, the Cy5 signal was mostly quenched but some signal recovery was found for the acid‐responsive system. Cy5 signal recovery was particularly pronounced in organs bearing many endocytic immune cells (lung, liver, spleen – compare Figure S75, Supporting Information for quantification).

In Figure 6C.2–3 this signal recovery is quantified for the spleen as major site of endocytic immune cells (numbers assign the respective IVIS image in Figure 6C.1). While the IR signal suggests equal cell uptake of both micellar nanocarrier systems (Figure 6C.3), a substantial Cy5 recovery was found for acid‐responsive nanoparticles exclusively (Figure 6C2). However, the Cy5 signal was still reduced compared to single dye‐labeled samples. This suggests incomplete particle degradation after 24 h, which is in agreement with our in vitro data (Figure 5) and supposed to continue (mice had to be sacrificed at that time point according to the study protocol approved by the local animal welfare authority), corroborating the sustained nanoparticle degradation and cargo release. In organs that typically provide less endocytic immune cells (such as heart and kidneys), the Cy5 signal of both dual dye‐labeled nanoparticles remained mostly quenched (Figure S75, Supporting Information). This observation reinforces the hypothesis that acid‐responsive micelle unfolding can only occur upon cell uptake (by endocytic immune cells). Furthermore, the solube IMDQ+Cy5 reference sample provided only some Cy5 emission at early time points, indicating fast renal clearance of the small molecules, as now found by some remaining fluorescence in the kidneys (Figure S75, Supporting Information), underlining the necessity of a nanoparticulate delivery platform to increase the small molecule drugs’ bioavailability.

In order to finally evaluate immune cell uptake of the micelle formulations and activation by IMDQ delivery, splenocytes were analyzed by flow cytometry (Figure S76, Supporting Information). Cell uptake was evaluated by quantifying the Cy5 mean fluorescence intensity. Particle uptake was most dominant in CD11b^+^ immune cells, primarily representing macrophages and neutrophils, and CD11c^+^ immune cells, representing dendritic cells (DCs) (Figure 6D.1). Within the CD11b^+^ subpopulation, Cy5 only‐labeled particles were taken up comparably well for both carrier systems. Also, the Cy5+IMDQ co‐labeled non‐responsive control sample showed comparable cell uptake. In contrast, the Cy5+IMDQ co‐labeled acid‐responsive nanoparticles exhibited an almost 2‐fold increased particle uptake, which may be associated with an accelerated endocytosis triggered by immune activation, as also observed for macrophages in vitro (Figure 4). This confirms an increased IMDQ activity only in case of delivered by the acid‐responsive nanoparticles, reinforcing improved drug accessibility upon particle degradation. The hypothesis can further be supported by evaluating the Cy5 MFI of the dual dye‐labeled particles. It is most likely that these particles were taken up in a comparable extent like the Cy5 only‐labeled particles. However, for the non‐responsive control system the Cy5 fluorescence remained mostly quenched while for the acid‐responsive nanocarrier Cy5 fluorescence increased suggesting particle degradation. The uptake by CD11c^+^ DCs revealed a slightly different picture. Although comparable uptake was found for the two carriers with Cy5 labeling, no influence of IMDQ co‐delivery was observed. This is consistent with our in vitro results, which also showed no accelerated particle uptake into BMDCs upon immune stimulation (Figure 4). However, also for DCs particle degradation was observed for the acid‐responsive nanocarrier according to the Cy5 recovery compared to the non‐responsive control sample (Figure 6D.1). As suggested already before, the enhanced uptake by macrophages could be explained by the slightly higher TLR 7 expression and surface accessibility levels reported in literature for macrophages versus dendritic cells.^[^ ^50^, ^51^ ^]^ Moreover, these observations suggest that also an enhanced sensitivity toward TLR stimulation would be provided by CD11b^+^ versus the CD11c^+^ immune cells, which has been reported for human macrophages compared to dendritic cells by downstream processes including cytokine secretions.^[^ ^53^ ^]^

Such beneficial downstream impact on the immunostimulation of the CD11b^+^ immune cells in the spleen of mice treated with the Cy5+IMDQ co‐labeled acid‐responsive nanoparticles could further be evaluated by counting the relative frequency of these immune cell populations (Figure 6D.2). There is no difference observed for the CD11c^+^ immune cells for all samples, however, the Cy5+IMDQ co‐labeled acid‐responsive nanoparticles exclusively increase the number of CD11b^+^ immune cells by a factor of four, while all other samples leave also the CD11b^+^ immune cell population unaffected, except for the soluble IMDQ+Cy5 treated mice, where the frequence of the CD11b^+^ immune cells, primarily representing macrophages and neutrophils, is almost decreased by a factor of two. The latter suggests an unregulated systemic inflammatory toxicity of the free IMDQ with the spleen liberates those immune cells, while only the unfolding benzyl ketal micelles clearly focus the activity and recruitment of CD11b^+^ immune cells into the spleen exclusively (Figure 6D.2).

To better understand the improved immunostimulatory delivery performance for the acid‐responsive micelles, we finally monitored the secretion of inflammatory cytokines for mice treated with the different in vivo formulations by analyzing their sera (Figure 6E). Mice treated with the soluble Cy5+IMDQ reference exhibited significantly elevated levels of CXCL1 and CXCL10. While CXCL1 is important for the recruitment of neutrophils, CXCL10 plays a role in recruiting T cells, and natural killer cells, and also has anti‐infection and anti‐tumor functions.^[^ ^54^, ^55^ ^]^ Together, these cytokines orchestrate a robust immune response, coordinating innate and adaptive immunity. For the particle formulations, reduced cytokine secretion was observed. Remarkably, only the IMDQ‐loaded acid‐responsive nanoparticles revealed elevated cytokine levels, albeit lower than for the soluble drug reference. Along with the previously observed selective CD11b^+^ immune cell recruitment to the spleen, as well as the reported higher sensitivity of these immune cells toward TLR stimulation,^[^ ^53^ ^]^ this clearly indicates sustained drug activity. To that respect, additional cytokine parameters could be further evaluated from the mice blood sera, however, most of these still remained below the detection range for the mice treated with the block copolymers, except again for groups treated with the soluble Cy5+IMDQ reference (where elevated levels of INFγ, TNFα, CCL2, CCL5, IL‐6 and INFα/β were found – compare Supporting Information Tables S1–S3, Supporting Information). Altogether, both nanoparticle formulations appear biocompatible and mediate advanced IMDQ delivery by overcoming systemic side effects associated with the application of the small molecule drug in soluble form. However, only the acid‐responsive micelles retain bioactivity by improving pharmacokinetics and enabling safer administration.

In conclusion, the in vivo distribution, degradation and immunostimulation studies reveal prolonged particle circulation in the blood stream for at least 24 h. Carrier unfolding and degradation seem to be crucial for drug activity and were observed only for the acid‐responsive nanoparticles. The improved drug accessibility resulted in increased recruitment and particle uptake, particularly by CD11b^+^ splenocytes, and the upregulation of cytokine secretion, however, still at moderate levels that avoid the risk of undesired side effects.

Taking into account the modular approach of the here introduced block copolymer system, also with respect to heterotelechelic block copolymer end group modifications, further strategies for co‐delivery of, e.g., peptide‐based antigens can be considered, too. This would be achieved, for instance, by surface decoration to the nanoparticles via click chemistry approaches, as earlier reported by us for polycarbonate nanogel guided vaccinations,^[^ ^42^ ^]^ and allow eliciting antigen‐specific immune responses for anticancer immunization.

## 3 Conclusion

Our comprehensive investigation on the ketal‐side chain functionalized block copolymers affording acid‐responsive unfolding nanocarriers and their thorough comparison with a non‐responsive micellar aliphatic polycarbonate control system demonstrated the acid‐responsive nanocarrier's superiority with regards to its degradation profile and improved drug availability. Beyond acidic pH‐triggered side group decomposition initiating polymeric micelle degradation, a subsequent carbonate backbone hydrolysis of the hydrophilized chains can be observed, preventing undesired particle accumulation. The particles biodegradability emphasizes the safety profile of our acid‐responsive design, making these particles promising candidates for systemic biomedical applications. Furthermore, while the particles exhibit stimulus‐responsive lability, they still remain fairly stable under physiological conditions, achieving an optimal balance between durability and degradability. Even under diluted conditions as required for systemic in vivo applications, the polymeric micelles remained intact due to their low critical micelle concentration.

The covalent conjugation of dye probes to the block copolymer chain ends provided access to detailed analysis of the micellar self‐assembly and unfolding properties, even in complex biological environments. Co‐formulation of Cy3‐ and Cy5‐labeled block copolymers enabled the evaluation of the micelles’ integrity based on their FRET signal. This approach facilitated in vitro analyses, confirming the concept of an endocytosis‐driven particle unfolding. To translate this approach to in vivo applications, we co‐formulated Cy5‐ and IRDye800RS‐labeled block copolymers. Here, the near infrared dye afforded a Cy5 fluorescence quenching, indicating micelle stability and integrity, while fluorescence recovery signaled acid‐triggered micelle disassembly. Consequently, the in vivo performance regarding particle distribution and degradation could be investigated. The acid‐responsive micelles provided prolonged blood stream circulation in analogy to the non‐responsive control system, but revealed unique immune cell uptake‐mediated decomposition. Furthermore, co‐delivery of the covalently attached TLR7/8 agonist IMDQ to the block copolymers’ end group led to substantial immune stimulation in vitro and in vivo. In vivo drug activity was only observed upon delivery by the acid‐responsive nanocarrier. Thus, drug activity can be linked to immune cell uptake and particle degradation.

In conclusion, the acid‐responsive nanocarrier presented here serves as a valuable delivery platform that becomes biodegradable and helps prevent undesired side effects. Additionally, covalent conjugation and shielding of the immunodrug reduce systemic activity, while acid‐responsive degradation enables targeted adjuvant release at sites of interest.

## Conflict of Interest

The authors declare no conflict of interest.

## Supporting information



Supporting Information

## Data Availability

The data that support the findings of this study are available in the supplementary material of this article.
