# Supplementary material for: Unfolding and Degradation of Micellar Immunodrug Carriers Derived From End Group Modified Aliphatic Poly(Carbonate)s with Acid‐Responsive Ketal Side Groups
Source: Adv Mater. 2025 Dec 12;38(8):e11752. doi: 10.1002/adma.202511752 (PMC12879277; doi:10.1002/adma.202511752)
Supplement: Supplementary file 1 — Supporting Information [file ADMA-38-e11752-s001.pdf]

ADVANCED MATERIALS

Supporting Information

for *Adv. Mater.*, DOI 10.1002/adma.202511752

Unfolding and Degradation of Micellar Immunodrug Carriers Derived From End Group Modified Aliphatic Poly(Carbonate)s with Acid-Responsive Ketal Side Groups

*Adrian V. Hauck, Michael Fichter, Laura J. Rosenberger, Jannis Willig, Alexander Fuchs, Felicia Schön, Paul Schneider, Carolina Medina-Montano, Taufiq Ahmad, Jessica Erlenbusch, Pol Besenius, Leonard Kaps, Matthias Bros, Stephan Grabbe, Volker Mailänder, Jürgen Groll and Lutz Nuhn**

Supporting Information

Unfolding and Degradation of Micellar Immunodrug Carriers Derived from End Group Modified Aliphatic Poly(Carbonate)s with Acid-Responsive Ketal Side Groups

Adrian V. Hauck[1], Michael Fichter[2], Laura J. Rosenberger[2], Jannis Willig[1], Alexander Fuchs[1], Felicia Schön[2], Paul Schneider[2], Carolina Medina-Montano[2], Taufiq Ahmad[1], Jessica Erlenbusch[4], Pol Besenius[4], Leonard Kaps[2,5], Matthias Bros[2], Stephan Grabbe[2], Volker Mailänder[2,3], Jürgen Groll[1], Lutz Nuhn[1*]

1: Institute of Functional Materials and Biofabrication, Center of Polymers for Life, Department of Chemistry and Pharmacy, Julius-Maximilians-Universität Würzburg, 97074 Würzburg, Germany

2: Department of Dermatology, University Medical Center (UMC) of the Johannes Gutenberg-University Mainz, Mainz 55131, Germany

3: Max Planck Institute for Polymer Research, 55128 Mainz, Germany

4: Department of Chemistry, Johannes Gutenberg-University Mainz, 55122 Mainz, Germany

5: Department for Medicine II, University Medical Center Saarland, 66421 Homburg/Saar, Germany

**corresponding author Prof. Dr. Lutz Nuhn*

(lutz.nuhn@uni-wuerzburg.de)

EXPERIMENTAL PROCEDURES

Materials

Unless otherwise stated, commercially available chemicals were obtained from Sigma Aldrich (Taufkirchen, Germany), TCI Chemicals (Tokyo, Japan) or Rapp Polymere (Tübingen, Germany) and used as received. Solvents (HPLC grade) were purchased from Sigma Aldrich or Thermo Fisher Scientific (Darmstadt, Germany). Deuterated solvents were obtained from Deutero (Kastellaun, Germany), Sigma Aldrich or Thermo Fisher Scientific. Fluorescent dyes Cy3-amine and Cy5-amine were obtained from Lumiprobe GmbH (Hannover, Germany) and IRDye800RS-amine was purchased from Li-Cor Biosciences (Lincoln, USA). 1,8-Diazabicyclo[5.4.0]undec-7-ene (DBU) and dichloromethane (DCM) were dried over CaH_2 and distillated before use.

MTC-OEtKBn and MTC-OBn were synthesized as reported in literature.[1,2]

Cell culture medium and supplements as well as Dulbecco's PBS were obtained from Thermo Fisher Scientific. The RAW-Blue^{TM} reporter cell line 264.7 (RRID: CVCL_X594), the RAW-Dual^{TM} reporter cell line 264.7 (RRID: CVCL_A7ZK) and QUANTI-Blue^{TM} substrate was purchased from InvivoGen (San Diego, CA, USA). The macrophage cell liness were cultured in DMEM-GlutaMAX^{TM} medium, which was supplemented with 10% fetal bovine serum, 1% penicillin/streptomycin, 0.02 % normocin, and 0.01% zeocin at 37°C with 5% CO_2 saturation. Contaminations within the cells were excluded due to regular testing.

Materials for *ex vivo* analysis and cell cultures were obtained from Greiner Bio-One GmbH (Frickenhausen, Germany), Sarstedt AG & Co. KG (Nümbrecht, Germany) and BD Biosciences Inc. (Franklin Lakes, NJ, USA). Media supplements and culture media were obtained from Sigma-Aldrich (Taufkirchen, Germany) Carl Roth GmbH (Karlsruhe, Germany), Miltenyi (Bergisch Gladbach, Germany) and Thermo Fisher Scientific (Waltham, MA, USA). Antibodies were obtained either from BioLegend (San Diego, CA, USA) or Thermo Fisher Scientific (Waltham, MA, USA).

Nuclear magnetic resonance spectroscopy

^{1}H and ^{13}C NMR spectra were recorded at room temperature on a Bruker Fourier 300 MHz or Bruker Avance III HD 400 MHz NMR spectrometer (Rheinstetten, Germany). ^{19}F NMR spectra were recorded on a Bruker Avance III HD 400 MHz NMR spectrometer. The chemical shifts (δ) are given in parts per million (ppm) relative to TMS. NMR spectra were evaluated with the software MestReNova 14.0.1-23559 by Mestrelab Research S.L.U. (Santiago de Compostela, Spain). Samples were prepared in respective deuterated solvents and their corresponding signals referenced to residual non-deuterated solvent signals.

Size exclusion chromatography

Gel permeation chromatography was performed either with tetrahydrofuran (THF) or hexafluoro-2-propanol (HFIP) as eluent. In case of THF-GPC, measurements were performed at a flow rate of 1.0 mL/min and a temperature of 40 °C. As column material styrene divinylbenzene (particle size: 10 µm, pore size: 500 Å + 104 Å + 106 Å, obtained from PSS Polymer Standards Service GmbH Mainz, Germany) was used. Detection was performed by IR-detection (Agilent 1260 Infinity RID) and UV-detection (Agilent 1260 Infinity VWD). A polystyrene standard was used for the calibration (Polymer Standard Service GmbH Mainz, Germany). Toluene was used as an internal standard. In case of HFIP-GPC, measurements were performed at a flow rate of 0.8 mL/min at a temperature of 40 °C. As column material modified silica gel (PFG columns, particle size: 7 µm, pore size: 100 Å + 1000 Å, obtained from PSS) was used. Detection was performed by IR-detection (Agilent 1260 Infinity II RID) and UV-detection (Agilent 1260 Infinity II MWD). HFIP with 3 g/L potassium trifluoroacetate was used as an eluent. Molecular weights were determined by using a calibration with PMMA (PSS). The evaluation of the elution diagrams was performed with WinGPC from PSS. Visualization of the data was performed using GraphPad Prism version 5.02.

Mass spectrometry

Matrix Assisted Laser Desorption/Ionization-Time of Flight Mass Spectrometry (MALDI-ToF) measurements were conducted using trans-2-[3-(4-tbutylphenyl)-2-methyl-2-propenylidene]-malononitrile (DCTB) as matrix on a rapifleXTM MALDI-ToF/ToF mass spectrometer from Bruker with a 10 kHz scanning smartbeam 3D laser (Nd:YAG at 355 nm) and a 10-bit 5 GHz digitizer in positive ion reflector mode. Data were evaluated with mMass version 5.5.0 and plotted with GraphPad Prism version 5.02.

Thermogravimetric analysis

Thermogravimetric analysis (TGA) measurements were performed in Al_2O_3-crucibles under argon atmosphere with a heating rate of 10 K min^{-1} between 303 and 1,173 K using a TG 209 F1 Iris® instrument (Netzsch).

Differentila Scanning Calorimetry

Differential scanning calorimetry (DSC) measurements were carried out in Al-crucibles under isobar conditions and nitrogen atmosphere with a heat and cooling rate of 5 K min^{-1} in a DSC 204 F1 Phoenix instrument (Netzsch).

Dynamic Light Scattering

Dynamic light scattering (DLS) measurements were performed on a Zetasizer Nano ZS (Malvern Instruments Ltd, Malvern, U.K.) equipped with a HeNe laser (λ = 633 nm) and detected at a scattering

angle of 173° at 25 °C. Dust was removed from the sample prior to each measurement by filtration through hydrophilic PTFE syringe filter (0.20 µm pore size, Macherey-Nagel). For data analysis, ZetaSizer Software 8.02. was used. Visualization of the data was performed using GraphPad Prism version 5.02.

Electron Microscopy

Transmission electron microscopy (TEM) samples of the block copolymer micelles were prepared in TRIS buffer (0.05 M, pH = 7.4) at a polymer mass concentration of $\beta = 2$ mg/mL. The nanoparticles were recorded by a Tecnai T12 or a Tecnai G2 Spirit instrument from FEI (Hillsboro, USA), both equipped with a LaB_6 cathode operating at 120 kV. The former is equipped with a BioTWIN objective lens whereas the latter makes use of a TWIN lens. Images were collected either using a MegasSYS 1k×1k or a Gatan US1000 2k×2k CCD sensor. 5 µL of the sample were left to absorb to freshly glow discharged copper grids (CF300-Cu, 300 mesh) coated with a 3–4 nm carbon film from Electron Microscopy Sciences (Hatfield, USA) for 1 min and negatively stained afterwards for 20 s with 5 µL of a 2 wt% solution of uranyl acetate. Excess liquid was removed with Whatman® grade 1 filter papers from GE Healthcare Biosciences (Uppsalla, Sweden) after each step.

Ultraviolet-Visible and Fluorescence Spectroscopy

UV-vis spectra were measured using an Agilent Cary 5000 UV-Vis-NIR spectrometer with an ultra-micro quartz glass cuvette (2x2.5 mm aperture, 10 mm pathlength, 50 µL, z height 20 mm). Data evaluation was performed with the Cary WinUV Software for UV-Vis-NIR applications. Visualization of the data was performed using GraphPad Prism version 5.02.

Fluorescence spectroscopy as well as QUANRI-Blue™ and MTT assay absorbance read-out were performed using a Spark 20M Multimode Microplate Reader from Tecan Trading AG (Mannedorf, Switzerland). Visualization of the data was performed using GraphPad Prism version 5.02.

BLOCK COPOLYMER SYNTHESIS

Poly(ethylene glycol)$_{113}$-*b*-poly(MTC-OEtKBn)$_{19}$ (1)

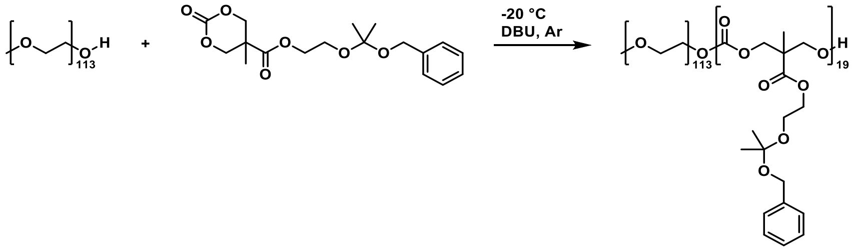

Base-catalyzed ring opening polymerization of the cyclic benzyl ketal carbonate monomer was conducted on a sub-0.5-g scale because the reaction could be performed in Schlenk flasks cooled by a cryostat to -20 °C. Two 10 mL Schlenk flasks were charged with MTC-OEtKBn (211.4 mg, 0.60 mmol, 30 eq) and mPEG$_{113}$-OH (100.0 mg, 0.02 mmol, 1.0 eq), respectively. Both compounds were dried azeotropically with 1 mL benzene by evaporating benzene/water under reduced pressure (instead of benzene, anhydrous toluene can also be applied for removing traces of water azeotropically[3]). To ensure complete removal of water, the drying process was repeated two times. Monomer and initiator were each dissolved in 800 µL dry DCM. The solutions were cooled to -20 °C. Dry DBU (2.99 µL, 0.02 mmol, 1.0 eq) was added to the macroinitiator solution. Subsequently, the monomer solution was added to the macroinitiator solution under argon stream. The reaction was quenched at a monomer conversion of 80% by precipitating the block copolymer in diethyl ether at -20 °C. The precipitated polymer was isolated by centrifugation (4000 rpm, 0 °C, 20 min). In order to purify the block copolymer, it was re-dissolved in DCM, precipitated in diethyl ether and isolated by centrifugation two more times. Subsequently, the isolated product was freeze dried from benzene (instead of benzene, anhydrous toluene can also be applied for removing traces of water azeotropically[3]), yielding a white voluminous powder (216.6 mg, 80%).

^{1}H NMR (300 MHz, DMSO-d$_6$) δ [ppm]: 7.29–7.22 (m, 5*n*H, Ar-*H*); 4.44–4.41 (m, 2*n*H, Ar-C*H*$_2$-); 4.20–4.16 (m, 6*n*H, -O-(C=O)-O-C*H*$_2$-C-C*H*$_2$-, Ar-CH$_2$-O-C-O-CH$_2$-C*H*$_2$-); 3.57 (m, 2*n*H, Ar-CH$_2$-O-C-O-C*H*$_2$-); 3.51 (m, 452H mPEG-*H*); 3.24 (s, 3H, C*H*$_3$-PEG); 1.33–1.31 (m, 6*n*H, Ar-CH$_2$-O-(C-(C*H*$_3$)$_2$)-); 1.08–1.03 (m, 3*n*H, mPEG$_{113}$-O-(C=O)-O-CH$_2$-C-C*H*$_3$).

GPC^{UV} (THF, PS calibration): M_n = 12970 g/mol, M_w = 13470 g/mol, $Đ$ = 1.04.

GPC^{RI} (THF, PS calibration): M_n = 12180 g/mol, M_w = 12800 g/mol, $Đ$ = 1.05.

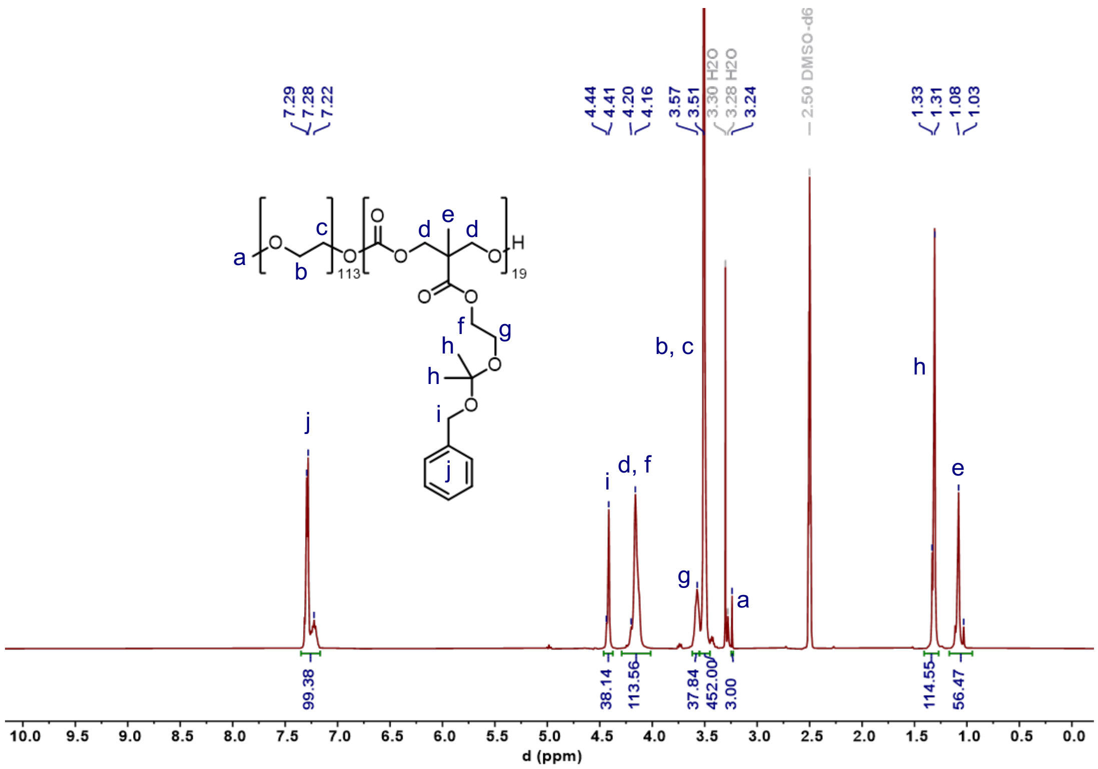

Figure S1: ^{1}H NMR spectrum (300 MHz, DMSO-d_6) of poly(ethylene glycol)$_{113}$-*b*-poly(MTC-OEtKBn)$_{19}$ (**1**). The degree of polymerization DP = 19 was obtained by comparing the carbonate block signals to mPEG's methoxy end group (a) as well as 5kDa mPEG's 113 repetition units (b,c).

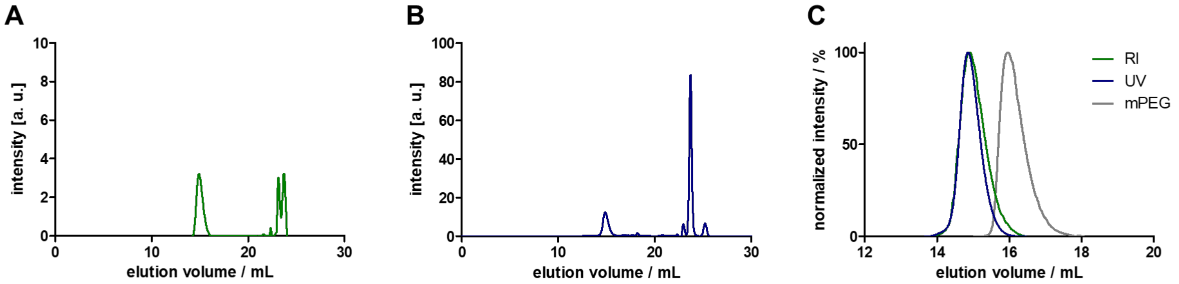

Figure S2: THF-GPC analysis of poly(ethylene glycol)$_{113}$-*b*-poly(MTC-OEtKBn)$_{19}$ (**1**). **A**: RI-traces and **B**: UV-traces of poly(ethylene glycol)$_{113}$-*b*-poly(MTC-OEtKBn)$_{19}$ (**1**) **C**: Section of the overlayed normalized UV- (blue) and RI-signal (green) of the polymer peak next to the mPEG$_{113}$-OH RI-signal (grey).

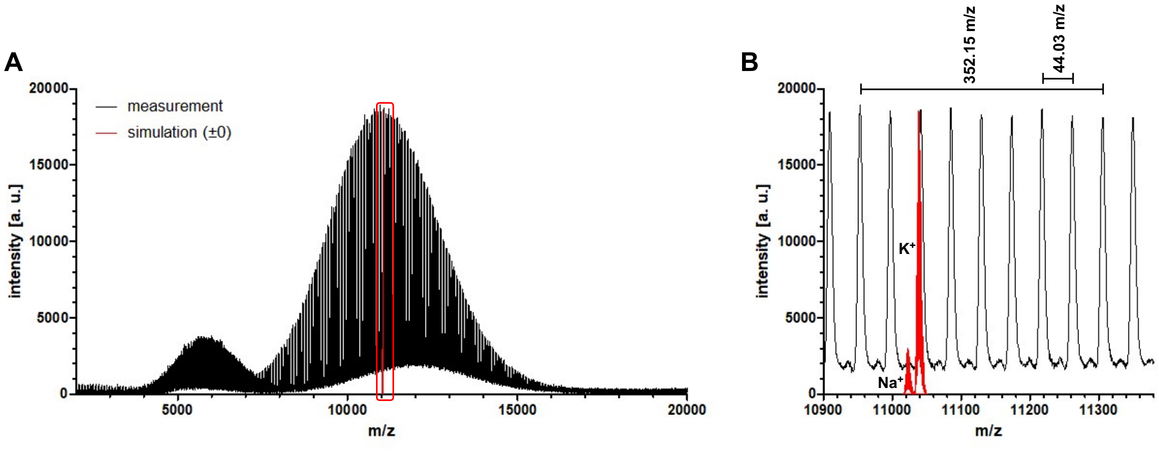

Figure S3: MALDI-ToF mass spectrum of poly(ethylene glycol)$_k$-*b*-poly(MTC-OEtKBn)$_{19}$ (**1**). **A**: Full mass spectrum. The red box labels the magnified area in **B**. The simulation (red) refers to the species with k = 113 and n = 17, cationized with Na^+ or K^+. The assigned peak distances label the mass differences corresponding to the repeating units ethylene glycol (44.03 m/z) and MTC-OEtKBn (352.15 m/z).

Poly(ethylene glycol)$_{113}$-*b*-poly(MTC-OBn)$_{21}$ (2)

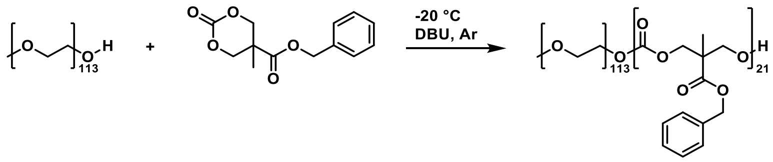

Base-catalyzed ring opening polymerization of the cyclic benzyl ketal carbonate monomer was conducted on a sub-0.5-g scale because the reaction could be performed in Schlenk flasks cooled by a cryostat to -20 ºC. Two 10 mL Schlenk flasks were charged with MTC-OBn (625.6 mg, 2.5 mmol, 25 eq) and mPEG$_{113}$-OH as macroinitiator (500.0 mg, 0.10 mmol, 1.0 eq), respectively. Both compounds were dried azeotropically with 1 mL benzene by evaporating benzene/water under reduced pressure (instead of benzene, anhydrous toluene can also be applied for removing traces of water azeotropically[3]). To ensure complete removal of water, the drying process was repeated two times. Monomer and initiator were each dissolved in 4.0 mL dry DCM. The solutions were cooled to -20 °C. Dry DBU (14.9 µL, 0.10 mmol, 1.0 eq) was added to the macroinitiator solution. Subsequently, the monomer solution was added to the macroinitiator solution under argon stream. The reaction was quenched at a monomer conversion of 94% by precipitating the block copolymer in diethyl ether at -20 °C. The precipitated polymer was isolated by centrifugation (4000 rpm, 0 °C, 20 min). In order to purify the block copolymer, it was re-dissolved in DCM, precipitated in diethyl ether and isolated by centrifugation two more times. Subsequently, the isolated product was freeze dried from benzene (instead of benzene, anhydrous toluene can also be applied for removing traces of water azeotropically[3]), yielding a white voluminous powder (892.0 mg, 82%).

^{1}H NMR (300 MHz, CDCl$_3$) δ [ppm]: 7.32–7.29 (m, 5*m*H, Ar-*H*); 5.16–5.12 (m, 2*m*H, Ar-C*H$_2$*-); 4.31–4.23 (m, 4*m*H, -O-(C=O)-O-C*H$_2$*-C-C*H$_2$*-); 3.64 (m, 452H mPEG-*H*); 3.38 (s, 3H, C*H$_3$*-PEG); 1.25–1.19 (m, 3*m*H, -O-(C=O)-O-CH$_2$-C-C*H$_3$*).

GPC^{UV} (THF, PS calibration): M_n = 11680 g/mol, M_w = 12150 g/mol, $Đ$ = 1.04.

GPC^{RI} (THF, PS calibration): M_n = 10910 g/mol, M_w = 11550 g/mol, $Đ$ = 1.06.

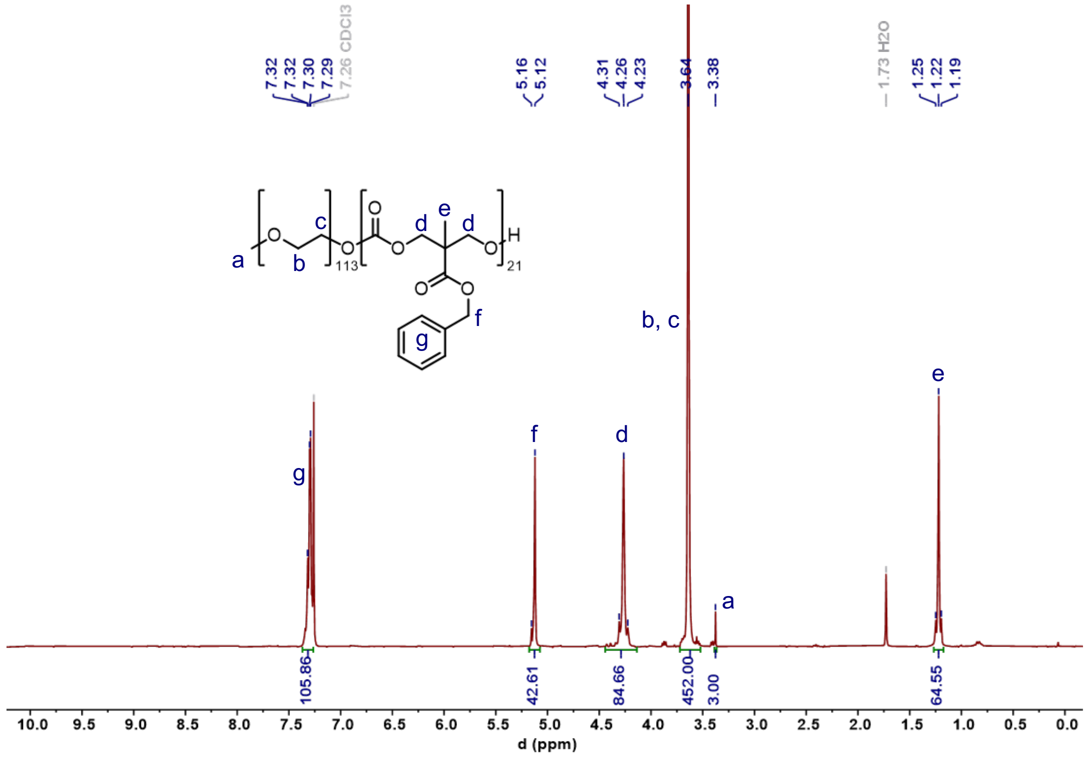

Figure S4: ^{1}H NMR spectrum (300 MHz, $CDCl_3$) of poly(ethylene glycol)$_{113}$-*b*-poly(MTC-OBn)$_{21}$ (**2**). The degree of polymerization DP = 21 was obtained by comparing the carbonate block signals to mPEG's methoxy end group (a) as well as 5kDa mPEG's 113 repetition units (b,c).

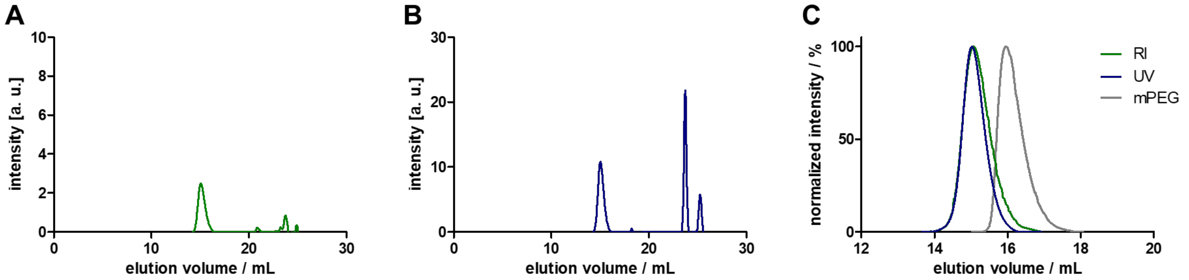

Figure S5: THF-GPC analysis of poly(ethylene glycol)$_{113}$-*b*-poly(MTC-OBn)$_{21}$ (**2**). **A**: RI-traces and **B**: UV-traces of poly(ethylene glycol)$_{113}$-*b*-poly(MTC-OBn)$_{21}$ (**2**) **C**: Section of the overlayed normalized UV- (blue) and RI-signal (green) of the polymer peak next to the mPEG$_{113}$-OH RI-signal (grey).

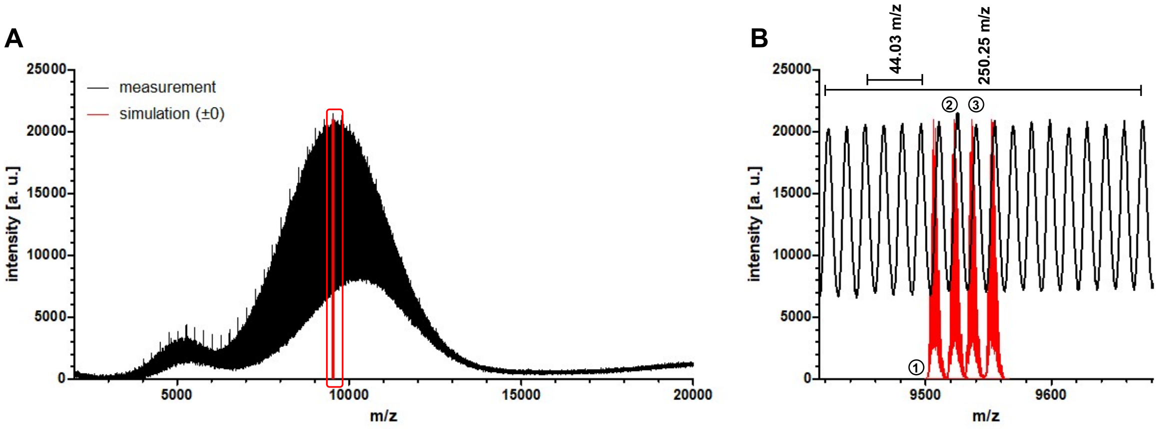

Figure S6: MALDI-ToF mass spectrum of poly(ethylene glycol)$_k$-*b*-poly(MTC-OBn)$_{21}$ (**2**). **A**: Full mass spectrum. The red box labels the magnified area in **B**. The simulation (red) refers to species with different block compositions (1: $k = 118$, $m = 17$, Na^+, 2: K^+, 3: $k = 113$, $m = 18$, Na^+, 4: K^+). The assigned peak distances label the mass differences corresponding to the repeating unit ethylene glycol (44.03 m/z) and MTC-OBn (250.25 m/z).

THERMOMETRIC CHARACTERIZATION

Thermogravimetric Analysis (TGA) of Poly(ethylene glycol)$_{113}$-*b*-poly(MTC-OEtKBn)$_{19}$

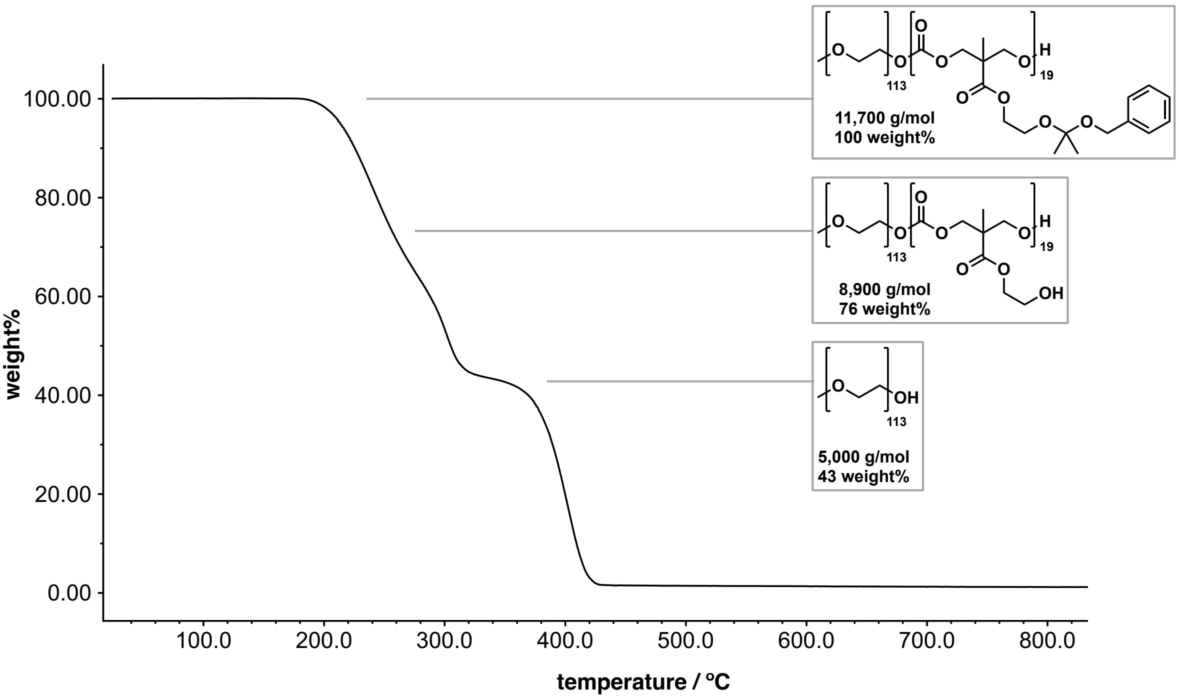

Figure S7: Results of the thermogravimetric analysis (TGA) of poly(ethylene glycol)$_{113}$-*b*-poly(MTC-OEtKBn)$_{19}$. The block copolymer is stable until 200 °C, where the benzyl ketal functionalities start the decompose thermally. At around 260-300 °C the carbonate block starts to decompose, while at around 380- 420 °C the remaining PEG block decomposes thermally, too.

Differential Scanning Calorimetry (DSC) of Poly(ethylene glycol)$_{113}$, Poly(MTC-OEtKBn)$_{19}$ and Poly(ethylene glycol)$_{113}$-*b*-poly(MTC-OEtKBn)$_{19}$

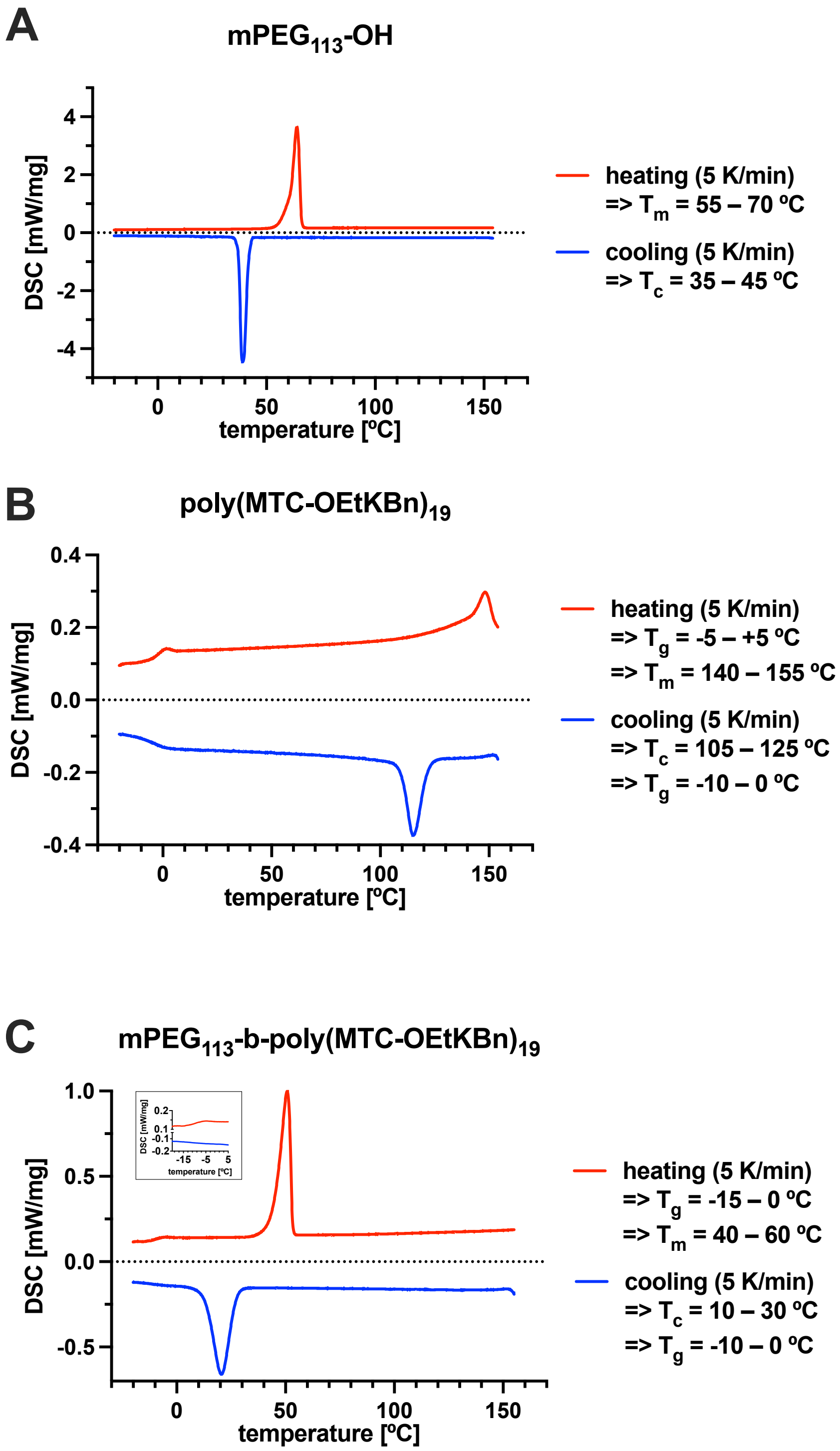

Figure S8: Results of the differential scanning calorimetry (DSC) analyses of the homopolymers poly(ethylene glycol)$_{113}$ and poly(MTC-OEtKBn)$_{19}$, as well as the block copolymer poly(ethylene glycol)$_{113}$-*b*-poly(MTC-OEtKBn)$_{19}$. **A)** Poly(ethylene glycol)$_{113}$ melts around 55-70 °C during heating and starts to crystallize around 35-45 °C during cooling. **B)** Poly(MTC-OEtKBn)$_{19}$ is has a glass transition around -5 to +5 °C and a melting around 140-150 °C during heating. During cooling the crystallization is around 105-125 °C and the glass transition around -10 to 0ºC. **C)** For the block copolymer poly(ethylene glycol)$_{113}$-*b*-poly(MTC-OEtKBn)$_{19}$ both the glass transition of the poly(MTC-OEtKBn)$_{19}$ block and the melting/crystallization of the poly(ethylene glycol) block have decreased. During heating a glass transition is found around -15 to 0 °C and a melting around 40-60 °C, while during cooling a crystallization appears around 10-30 °C and the glass transition appears around -10 to 0ºC.

END GROUP FUNCTIONALIZATION

Introduction of Active PFP-Carbonate Chain Ends

Poly(ethylene glycol)$_{113}$-*b*-poly(MTC-OEtKBn)$_{19}$-(C=O)-O-PFP

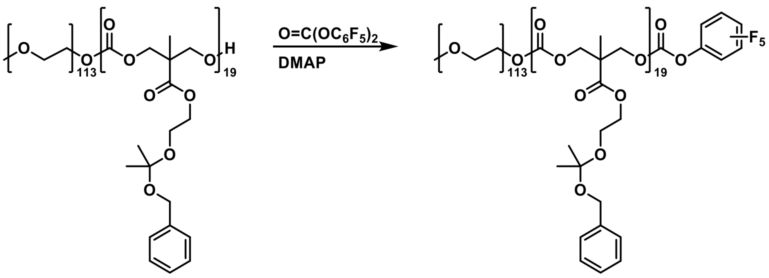

A 10 mL Schlenk flask was equipped with a stirring bar and poly(ethylene glycol)$_{113}$-*b*-poly(MTC-OEtKBn)$_{19}$ (**1**) (60.0 mg, 5.13 µmol, 1.0 eq). The polymer was dried by azeotrope distillation with 0.5 mL benzene three times (instead of benzene, anhydrous toluene can also be applied for removing traces of water azeotropically[3]). After dissolving the polymer in 1.0 mL dry THF, bis(pentafluorophenyl) carbonate (10.1 mg, 25.6 µmol, 5.0 eq) and 4-dimethylaminopyridine (DMAP, 0.626 mg, 5.13 µmol, 1.0 eq) were added. The reaction mixture was stirred at room temperature for 16 h. Afterwards it was precipitated in ice-cold diethyl ether. The precipitated polymer was isolated by centrifugation (4000 rpm, 0 °C, 20 min). In order to purify the block copolymer, it was re-dissolved in DCM, precipitated in diethyl ether and isolated by centrifugation two more times. Subsequently, the isolated product was freeze dried from benzene (instead of benzene, anhydrous toluene can also be applied for removing traces of water azeotropically[3]), yielding poly(ethylene glycol)$_{113}$-*b*-poly(MTC-OEtKBn)$_{19}$-(C=O)-O-PFP as a white voluminous solid (49.9 mg, 83%).

^{1}H NMR (400 MHz, CD_2Cl_2) δ [ppm]: 7.32–7.31 (m, 5*n*H, Ar-*H*); 4.49–4.48 (m, 2*n*H, Ar-C*H$_2$*-); 4.24–4.21 (m, 6*n*H, -O-(C=O)-O-C*H$_2$*-C-C*H$_2$*-, Ar-CH$_2$-O-C-O-CH$_2$-C*H$_2$*-); 3.66–3.64 (m, 2*n*H, Ar-CH$_2$-O-C-O-C*H$_2$*-); 3.60 (m, 452H mPEG-*H*); 3.34 (s, 3H, C*H$_3$*-PEG); 1.41–1.40 (m, 6*n*H, Ar-CH$_2$-O-(C-(C*H$_3$*)$_2$)-); 1.25–1.19 (m, 3*n*H, mPEG$_{113}$-O-(C=O)-O-CH$_2$-C-C*H$_3$*).

^{19}F NMR (376 MHz, CD_2Cl_2) δ [ppm]: -153.34 (d, 2F, *ortho*), -158.10 (t, 1F, *para*), -162.64 (dd, 2F, *meta*).

GPC^{UV} (THF, PS calibration): M_n = 13130 g/mol, M_w = 13630 g/mol, $Đ$ = 1.04.

GPC^{RI} (THF, PS calibration): M_n = 12560 g/mol, M_w = 13170 g/mol, $Đ$ = 1.05.

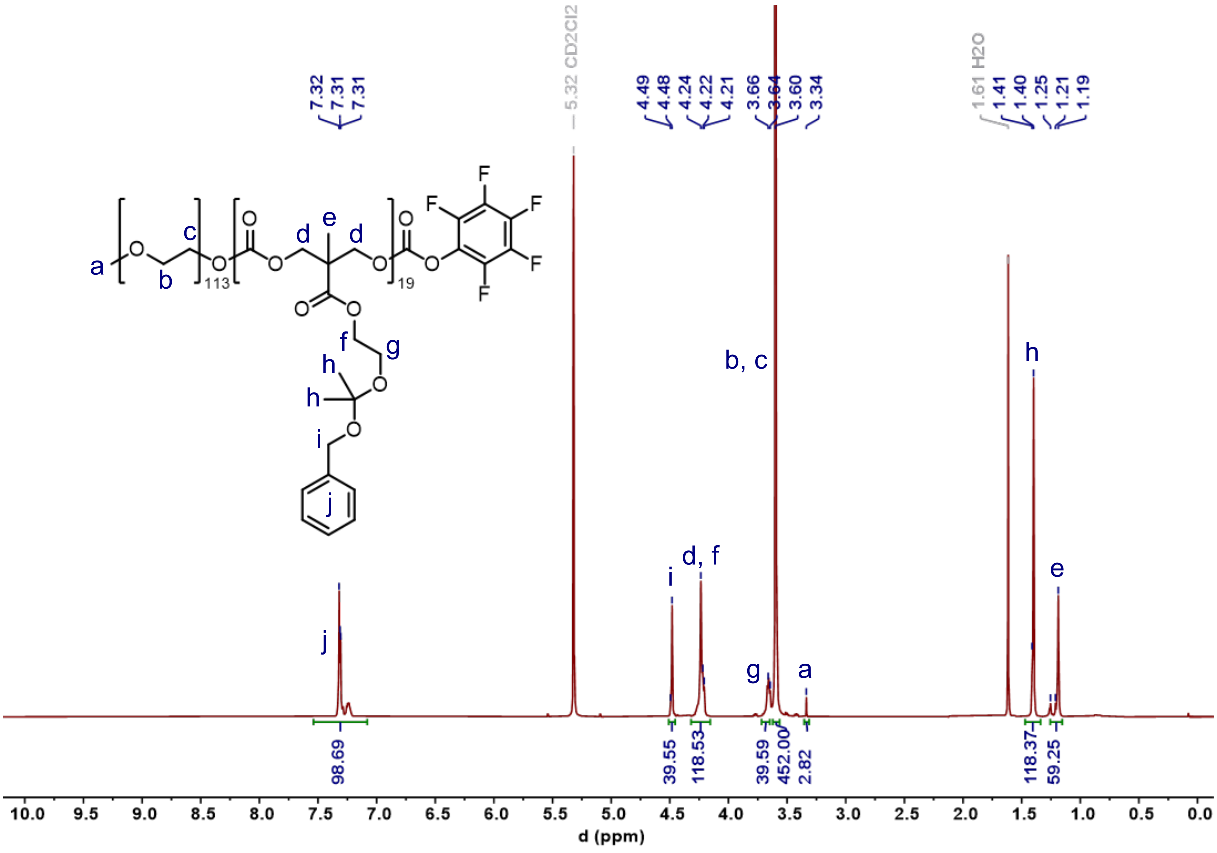

Figure S9: ^{1}H NMR spectrum (400 MHz, CD_2Cl_2) of poly(ethylene glycol)$_{113}$-*b*-poly(MTC-OEtKBn)$_{19}$-(C=O)-O-PFP.

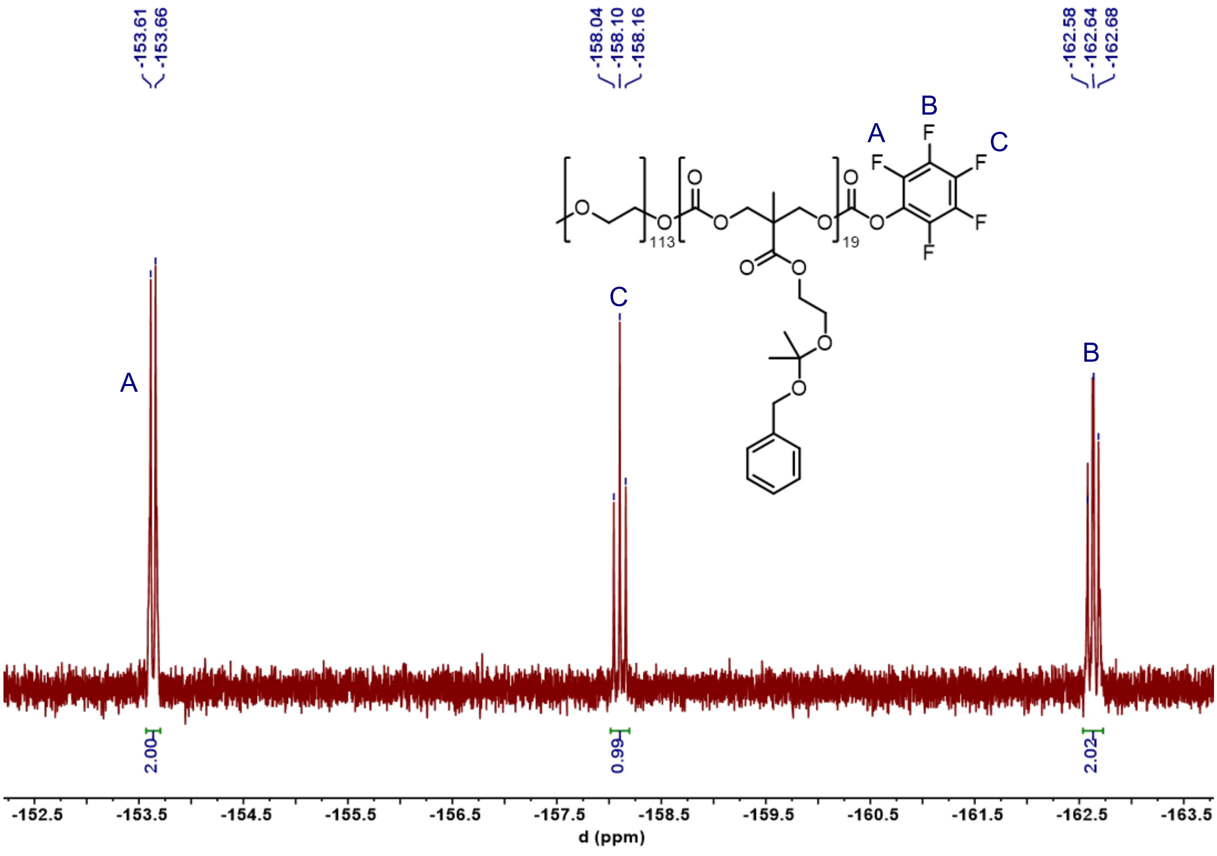

Figure S10: ^{19}F NMR spectrum (376 MHz, CD_2Cl_2) of poly(ethylene glycol)$_{113}$-*b*-poly(MTC-OEtKBn)$_{19}$-(C=O)-O-PFP.

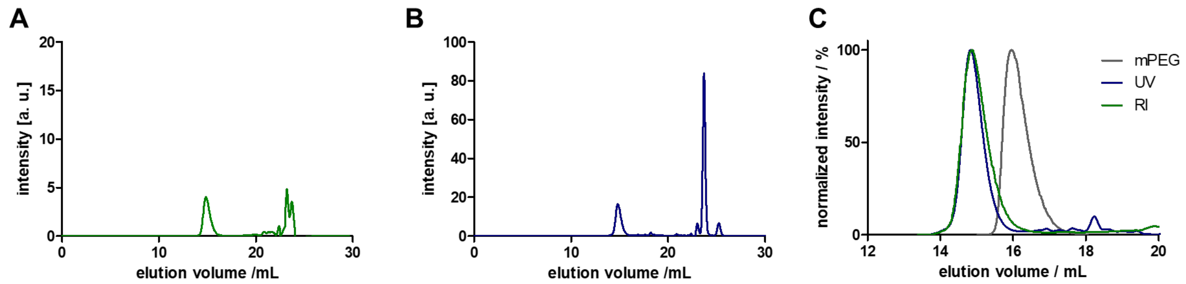

Figure S11: THF-GPC analysis of poly(ethylene glycol)$_{113}$-*b*-poly(MTC-OEtKBn)$_{19}$-(C=O)-O-PFP. **A**: RI-traces and **B**: UV-traces of poly(ethylene glycol)$_{113}$-*b*-poly(MTC-OEtKBn)$_{19}$-(C=O)-O-PFP. **C**: Section of the overlayed normalized UV- (blue) and RI-signal (green) of the polymer peak next to the mPEG$_{113}$-OH RI-signal (grey).

Poly(ethylene glycol)$_{113}$-*b*-poly(MTC-OBn)$_{21}$-(C=O)-O-PFP

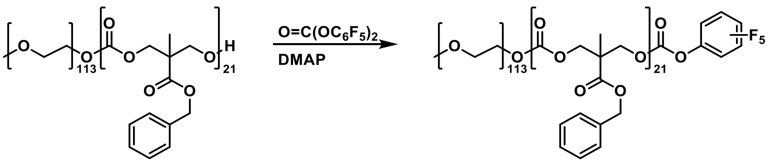

A 10 mL Schlenk flask was equipped with a stirring bar and poly(ethylene glycol)$_{113}$-*b*-poly(MTC-OBn)$_{21}$ (**2**) (60.0 mg, 5.70 µmol, 1.0 eq). The polymer was dried by azeotrope distillation with 0.5 mL benzene three times (instead of benzene, anhydrous toluene can also be applied for removing traces of water azeotropically[3]). After dissolving the polymer in 1.0 mL dry THF, bis(pentafluorophenyl) carbonate (11.2 mg, 28.5 µmol, 5.0 eq) and 4-dimethylaminopyridine (DMAP, 0.697 mg, 5.70 µmol, 1.0 eq) were added. The reaction mixture was stirred at room temperature for 16 h. Afterwards it was precipitated in ice-cold diethyl ether. The precipitated polymer was isolated by centrifugation (4000 rpm, 0 °C, 20 min). In order to purify the block copolymer, it was re-dissolved in DCM, precipitated in diethyl ether and isolated by centrifugation two more times. Subsequently, the isolated product was freeze dried from benzene (instead of benzene, anhydrous toluene can also be applied for removing traces of water azeotropically[3]), yielding poly(ethylene glycol)$_{113}$-*b*-poly(MTC-OBn)$_{21}$-(C=O)-O-PFP as a white voluminous solid (57.6 mg, 96%).

^{1}H NMR (400 MHz, CD_2Cl_2) δ [ppm]: 7.35–7.27 (m, 5*m*H, Ar-*H*); 5.17–5.12 (m, 2*m*H, Ar-CH_2-); 4.29–4.20 (m, 4*m*H, -O-(C=O)-O-CH_2-C-CH_2-); 3.60 (m, 452H mPEG-*H*); 3.34 (s, 3H, CH_3-PEG); 1.25–1.22 (m, 3*m*H, mPEG$_{113}$-O-(C=O)-O-CH$_2$-C-CH_3).

^{19}F NMR (376 MHz, CD_2Cl_2) δ [ppm]:-153.62 (d, 2F, *ortho*), -158.08 (t, 1F, *para*), -162.63 (dd, 2F, *meta*).

GPC^{UV} (THF, PS calibration): M_n = 11930 g/mol, M_w = 12410 g/mol, $Đ$ = 1.04.

GPC^{RI} (THF, PS calibration): M_n = 11200 g/mol, M_w = 11810 g/mol, $Đ$ = 1.05.

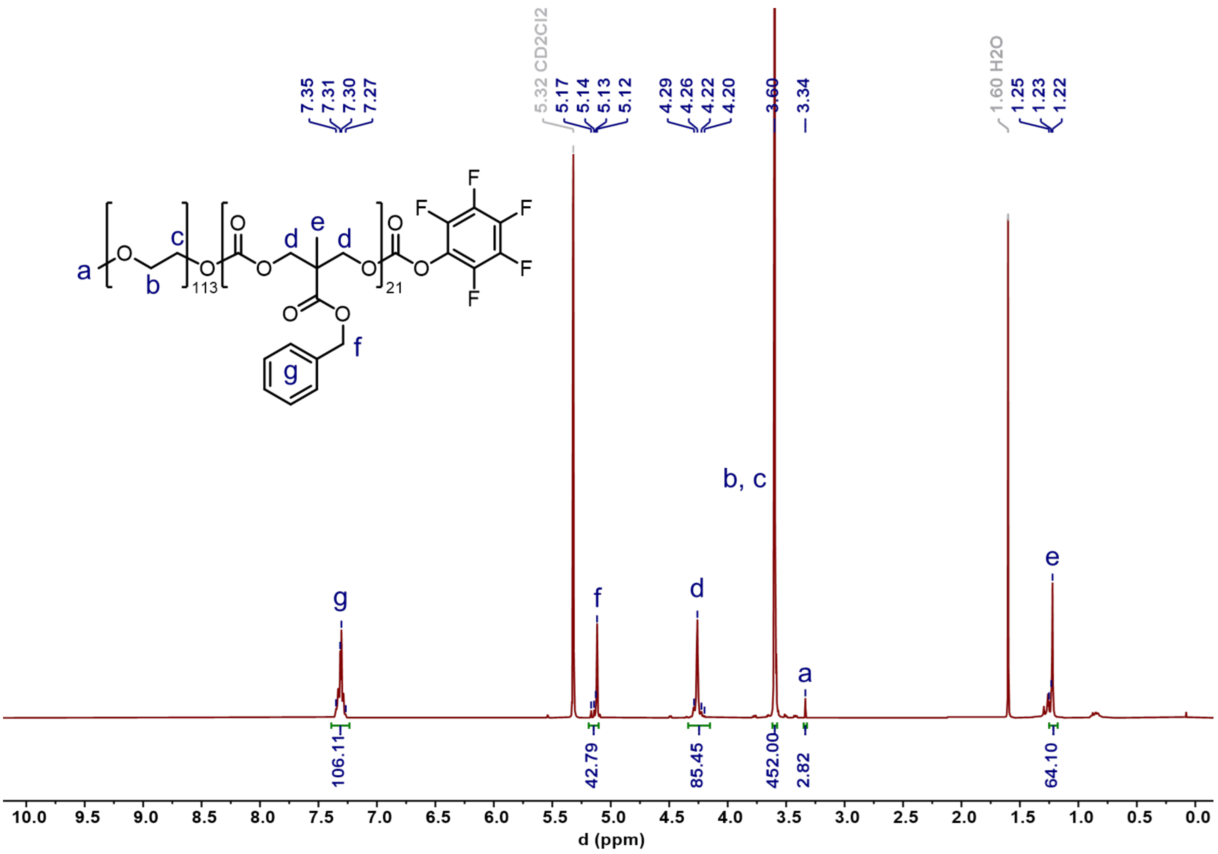

Figure S12: ^{1}H NMR spectrum (400 MHz, CD_2Cl_2) of poly(ethylene glycol)$_{113}$-*b*-poly(MTC-OBn)$_{21}$-(C=O)-O-PFP.

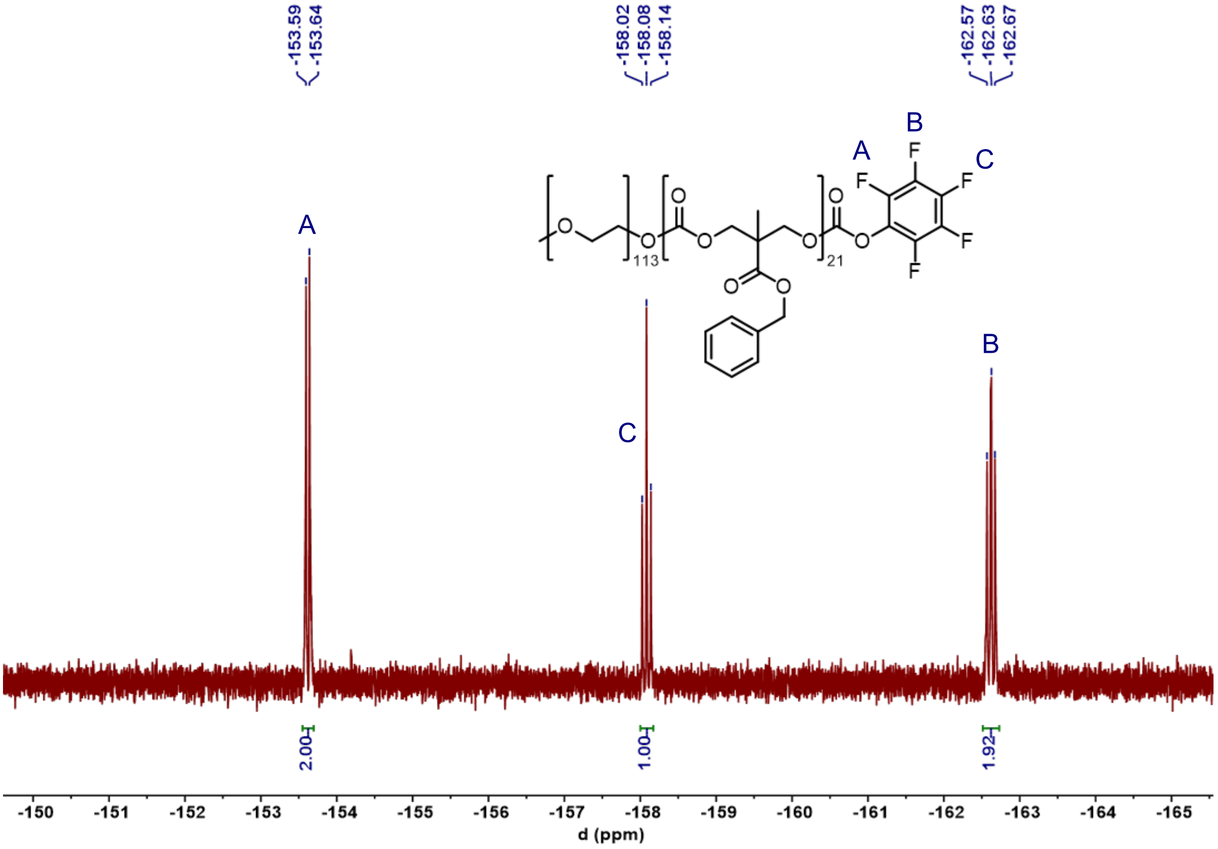

Figure S13: ^{19}F NMR spectrum (376 MHz, CD2Cl2) of poly(ethylene glycol)$_{113}$-*b*-poly(MTC-OBn)$_{21}$-(C=O)-O-PFP.

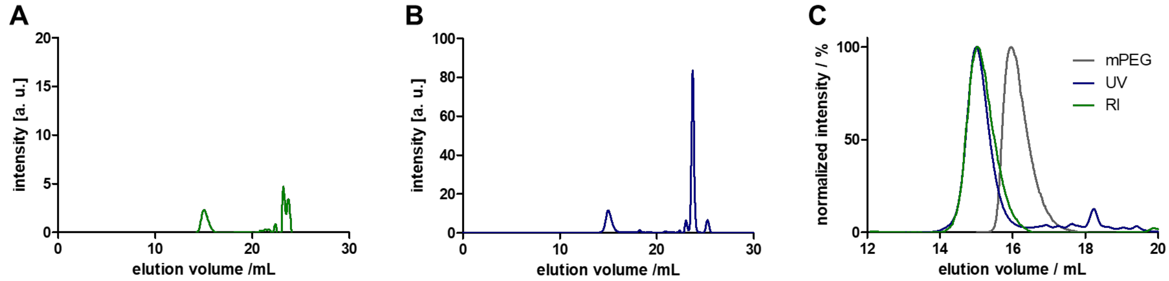

Figure S14: THF-GPC analysis of poly(ethylene glycol)$_{113}$-*b*-poly(MTC-OBn)$_{21}$-(C=O)-O-PFP. **A**: RI-traces and **B**: UV-traces of poly(ethylene glycol)$_{113}$-*b*-poly(MTC-OBn)$_{21}$-(C=O)-O-PFP. **C**: Section of the overlayed normalized UV- (blue) and RI-signal (green) of the polymer peak next to the mPEG$_{113}$-OH RI-signal (grey).

Drug Conjugation

Poly(ethylene glycol)$_{113}$-*b*-poly(MTC-OEtKBn)$_{19}$-(C=O)-NH-IMDQ

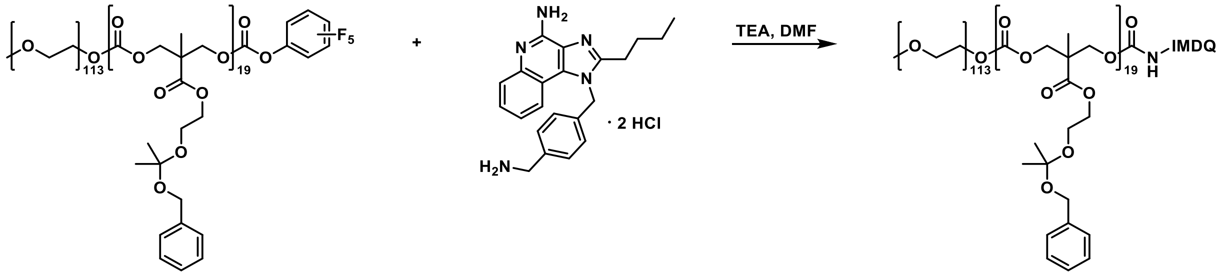

A 10 mL Schlenk flask was equipped with a stirring bar and poly(ethylene glycol)$_{113}$-*b*-poly(MTC-OEtKBn)$_{19}$-(C=O)-O-PFP (6.5 mg, 0.55 µmol, 1.0 eq). The polymer was dried by azeotrope distillation with 0.5 mL benzene three times (instead of benzene, anhydrous toluene can also be applied for removing traces of water azeotropically[3]). After dissolving the polymer in 500 µL dry DMF, 21.6 µL of a 10 mg/mL DMSO stock solution IMDQ·HCl (0.22 mg, 25.6 µmol, 1.0 eq) and triethyl amine (0.11 µL, 0.82 µmol, 1.5 eq) were added. The reaction mixture was stirred at room temperature for 16 h. Afterwards, it was precipitated in ice-cold diethyl ether. The precipitated polymer was isolated by centrifugation (4000 rpm, 0 °C, 20 min). In order to purify the block copolymer, it was re-dissolved in DCM, precipitated in diethyl ether and isolated by centrifugation two more times. Subsequently, the isolated product freeze dried from benzene (instead of benzene, anhydrous toluene can also be applied for removing traces of water azeotropically[3]), yielding poly(ethylene glycol)$_{113}$-*b*-poly(MTC-OEtKBn)$_{19}$-(C=O)-NH-IMDQ as a white voluminous solid (6.4 mg, 98%).

GPC^{UV} (THF, PS calibration): M_n = 6244 g/mol, M_w = 6818 g/mol, $Đ$ = 1.09.

GPC^{RI} (THF, PS calibration): M_n = 6246 g/mol, M_w = 6875 g/mol, $Đ$ = 1.10.

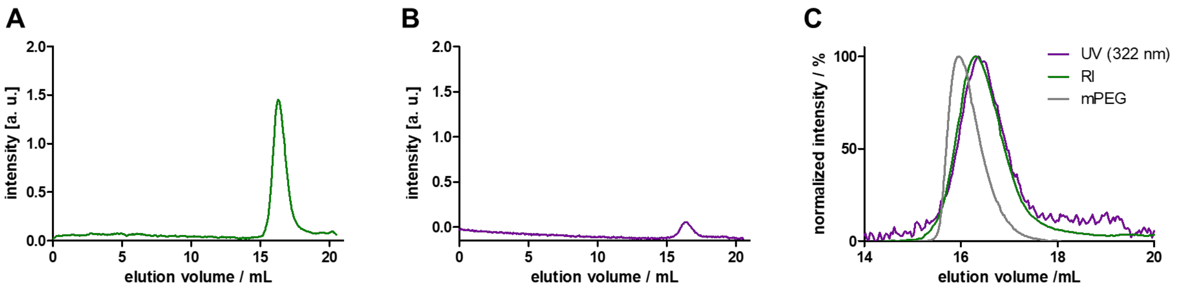

Figure S15: THF-GPC analysis of poly(ethylene glycol)$_{113}$-*b*-poly(MTC-OEtKBn)$_8$-(C=O)-NH-IMDQ. **A**: RI-traces and **B**: UV-traces (322 nm) of poly(ethylene glycol)$_{113}$-*b*-poly(MTC-OEtKBn)$_8$-(C=O)-NH-IMDQ. **C**: Section of the overlayed normalized UV- (322 nm, purple) and RI-signal (green) of the polymer peak next to the mPEG$_{113}$-OH RI-signal (grey).

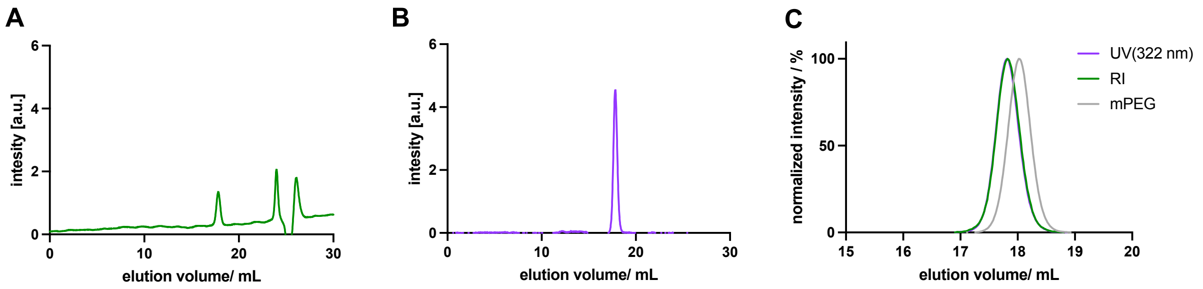

Figure S16: HFIP-GPC analysis of poly(ethylene glycol)$_{113}$-*b*-poly(MTC-OEtKBn)$_{19}$-(C=O)-NH-IMDQ. **A**: RI-traces and **B**: UV-traces (322 nm) of poly(ethylene glycol)$_{113}$-*b*-poly(MTC-OEtKBn)$_{19}$-(C=O)-NH-IMDQ. **C**: Section of the overlayed normalized UV- (322 nm, purple) and RI-signal (green) of the polymer peak next to the mPEG$_{113}$-OH RI-signal (grey).

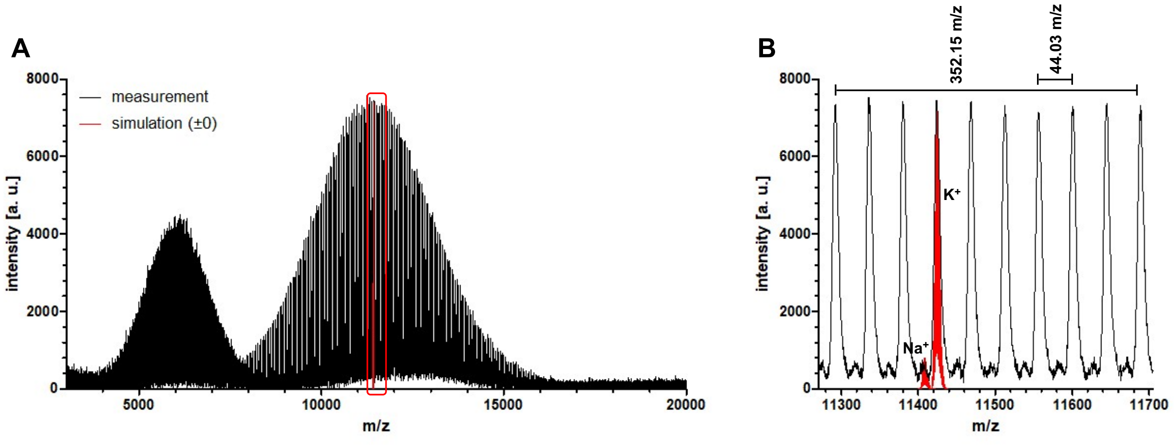

Figure S17: MALDI-ToF mass spectrum of poly(ethylene glycol)$_{113}$-*b*-poly(MTC-OEtKBn)$_{19}$-(C=O)-NH-IMDQ. **A**: Full mass spectrum. The red box labels the magnified area in **B**. The simulation (red) refers to the species with $k = 113$ and $n = 17$, cationized with Na^+ or K^+. The assigned peak distances label the mass differences corresponding to the repeating units ethylene glycol (44.03 m/z) and MTC-OEtKBn (352.15 m/z).

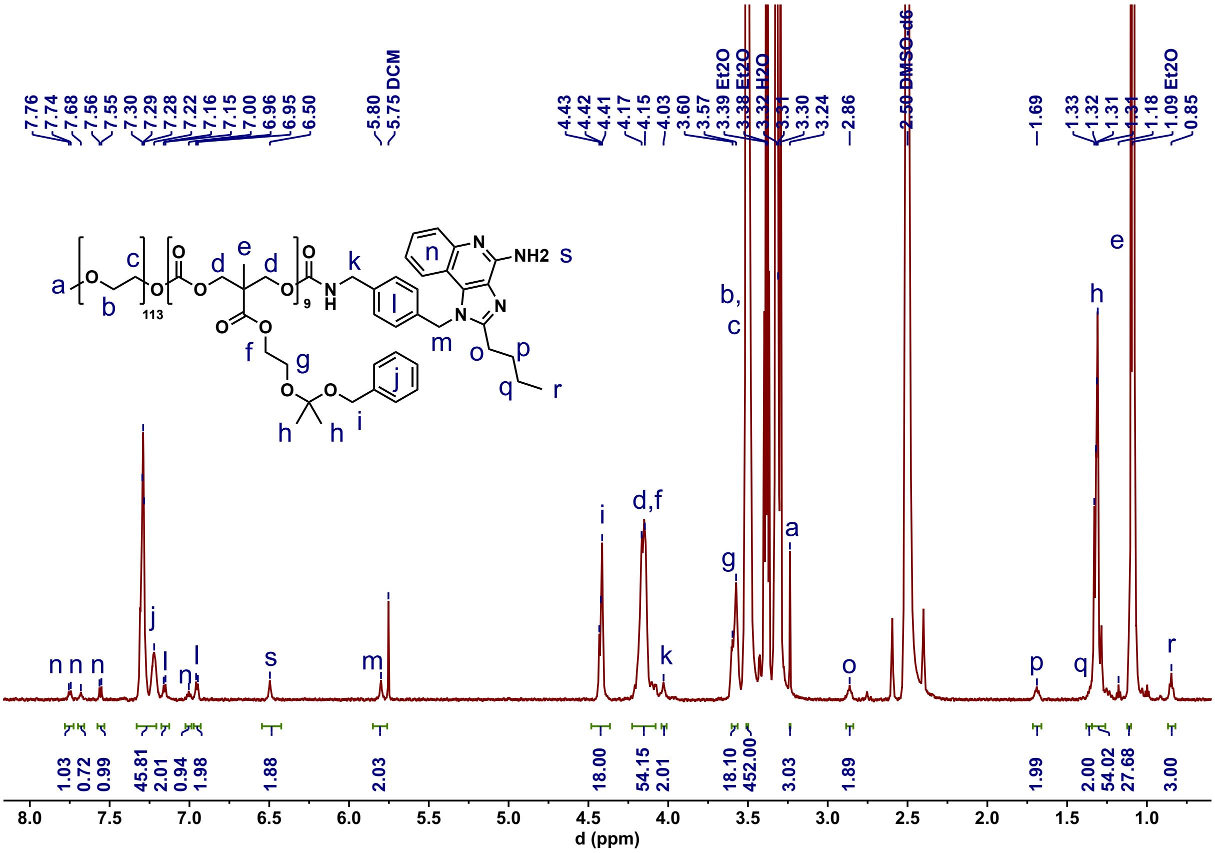

Figure S18: ^{1}H NMR spectrum (400 MHz, DMSO-d_6) of poly(ethylene glycol)$_{113}$-*b*-poly(MTC- OEtKBn)$_9$-(C=O)-NH-IMDQ.

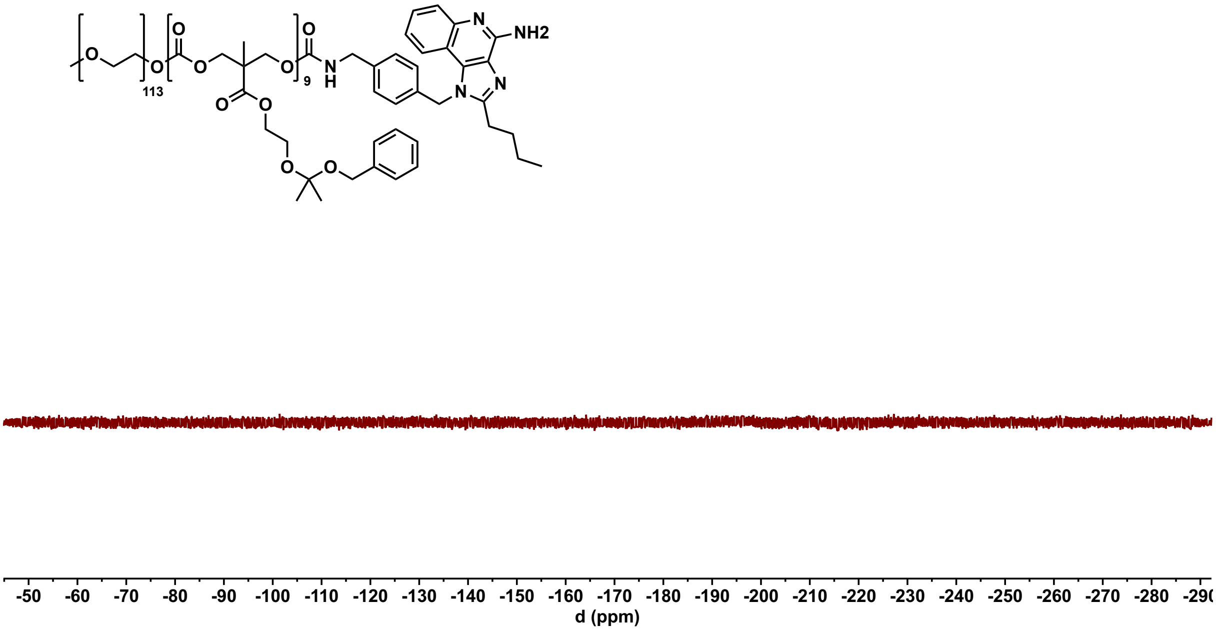

Figure S19: ^{19}F NMR spectrum (376 MHz, DMSO-d_6) of poly(ethylene glycol)$_{113}$-*b*-poly(MTC-OEtKBn)$_9$-(C=O)-NH-IMDQ.

Poly(ethylene glycol)$_{113}$-*b*-poly(MTC-OBn)$_{21}$-(C=O)-NH-IMDQ

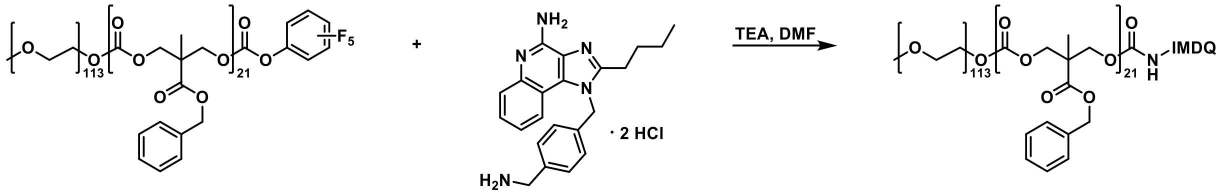

A 10 mL Schlenk flask was equipped with a stirring bar and poly(ethylene glycol)$_{113}$-*b*-poly(MTC-OBn)$_{21}$-(C=O)-O-PFP (10.0 mg, 0.93 µmol, 1.0 eq). The polymer was dried by azeotrope distillation with 0.5 mL benzene three times (instead of benzene, anhydrous toluene can also be applied for removing traces of water azeotropically[3]). After dissolving the polymer in 500 µL dry DMF, 36.9 µL of a 10 mg/mL DMSO stock solution IMDQ·HCl (0.37 mg, 0.93 µmol, 1.0 eq) and triethyl amine (0.19 µL, 1.4 µmol, 1.5 eq) were added. The reaction mixture was stirred at room temperature for 16 h. Afterwards, it was precipitated in ice-cold diethyl ether. The precipitated polymer was isolated by centrifugation (4000 rpm, 0 °C, 20 min). In order to purify the block copolymer, it was re-dissolved in DCM, precipitated in diethyl ether and isolated by centrifugation two more times. Subsequently, the isolated product freeze dried from benzene (instead of benzene, anhydrous toluene can also be applied for removing traces of water azeotropically[3]), yielding poly(ethylene glycol)$_{113}$-*b*-poly(MTC-OBn)$_{21}$-(C=O)-NH-IMDQ as a white voluminous solid (10.0 mg, 100%).

GPC^{UV} (THF, PS calibration): M_n = 4866 g/mol, M_w = 5233 g/mol, $Đ$ = 1.08.

GPC^{RI} (THF, PS calibration): M_n = 4869 g/mol, M_w = 5266 g/mol, $Đ$ = 1.08.

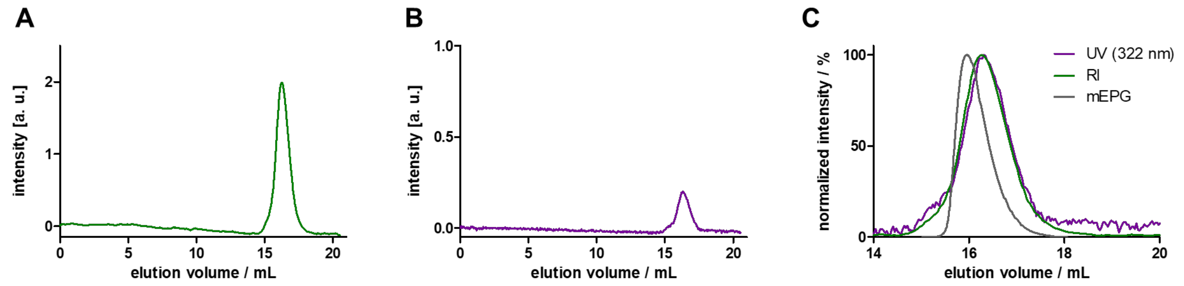

Figure S20: THF-GPC analysis of poly(ethylene glycol)$_{113}$-*b*-poly(MTC-OBn)$_{13}$-(C=O)-NH-IMDQ. **A**: RI-traces and **B**: UV-traces (322 nm) of poly(ethylene glycol)$_{113}$-*b*-poly(MTC-OBn)$_{13}$-(C=O)-NH-IMDQ. **C**: Section of the overlayed normalized UV- (322 nm, purple) and RI-signal (green) of the polymer peak next to the mPEG$_{113}$-OH RI-signal (grey).

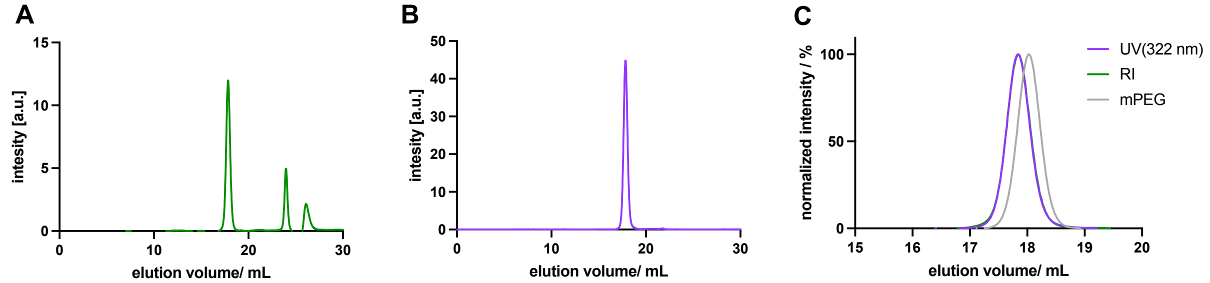

Figure S21: HFIP-GPC analysis of poly(ethylene glycol)$_{113}$-*b*-poly(MTC-OBn)$_{21}$-(C=O)-NH-IMDQ. **A**: RI-traces and **B**: UV-traces (322 nm) of poly(ethylene glycol)$_{113}$-*b*-poly(MTC-OBn)$_{21}$-(C=O)-NH-IMDQ. **C**: Section of the overlayed normalized UV- (322 nm, purple) and RI-signal (green) of the polymer peak next to the mPEG$_{113}$-OH RI-signal (grey).

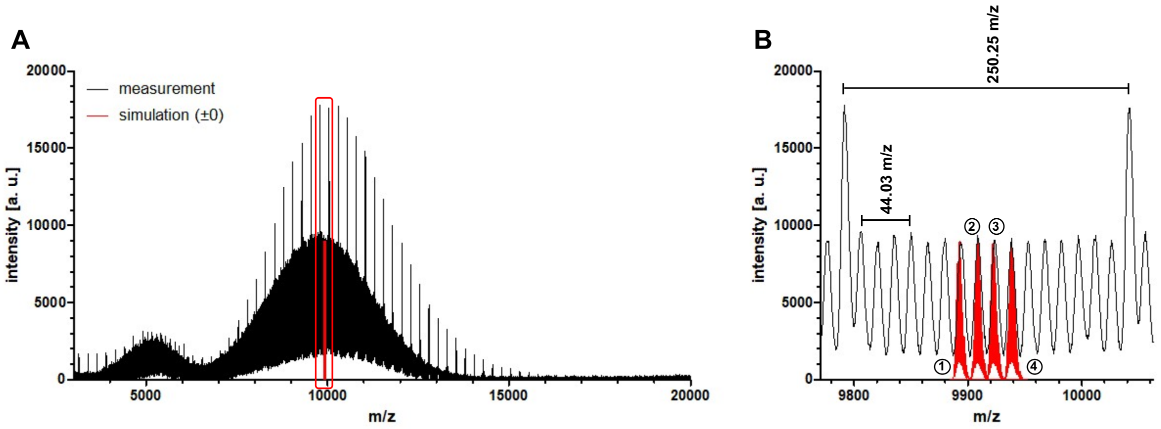

Figure S22: MALDI-ToF mass spectrum of poly(ethylene glycol)$_k$-*b*-poly(MTC-OBn)$_{21}$-(C=O)-NH-IMDQ. **A**: Full mass spectrum. The red box labels the magnified area in **B**. The simulation (red) refers to species with different block compositions (1: $k = 118$, $m = 17$, Na^+, 2: K^+, 3: $k = 113$, $m = 18$, Na^+, 4: K^+). The assigned peak distances label the mass differences corresponding to the repeating unit ethylene glycol (44.03 m/z) and MTC-OBn (250.25 m/z).

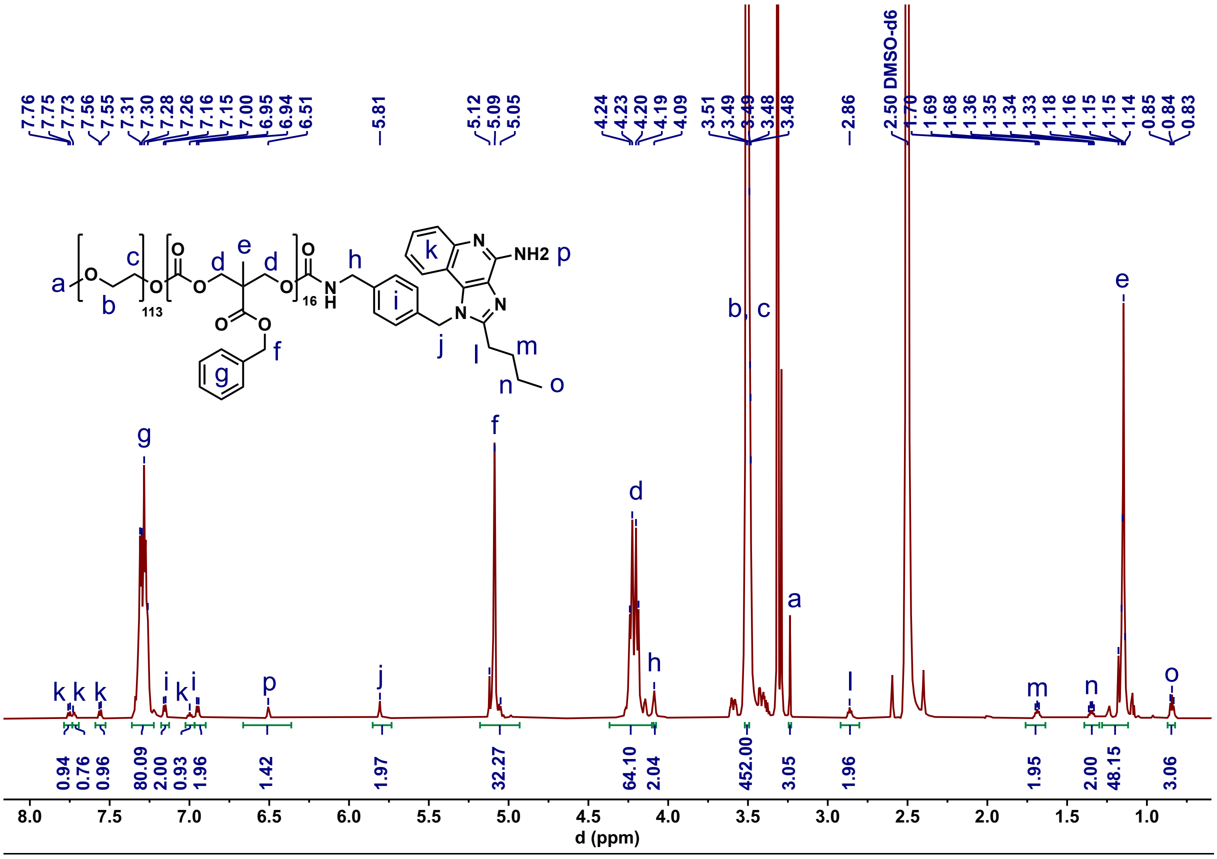

Figure S23: ^{1}H NMR spectrum (400 MHz, DMSO-d_6) of poly(ethylene glycol)$_{113}$-*b*-poly(MTC-OBn)$_{16}$-(C=O)-NH-IMDQ.

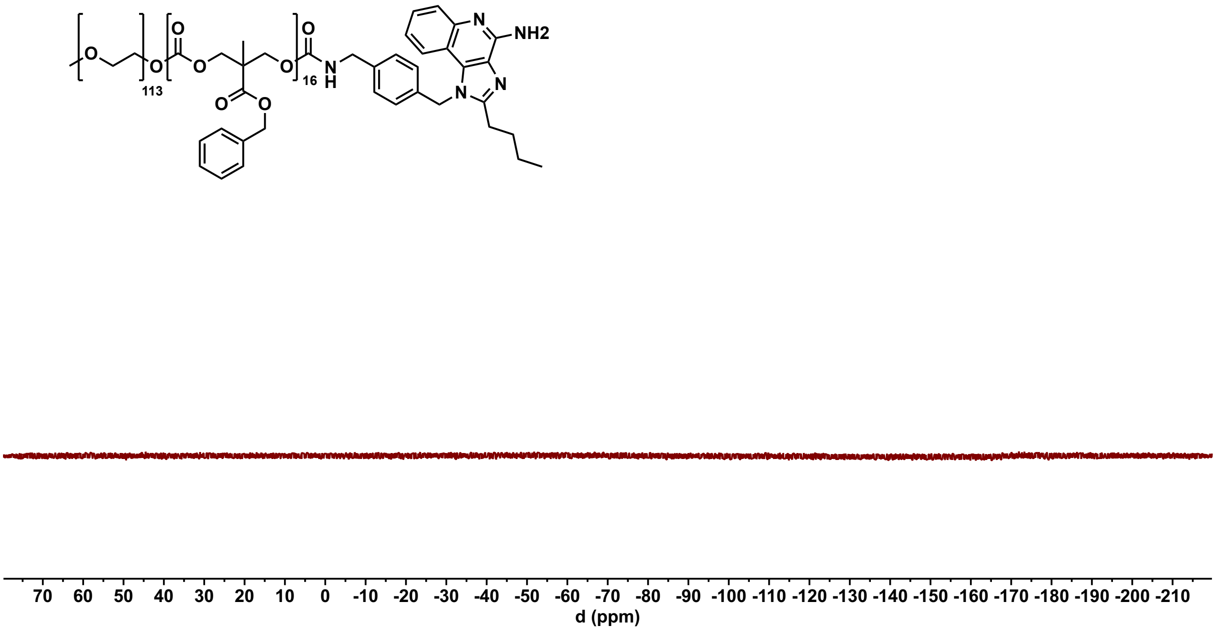

Figure S24: ^{19}F NMR spectrum (376 MHz, DMSO-d_6) of poly(ethylene glycol)$_{113}$-*b*-poly(MTC-OBn)$_{16}$-(C=O)-NH-IMDQ.

Dye Labeling

Poly(ethylene glycol)$_{113}$-*b*-poly(MTC-OEtKBn)$_n$-(C=O)-NH-TAMRA

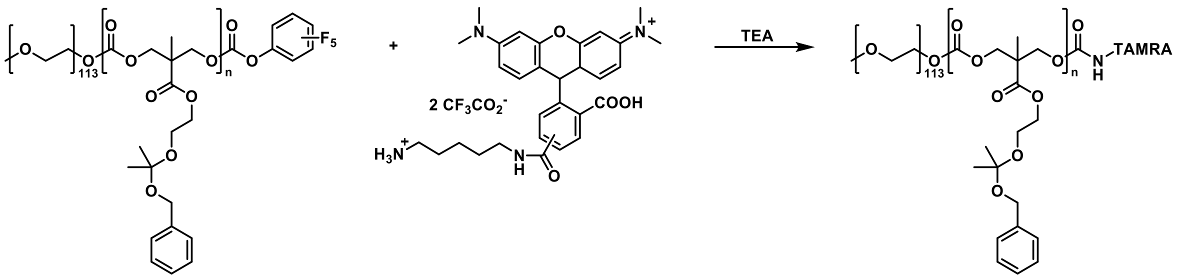

TAMRA dye-labeled poly(ethylene glycol)$_{113}$-*b*-poly(MTC-OEtKBn)$_n$-(C=O)-NH-TAMRA was synthesized from poly(ethylene glycol)$_{113}$-*b*-poly(MTC-OEtKBn)$_n$-(C=O)-O-PFP (5.0 mg, 0.67 µmol, 1.0 eq) according to the IMDQ attachment, described earlier. The dye was added to the polymer solution as 5.0 mg/mL DMSO stock solution (100 µL, 0.67 µmol, 1.0 eq) and 0,14 µL NEt_3 (1.0 µmol, 1.5 eq) were used to catalyze the reaction. The isolated dye-labeled polymer was collected as pink voluminous powder (5.0 mg, 100%).

GPC$^{UV, 540 nm}$ (HFIP, PMMA calibration): M_n = 32140 g/mol, M_w = 34810, $Đ$ = 1.08.

GPC^{RI} (HFIP, PMMA calibration): M_n = 33040 g/mol, M_w = 35780 g/mol, $Đ$ = 1.08.

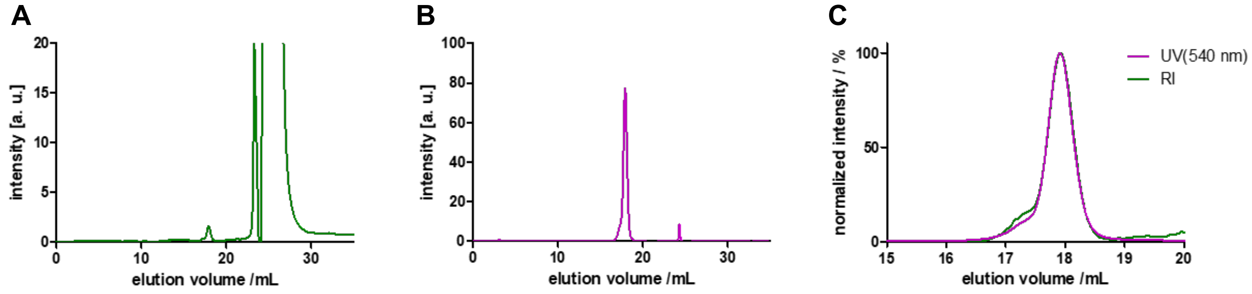

Figure S25: HFIP-GPC analysis of poly(ethylene glycol)$_{113}$-*b*-poly(MTC-OEtKBn)$_n$-(C=O)-NH-TAMRA. **A**: RI-traces and **B**: UV-traces (540 nm) of poly(ethylene glycol)$_{113}$-*b*-poly(MTC-OEtKBn)$_n$-(C=O)-NH-TAMRA. **C**: Section of the overlayed normalized UV- (540 nm, pink) and RI-signal (green) of the polymer.

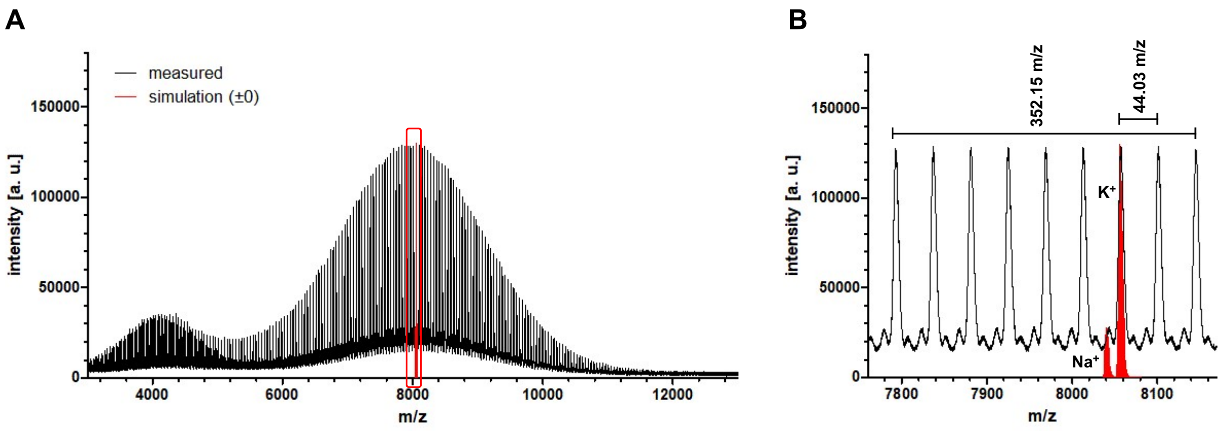

Figure S26: MALDI-ToF mass spectrum of poly(ethylene glycol)$_k$-*b*-poly(MTC-OEtKBn)$_n$-(C=O)-NH-TAMRA. **A**: Full mass spectrum. The red box labels the magnified area in **B**. The simulation (red) refers to the species with $k = 113$ and $n = 7$, cationized with Na^+ or K^+. The assigned peak distances label the mass differences corresponding to the repeating units ethylene glycol (44.03 m/z) and MTC-OEtKBn (352.15 m/z).

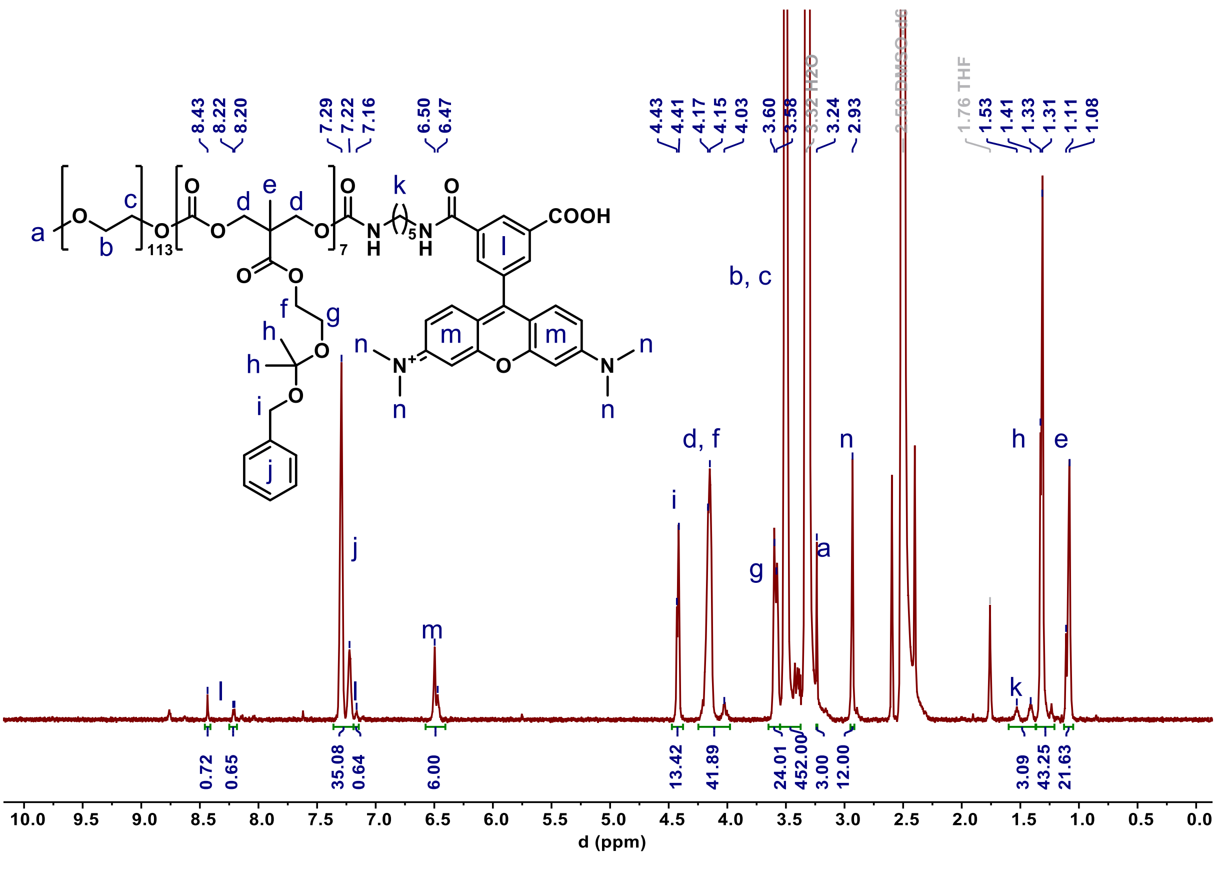

Figure S27: ^{1}H NMR spectrum (400 MHz, CD_2Cl_2) of poly(ethylene glycol)$_{113}$-*b*-poly(MTC-OEtKBn)$_7$-(C=O)-NH-TAMRA.

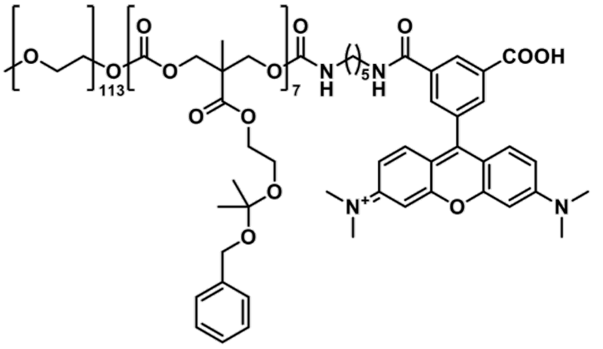

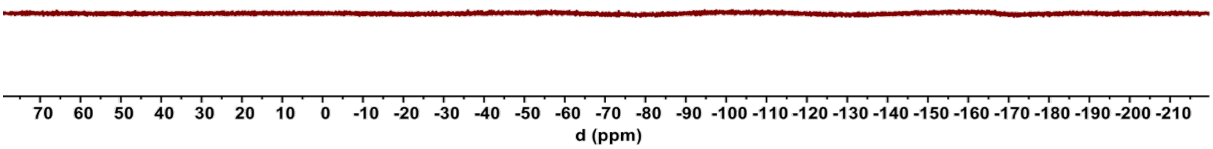

Figure S28: ^{19}F NMR spectrum (376 MHz, CD2Cl2) of poly(ethylene glycol)$_{113}$-*b*-poly(MTC-OEtKBn)$_{7}$-(C=O)-NH-TAMRA.

<u>Poly(ethylene glycol)$_{113}$-*b*-poly(MTC-OEtKBn)$_{15}$-(C=O)-NH-TAMRA</u>

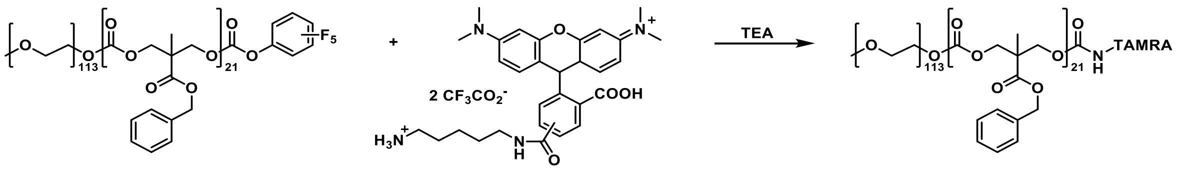

TAMRA dye-labeled poly(ethylene glycol)$_{113}$-*b*-poly(MTC-OBn)$_{21}$-(C=O)-NH-TAMRA was gratefully provided by CHRISTIAN CZYSCH. Its characterization is described in the literature.[2]

GPC^{RI} (HFIP, PMMA calibration): M_n = 30690 g/mol, M_w = 31700 g/mol, $Đ$ = 1.03.

Poly(ethylene glycol)$_{113}$-*b*-poly(MTC-OEtKBn)$_{19}$-(C=O)-NH-Cy3

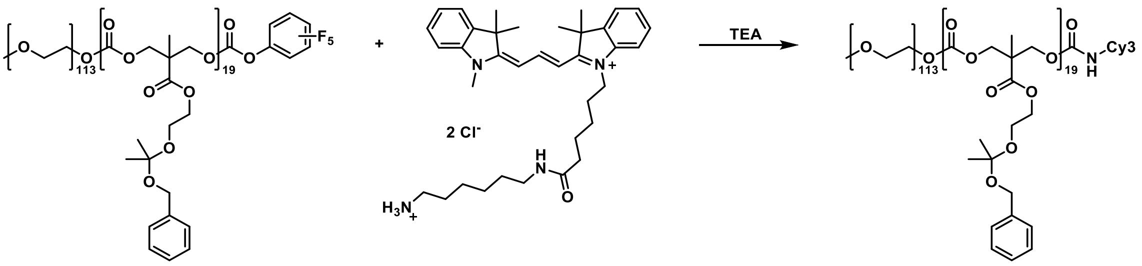

Cy3-labeled poly(ethylene glycol)$_{113}$-*b*-poly(MTC-OEtKBn)$_{19}$-(C=O)-NH-Cy3 was synthesized from poly(ethylene glycol)$_{113}$-*b*-poly(MTC-OEtKBn)$_{19}$-(C=O)-O-PFP (10.0 mg, 0.83 µmol, 1.0 eq) according to the IMDQ attachment, described earlier. The dye was added to the polymer solution as 25 mg/mL DMSO stock solution (21.1 µL, 0.83 µmol, 1.0 eq) and 0,17 µL NEt_3 (1.3 µmol, 1.5 eq) were used to catalyze the reaction. The isolated dye-labeled polymer was collected as red voluminous powder (10.3 mg, 100%).

GPC$^{UV, 540 nm}$ (HFIP, PMMA calibration): M_n = 39280 g/mol, M_w = 40790 g/mol, $Đ$ = 1.04.

GPC^{RI} (HFIP, PMMA calibration): M_n = 38470 g/mol, M_w = 40020 g/mol, $Đ$ = 1.04.

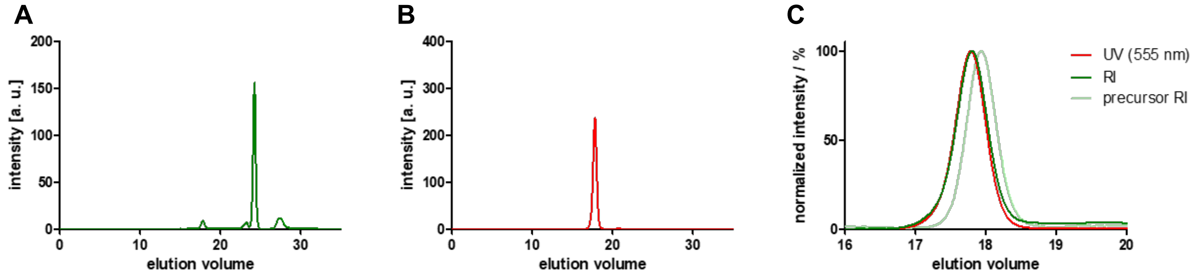

Figure S29: HFIP-GPC analysis of poly(ethylene glycol)$_{113}$-*b*-poly(MTC-OEtKBn)$_{19}$-(C=O)-NH-Cy3. **A**: RI-traces and **B**: UV-traces (555 nm) of poly(ethylene glycol)$_{113}$-*b*-poly(MTC-OEtKBn)$_{19}$-(C=O)-NH-Cy3. **C**: Section of the overlayed normalized UV- (555 nm, red) and RI-signal (green) of the polymer peak next to the precursor RI-signal (light green).

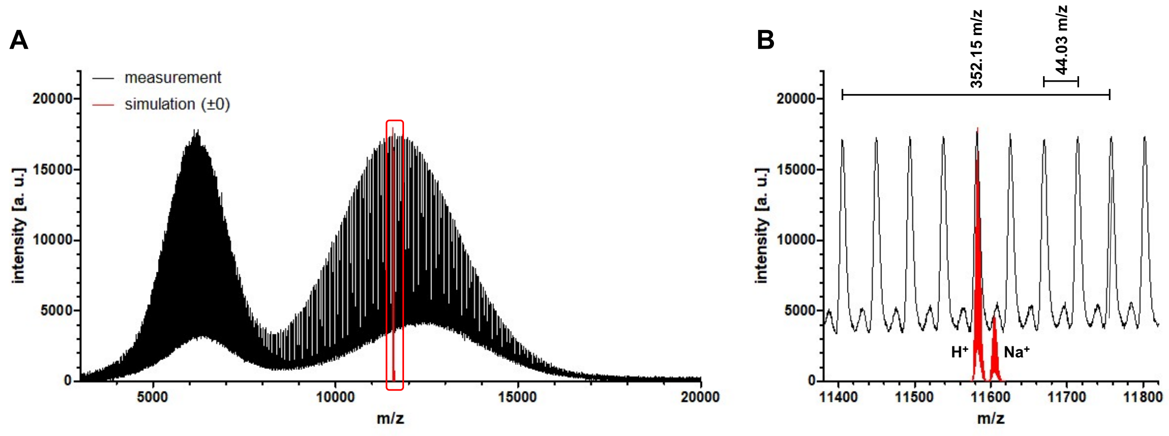

Figure S30: MALDI-ToF mass spectrum of poly(ethylene glycol)$_k$-*b*- poly(MTC-OEtKBn)$_{19}$-(C=O)-NH-Cy3. **A**: Full mass spectrum. The red box labels the magnified area in **B**. The simulation (red) refers to the species with k = 113 and n = 17, cationized with H^+ or Na^+. The assigned peak distances label the mass differences corresponding to the repeating units ethylene glycol (44.03 m/z) and MTC-OEtKBn (352.15 m/z).

Poly(ethylene glycol)$_{113}$-*b*-poly(MTC-OBn)$_{21}$-(C=O)-NH-Cy3

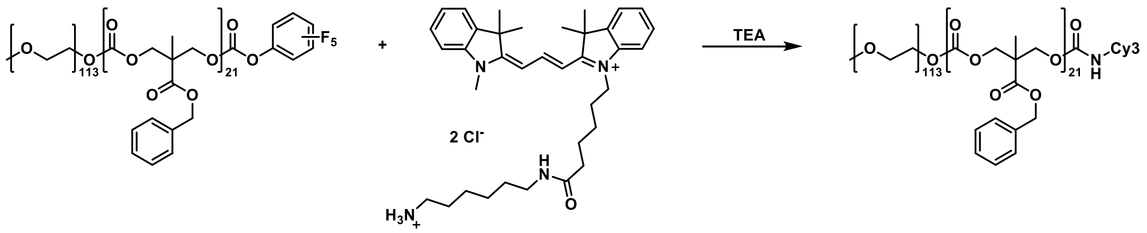

Cy3-labeled poly(ethylene glycol)$_{113}$-*b*-poly(MTC-OBn)$_{21}$-(C=O)-NH-Cy3 was synthesized from poly(ethylene glycol)$_{113}$-*b*-poly(MTC-OBn)$_{21}$-(C=O)-O-PFP (10.0 mg, 0.93 µmol, 1.0 eq) according to the IMDQ attachment, described earlier. The dye was added to the polymer solution as 25 mg/mL DMSO stock solution (23.4 µL, 0.93 µmol, 1.0 eq) and 0,19 µL NEt_3 (1.4 µmol, 1.5 eq) were used to catalyze the reaction. The isolated dye-labeled polymer was collected as red voluminous powder (10.5 mg, 100%).

GPC$^{UV, 540\ nm}$ (HFIP, PMMA calibration): M_n = 38580 g/mol, M_w = 40120 g/mol, $Đ$ = 1.04.

GPC^{RI} (HFIP, PMMA calibration): M_n = 37960 g/mol, M_w = 39540 g/mol, $Đ$ = 1.04.

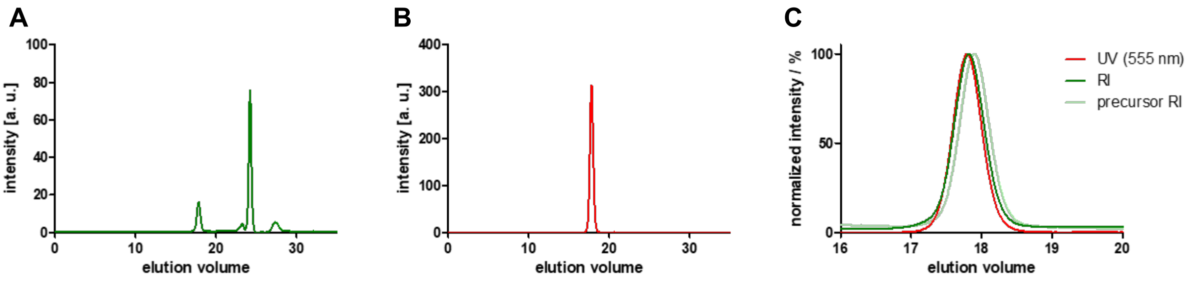

Figure S31: HFIP-GPC analysis of poly(ethylene glycol)$_{113}$-*b*-poly(MTC-OBn)$_{21}$-(C=O)-NH-Cy3. **A**: RI-traces and **B**: UV-traces (555 nm) of poly(ethylene glycol)$_{113}$-*b*-poly(MTC-OBn)$_{21}$-(C=O)-NH-Cy3. **C**: Section of the overlayed normalized UV-(555 nm, red) and RI-signal (green) of the polymer peak next to the precursor RI-signal (light green).

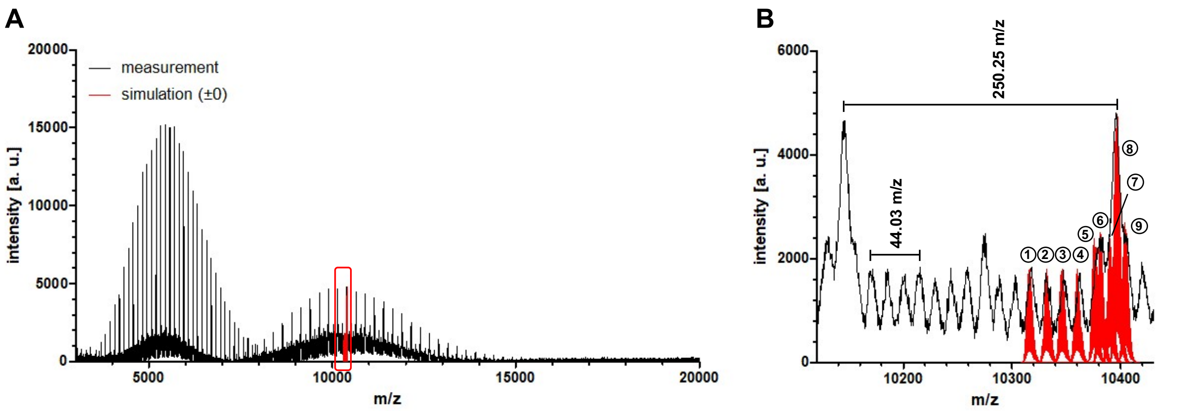

Figure S32: MALDI-ToF mass spectrum of poly(ethylene glycol)$_k$-*b*-poly(MTC-OBn)$_m$-(C=O)-NH-Cy3 (**2.3**). **A**: Full mass spectrum. The red box labels the magnified area in **B**. The simulation (red) refers to species with different block compositions (1: k = 118, m = 18, 2: k = 107, m = 20, 3: k = 113, m = 19, 4: k = 119, m = 18, 5: k = 108, m = 20, 6: k = 113, m = 19, +HCl, 7: k = 114, m = 19, 8: k = 119, m = 18, +HCl, 9: k = 120, m = 18, all without additional cation). The assigned peak distances label the mass difference corresponding to the repeating unit ethylene glycol (44.03 m/z) and MTC-OBn (250.25 m/z).

Poly(ethylene glycol)$_{113}$-*b*-poly(MTC-OEtKBn)$_{19}$-(C=O)-NH-Cy5

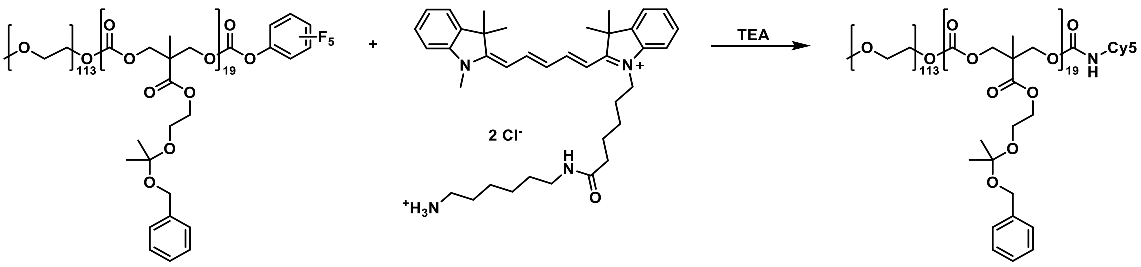

Cy5-labeled poly(ethylene glycol)$_{113}$-*b*-poly(MTC-OEtKBn)$_{19}$-(C=O)-NH-Cy5 (was synthesized from poly(ethylene glycol)$_{113}$-*b*-poly(MTC-OEtKBn)$_{19}$-(C=O)-O-PFP (10.0 mg, 0.83 µmol, 1.0 eq) according to the IMDQ attachment, described earlier. The dye was added to the polymer solution as 25 mg/mL DMSO stock solution (21.9 µL, 0.83 µmol, 1.0 eq) and 0,17 µL NEt_3 (1.3 µmol, 1.5 eq) were used to catalyze the reaction. The isolated dye-labeled polymer was collected as blue voluminous powder (10.2 mg, 100%).

GPC$^{UV,\ 600\ nm}$ (HFIP, PMMA calibration): M_n = 37600 g/mol, M_w = 39470 g/mol, $Đ$ = 1.05.

GPC^{RI} (HFIP, PMMA calibration): M_n = 36340 g/mol, M_w = 38410 g/mol, $Đ$ = 1.06.

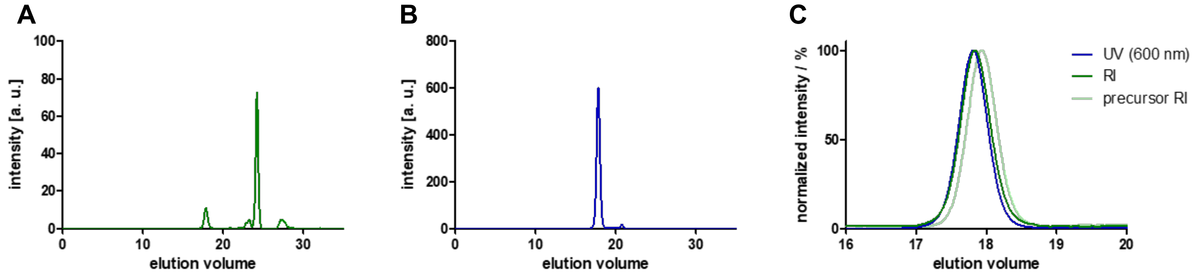

Figure S33: HFIP-GPC analysis of poly(ethylene glycol)$_{113}$-*b*-poly(MTC-OEtKBn)$_{19}$-(C=O)-NH-Cy5. **A**: RI-traces and **B**: UV-traces (600 nm) of poly(ethylene glycol)$_{113}$-*b*-poly(MTC-OEtKBn)$_{19}$-(C=O)-NH-Cy5. **C**: Section of the overlayed normalized UV- (600 nm, blue) and RI-signal (green) of the polymer peak next to the precursor RI-signal (light green).

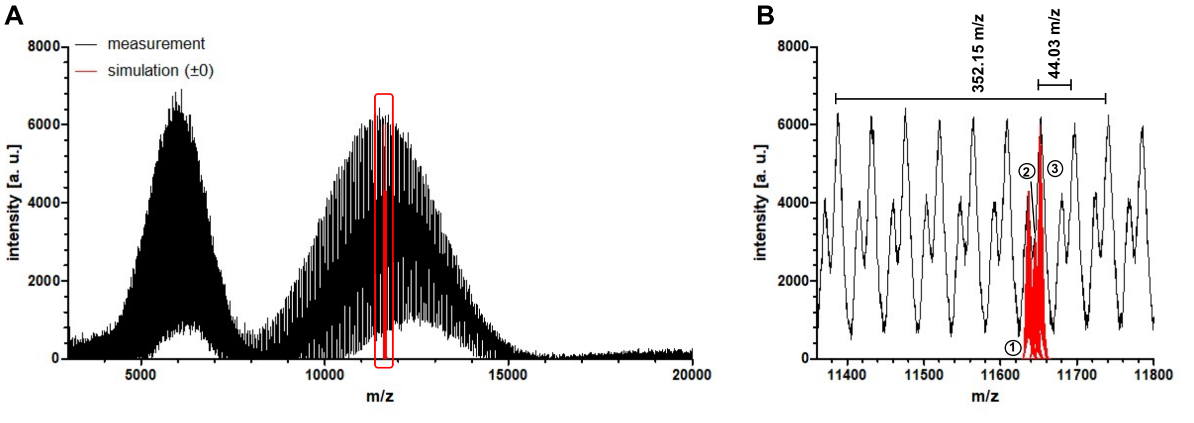

Figure S34: MALDI-ToF mass spectrum of poly(ethylene glycol)$_k$-*b*- poly(MTC-OEtKBn)$_{19}$-(C=O)-NH-Cy5. **A**: Full mass spectrum. The red box labels the magnified area in **B**. The simulation (red) refers to peaks with different block compositions (1: k = 112, n = 17, +2HCl, 2: k = 113, n = 17, +HCl, 3: k = 114, n = 17, all without additional cation). The assigned peak distances label the mass differences corresponding to the repeating units ethylene glycol (44.03 m/z) and MTC-OEtKBn (352.15 m/z).

Poly(ethylene glycol)$_{113}$-*b*-poly(MTC-OBn)$_{21}$-(C=O)-NH-Cy5

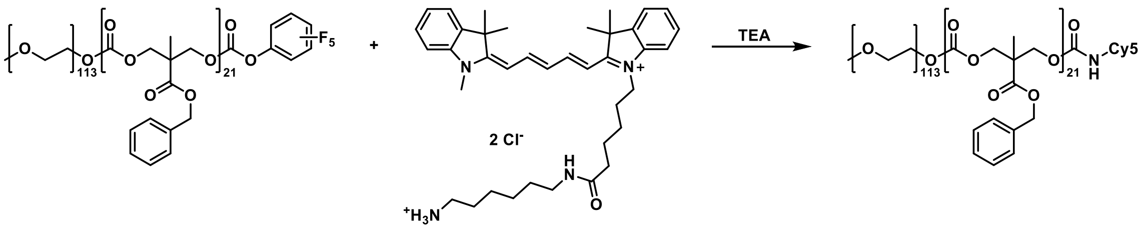

Cy5-labeled poly(ethylene glycol)$_{113}$-*b*-poly(MTC-OBn)$_{21}$-(C=O)-NH-Cy5 was synthesized from poly(ethylene glycol)$_{113}$-*b*-poly(MTC-OBn)$_{21}$-(C=O)-O-PFP (10.0 mg, 0.93 µmol, 1.0 eq) according to the IMDQ attachment, described earlier. The dye was added to the polymer solution as 25 mg/mL DMSO stock solution (24.4 µL, 0.93 µmol, 1.0 eq) and 0,19 µL NEt_3 (1.4 µmol, 1.5 eq) were used to catalyze the reaction. The isolated dye-labeled polymer was collected as blue voluminous powder (6.5 mg, 65%).

GPC$^{UV, 600 nm}$ (HFIP, PMMA calibration): M_n = 37270 g/mol, M_w = 38930 g/mol, $Đ$ = 1.05.

GPC^{RI} (HFIP, PMMA calibration): M_n = 35940 g/mol, M_w = 37740 g/mol, $Đ$ = 1.05.

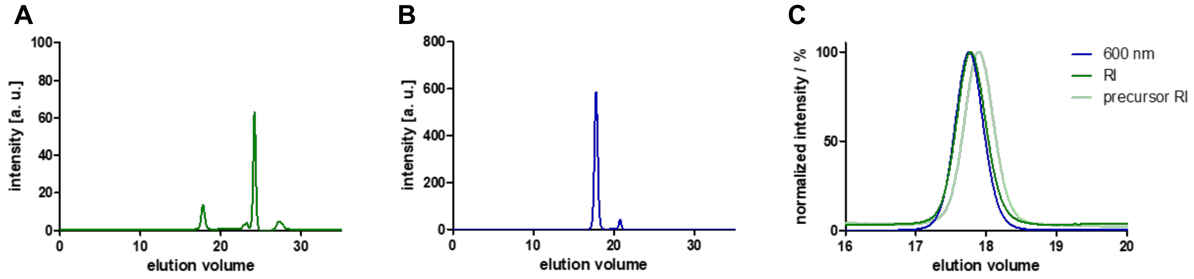

Figure S35: HFIP-GPC analysis of poly(ethylene glycol)$_{113}$-*b*-poly(MTC-OBn)$_{21}$-(C=O)-NH-Cy5. **A**: RI-traces and **B**: UV-traces (600 nm) of poly(ethylene glycol)$_{113}$-*b*-poly(MTC-OBn)$_{21}$-(C=O)-NH-Cy5. **C**: Section of the overlayed normalized UV-(600 nm, blue) and RI-signal (green) of the polymer peak next to the precursor RI-signal (light green).

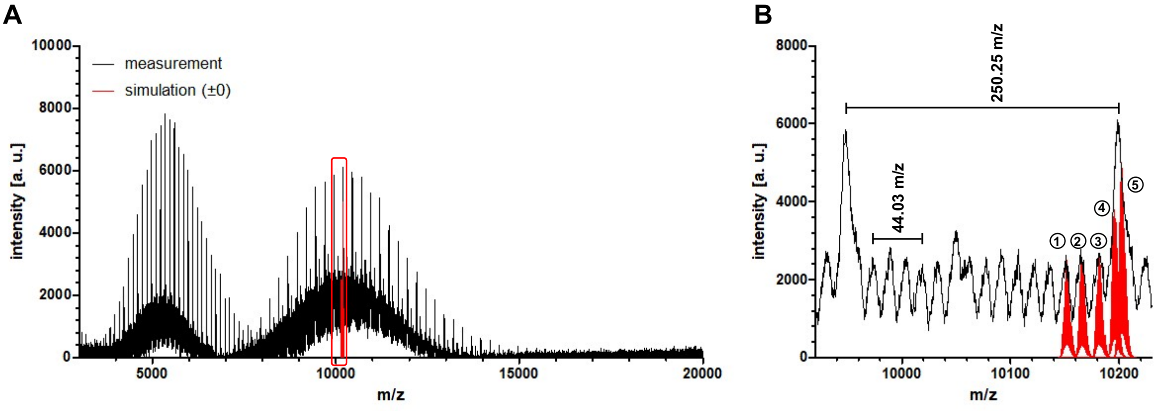

Figure S36: MALDI-ToF mass spectrum of poly(ethylene glycol)$_k$-*b*-poly(MTC-OBn)$_m$-(C=O)-NH-Cy5 (**2.4**). **A**: Full mass spectrum. The red box labels the magnified area in **B**. The simulation (red) refers to peaks with different block compositions (1: k = 108, m = 19, 2: k = 114, m = 18, 3: k = 103, m = 20, 4: k = 109, m = 19, 5: k = 114, m = 18, +HCl, all without additional cation). The assigned peak distances label the mass difference corresponding to the repeating unit ethylene glycol (44.03 m/z) and MTC-OBn (250.25 m/z).

Poly(ethylene glycol)$_{113}$-*b*-poly(MTC-OEtKBn)$_{19}$-(C=O)-NH-IRDye800RS

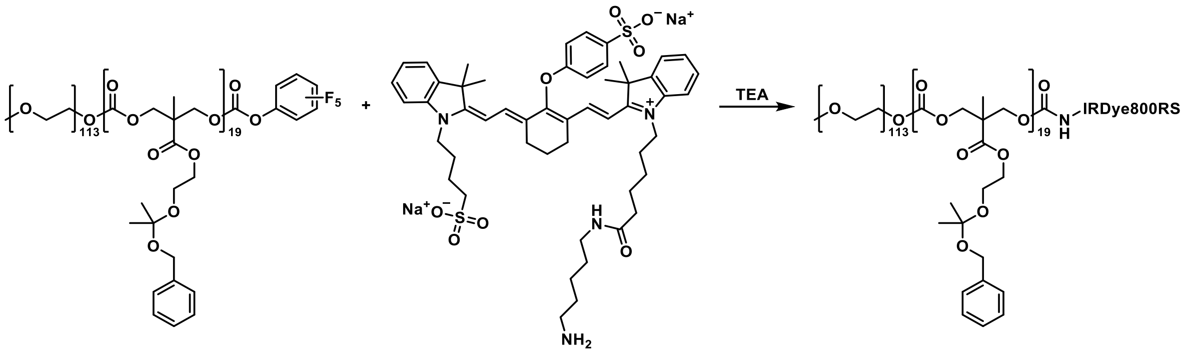

IRDye00RS-labeled poly(ethylene glycol)$_{113}$-*b*-poly(MTC-OEtKBn)$_{19}$-(C=O)-NH-IRDye800RS was synthesized from poly(ethylene glycol)$_{113}$-*b*-poly(MTC-OEtKBn)$_{19}$-(C=O)-O-PFP (10.0 mg, 0.83 µmol, 1.0 eq) according to the IMDQ attachment, described earlier. The dye was added to the polymer solution as 25 mg/mL DMSO stock solution (31.9 µL, 0.83 µmol, 1.0 eq) and 0,17 µL NEt_3 (1.3 µmol, 1.5 eq) were used to catalyze the reaction. The isolated dye-labeled polymer was further purified by spin filtration (10 kDa MWCO), to remove traces of unreacted dye. The labeled polymer was collected as voluminous green powder (10.1 mg, 100%).

GPC$^{UV,\ 600\ nm}$ (HFIP, PMMA calibration): M_n = 29310 g/mol, M_w = 30780 g/mol, $Đ$ = 1.05.

GPC^{RI} (HFIP, PMMA calibration): M_n = 27260 g/mol, M_w = 28550 g/mol, $Đ$ = 1.05.

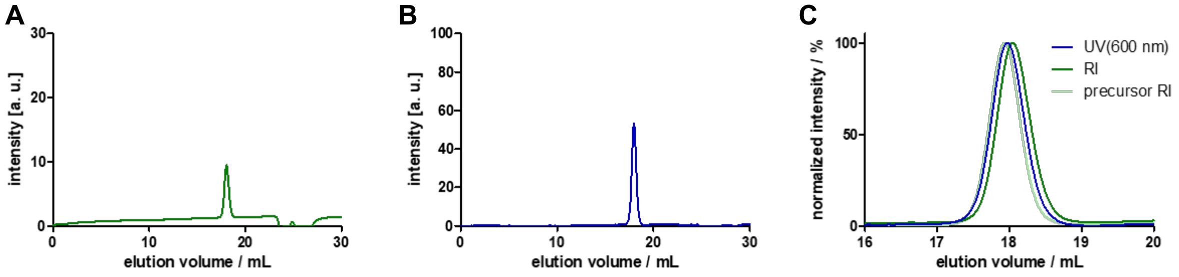

Figure S37: HFIP-GPC analysis of poly(ethylene glycol)$_{113}$-*b*-poly(MTC-OEtKBn)$_{19}$-(C=O)-NH-IRDye800RS. **A**: RI-traces and **B**: UV-traces (600 nm) of poly(ethylene glycol)$_{113}$-*b*-poly(MTC-OEtKBn)$_{19}$-(C=O)-NH-IRDye800RS. **C**: Section of the overlayed normalized UV- (600 nm, blue) and RI-signal (green) of the polymer peak next to the precursor RI-signal (light green).

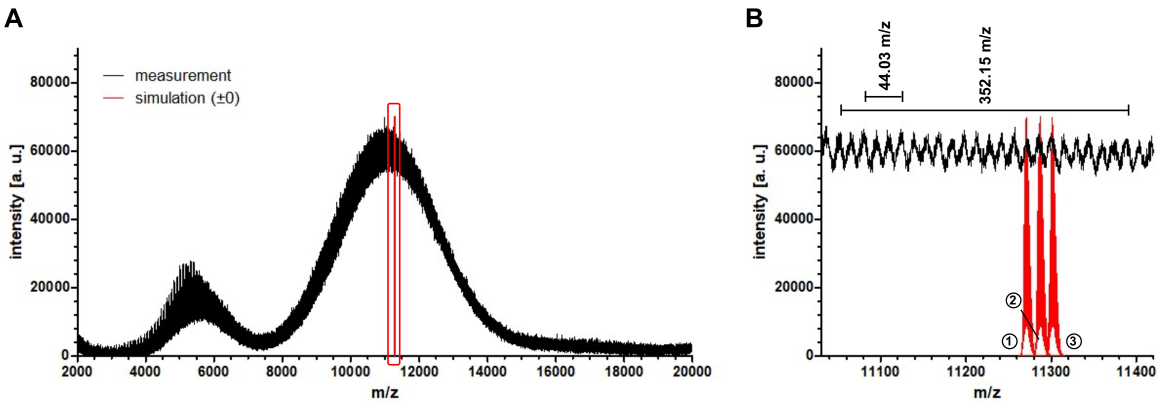

Figure S38: MALDI-ToF mass spectrum of poly(ethylene glycol)$_k$-*b*- poly(MTC-OEtKBn)$_{19}$-(C=O)-NH-IRDye800RS (**2.5**). **A**: Full mass spectrum. The red box labels the magnified area in **B**. The simulation (red) refers to peaks with different block compositions (1: $k = 113$, $n = 15$, Na^+, H^+, 2: $k = 113$, $n = 15$, K^+, H^+, 3: $k = 113$, $n = 17$, +HCl, Na^+, K^+). The assigned peak distances label the mass differences corresponding to the repeating units ethylene glycol (44.03 m/z) and MTC-OEtKBn (352.15 m/z).

Poly(ethylene glycol)$_{113}$-*b*-poly(MTC-OBn)$_{21}$-(C=O)-NH-IRDye800RS

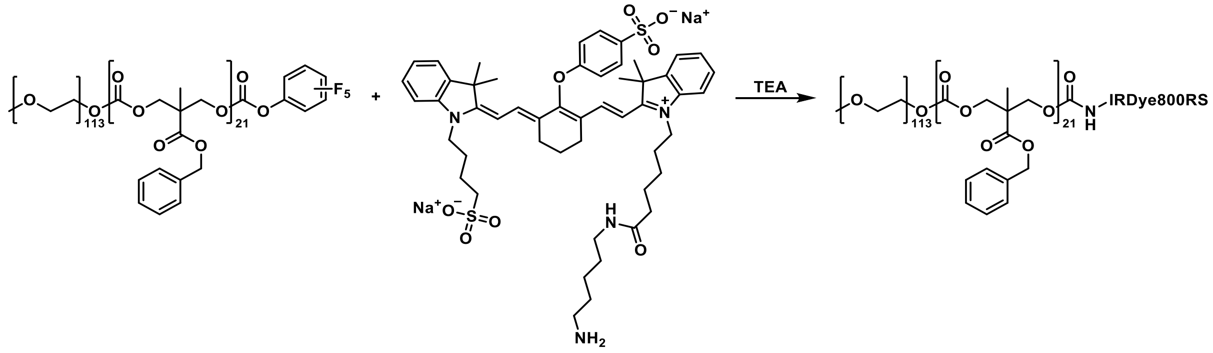

IRDye00RS-labeled poly(ethylene glycol)$_{113}$-*b*-poly(MTC-OBn)$_{21}$-(C=O)-NH-IRDye00RS was synthesized from poly(ethylene glycol)$_{113}$-*b*-poly(MTC-OBn)$_{21}$-(C=O)-O-PFP (10.0 mg, 0.93 µmol, 1.0 eq) according to the IMDQ attachment, described earlier. The dye was added to the polymer solution as 25 mg/mL DMSO stock solution (35.4 µL, 0.93 µmol, 1.0 eq) and 0,19 µL NEt_3 (1.4 µmol, 1.5 eq) were used to catalyze the reaction. The isolated dye-labeled polymer was further purified by spin filtration (10 kDa MWCO), to remove traces of unreacted dye. The labeled polymer was collected as green voluminous powder (10.3 mg, 100%).

GPC$^{UV, 600\ nm}$ (HFIP, PMMA calibration): $M_n = 28030$ g/mol, $M_w = 29980$ g/mol, $Đ = 1.07$.

GPC^{RI} (HFIP, PMMA calibration): $M_n = 26930$ g/mol, $M_w = 28490$ g/mol, $Đ = 1.06$.

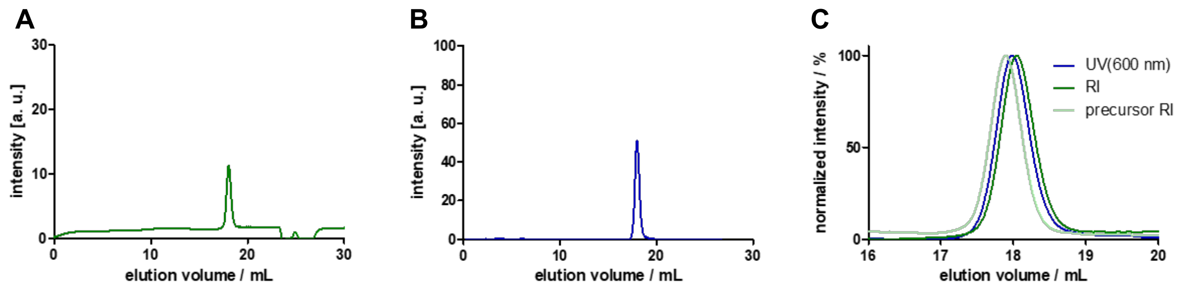

Figure S39: HFIP-GPC analysis of poly(ethylene glycol)$_{113}$-*b*-poly(MTC-OBn)$_{21}$-(C=O)-NH-IRDye800RS. **A**: RI-traces and **B**: UV-traces (600 nm) of poly(ethylene glycol)$_{113}$-*b*-poly(MTC-OBn)$_{21}$-(C=O)-NH- IRDye800RS (**2.5**). **C**: Section of the overlayed normalized UV- (600 nm, blue) and RI-signal (green) of the polymer peak next to the precursor RI-signal (light green).

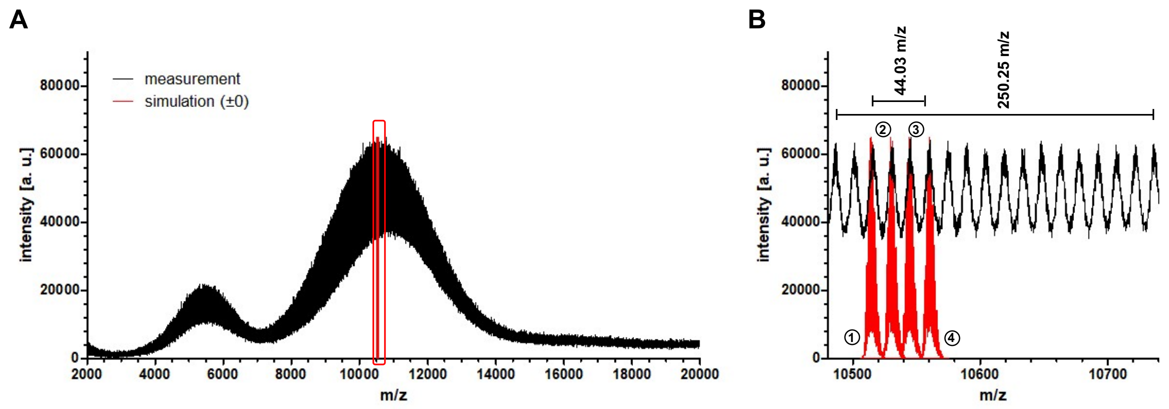

Figure S40: MALDI-ToF mass spectrum of poly(ethylene glycol)$_{113}$-*b*-poly(MTC-OBn)$_{21}$-(C=O)-NH-IRDye800RS. **A**: Full mass spectrum. The red box labels the magnified area in **B**. The simulation (red) refers to peaks with different block compositions (1: $k = 118$, $m = 17$, 2: $k = 107$, $m = 19$, 3: $k = 113$, $m = 18$, 4: $k = 102$, $m = 20$, all with two H^+). The assigned peak distances label the mass difference corresponding to the repeating unit ethylene glycol (44.03 m/z) and MTC-OBn (250.25 m/z).

NMR SIDE CHAIN DEGRADATION STUDY

NMR monitoring of acidic side group hydrolysis of poly(ethylene glycol)$_{113}$-*b*-poly(MTC-OEtKBn)$_{19}$

5.0 mg of poly(ethylene glycol)$_{113}$-*b*-poly(MTC-OEtKBn)$_{19}$ were weighed into a snap cap vial and dissolved with 0.33 mL of D_2O, supplemented with 0.6 mg of dimethylsulfone as internal standard. The polymers were quickly self-assembled by ultrasonification. Subsequently, the sample was diluted with 0.33 mL of a 0.8 mM deuterated phosphate buffer where the pD was adjusted to a value of 5.5 determined by standard pH glass electrode. According to literature reports,[4] this would correspond to a realistic pH value of around 6.0. The sample was immediately transferred into an NMR tube and subsequently monitored over time at the given time points, while stored at 37°C in order to record the hydrolysis of the benzyl ketal side group and the release of benzyl alcohol and acetone.

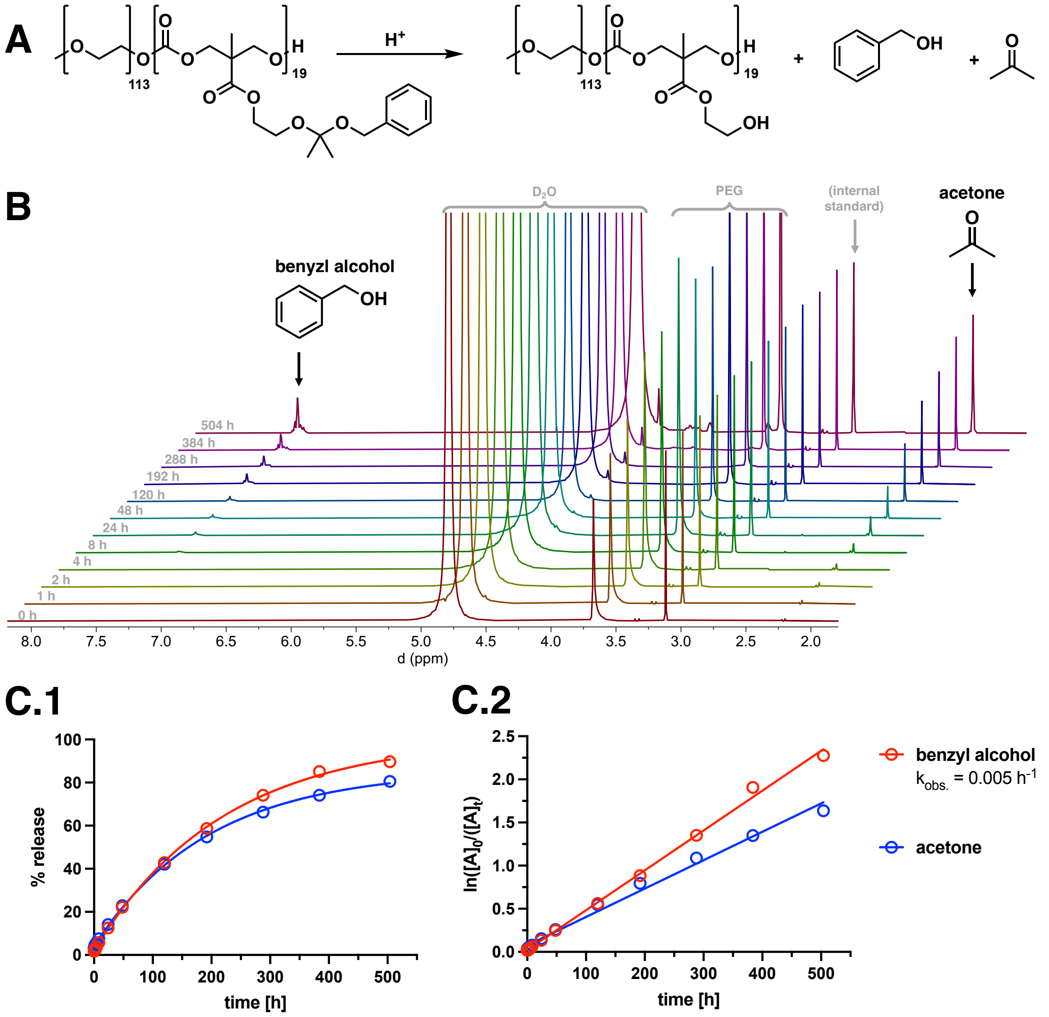

Figure S41: ^{1}H NMR side chain degradation study of self-assembled poly(ethylene glycol)$_k$-*b*- poly(MTC-OEtKBn)$_{19}$-block copolymers at 37°C in deuterated phosphate buffer at pD 5.9. **A:** Chemical structures of the ketal hydrolysis at low pH conditions. **B:** Recorded ^{1}H NMR spectra where the proton signals of the liberated benzyl alcohol and acetone were monitored over time (in relation the the internal standard dimethylsulfone). **C.1:** Evaluation of the released ketal degradation products benzyl alcohol and acetone – the lower amount of recorded acetone protons is probably related to its low vapor pressure). **C.2:** The exponential decay constant $k_{obs.}$ is therefore derived from the released benzyl alcohol and the slop of the logarithmic plot of its ^{1}H NMR integrals against time. When applying the obtained exponential decay constant k_{obs} to the equation determined by the FRET measurements of *Figure S63* ($log(k_{obs}) = 2.86 - 0.881*pH$), one can obtain pH = 5.85, which corresponds to the estimated pD value.

NMR BACKBONE DEGRADATION STUDY

Acidic side group hydrolysis of poly(ethylene glycol)$_{113}$-*b*-poly(MTC-OEtKBn)$_{19}$

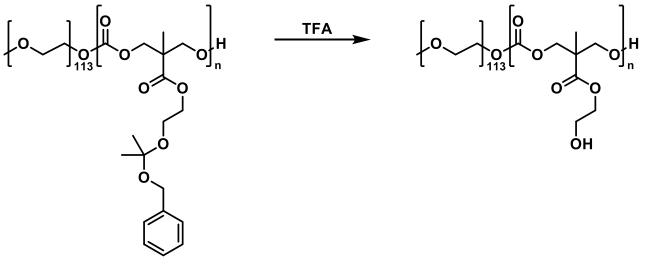

An NMR tube was charged with 10 mg poly(ethylene glycol)$_{113}$-*b*-poly(MTC-OEtKBn)$_{19}$ dissolved in 1.0 mL CD_2Cl_2 and the sample was analyzed by ^{1}H NMR spectroscopy. Afterwards, 5.0 µL trifluoroacetic acid (TFA) were added and another ^{1}H NMR spectrum was measured, confirming instant ketal hydrolysis under the formation of acetone and benzyl alcohol. The side group degraded polymer was precipitated in ice-cold diethyl ether and isolated by centrifugation (4000 rpm, 0 °C, 20 min). Afterwards, it was freeze dried from benzene (instead of benzene, anhydrous toluene can also be applied for removing traces of water azeotropically[3]), yielding a white amorphous solid (9.5 mg, .98%).

^{1}H NMR (300 MHz, CD_2Cl_2) δ [ppm]: 4.36–4.27 (m, 4*n*H, -O-(C=O)-O-C*H*$_2$-C-C*H*$_2$-) 4.25–4.22 (m, 2*n*H, HO-CH$_2$-C*H*$_2$-); 3.76 (m, 2*n*H, HO-C*H*$_2$-); 3.60 (m, 452H mPEG-*H*); 3.34 (s, 3H, C*H*$_3$-PEG); 2.84 (s, 1H, *H*O-); 1.28–1.20 (m, 3*n*H, mPEG$_{113}$-O-(C=O)-O-CH$_2$-C-C*H*$_3$).

GPC^{RI} (THF, PS calibration): M_n = 6837 g/mol, M_w = 7073 g/mol $Đ$ = 1.03.

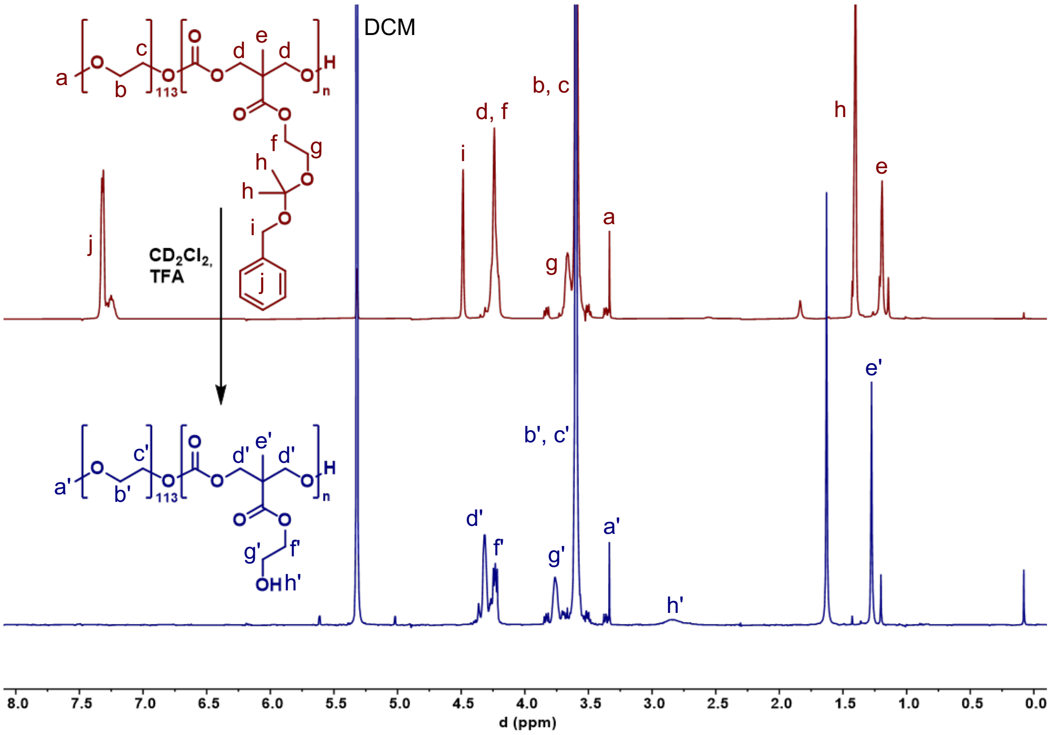

Figure S42: Side group degradation of poly(ethylene glycol)$_{113}$-*b*-poly(MTC-OEtKBn)$_{19}$ by ketal hydrolysis with TFA in d$_2$-DCM. The red structure and ^{1}H NMR spectrum (300 MHz, CD_2Cl_2) correspond to the starting material before TFA addition. In blue, the isolated product structure and its ^{1}H NMR spectrum (300 MHz, CD_2Cl_2) are shown.

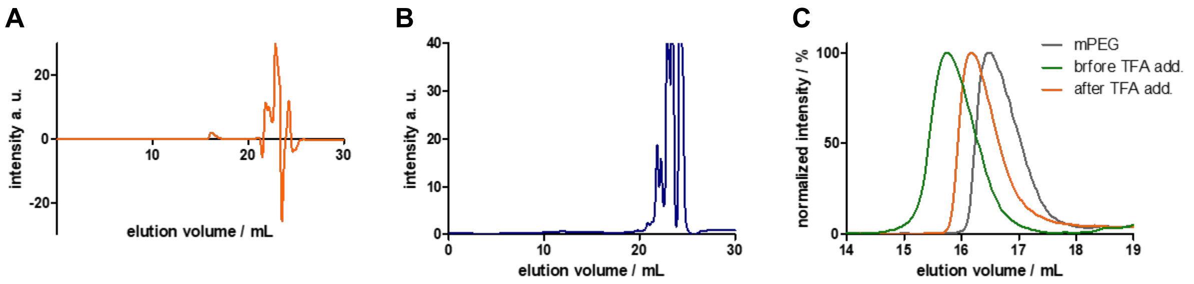

Figure S43: THF-GPC analysis of poly(ethylene glycol)$_{113}$-*b*-poly(MTC-OEtOH)$_{19}$. **A**: RI-traces and **B**: UV-traces of poly(ethylene glycol)$_{113}$-*b*-poly(MTC-OEtOH)$_{19}$; since no aromatic groups are present anymore, no UV signal can be found. **C**: Section of the overlayed normalized RI-signal of the staring material (green) and the hydrophilized product (orange) next to mPEG$_{113}$-OH (grey).

Acidic treatment of poly(ethylene glycol)$_{113}$-*b*-poly(MTC-OBn)$_m$

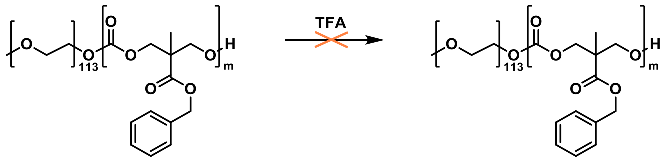

An NMR tube was charged with 10 mg poly(ethylene glycol)$_{113}$-*b*-poly(MTC-OBn)$_{21}$ dissolved in 1.0 mL CD_2Cl_2 and the sample was analyzed by ^{1}H NMR spectroscopy. Afterwards, 5.0 µL trifluoroacetic acid (TFA) were added and another ^{1}H NMR spectrum was measured. The retained polymer was precipitated in ice-cold diethyl ether and isolated by centrifugation (4000 rpm, 0 °C, 20 min). Afterwards, it was freeze dried from benzene (instead of benzene, anhydrous toluene can also be applied for removing traces of water azeotropically[3]), yielding a white amorphous solid (9.8 mg, 98%).

^{1}H NMR (300 MHz, CD_2Cl_2) δ[ppm]: 7.33–7.31 (m, 5*m*H, Ar-*H*); 5.15–5.13 (m, 2*m*H, Ar-CH_2-); 4.28–4.24 (m, 4*m*H, -O-(C=O)-O-CH_2-C-CH_2-); 3.71 (m, 452H mPEG-*H*); 3.47 (s, 3H, CH_3-PEG); 1.27–1.23 (m, 3*m*H, -O-(C=O)-O-CH$_2$-C-CH_3).

GPC^{UV} (THF, PS calibration): M_n = 10530 g/mol, M_w = 10860 g/mol, $Đ$ = 1.03.

GPC^{RI} (THF, PS calibration): M_n = 10200 g/mol, M_w = 10680 g/mol, $Đ$ = 1.05.

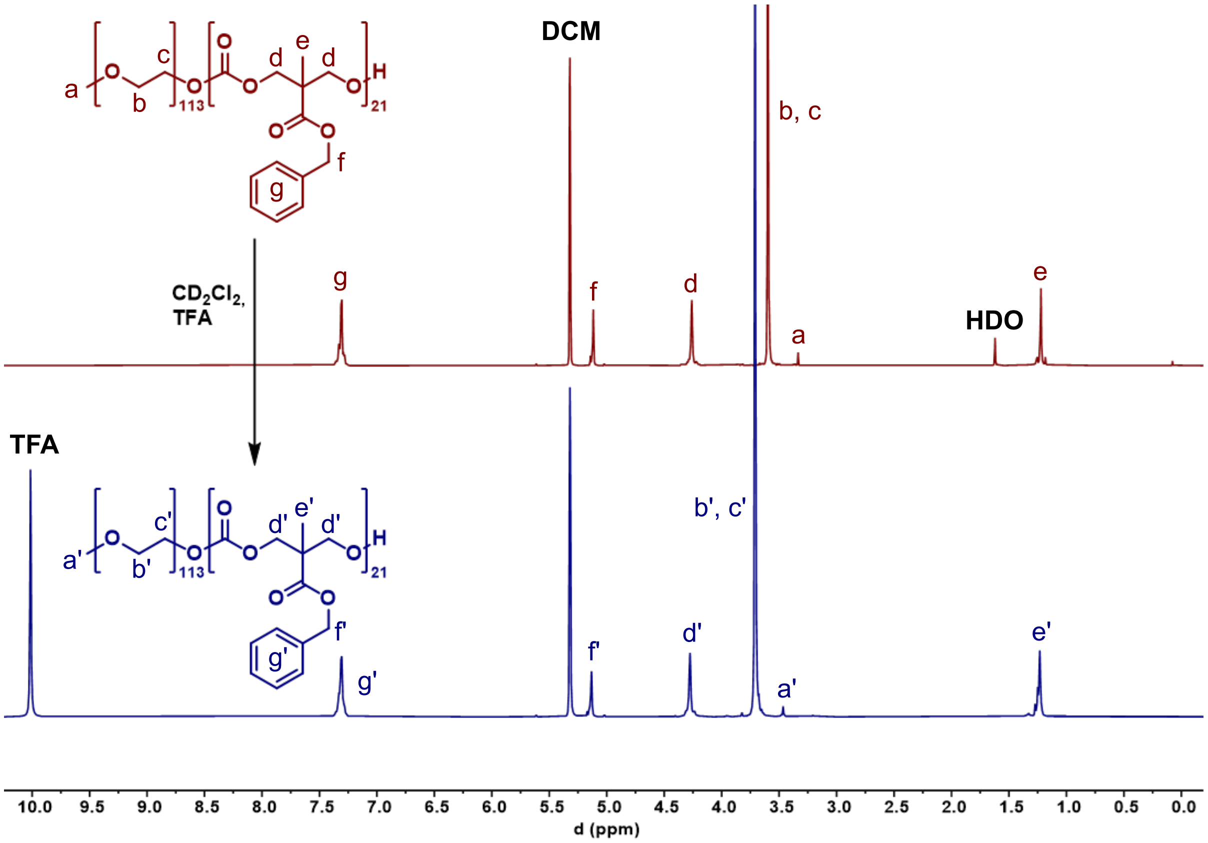

Figure S44: Treatment of poly(ethylene glycol)$_{113}$-*b*-poly(MTC-OBn)$_m$ with trifluoroacetic acid (TFA) in d_2-DCM. The red structure and ^{1}H NMR spectrum (300 MHz, CD_2Cl_2) correspond to the starting material block copolymer before TFA addition. In blue, the recovered structure and the corresponding ^{1}H NMR spectrum (300 MHz, CD_2Cl_2) after TFA addition are shown.

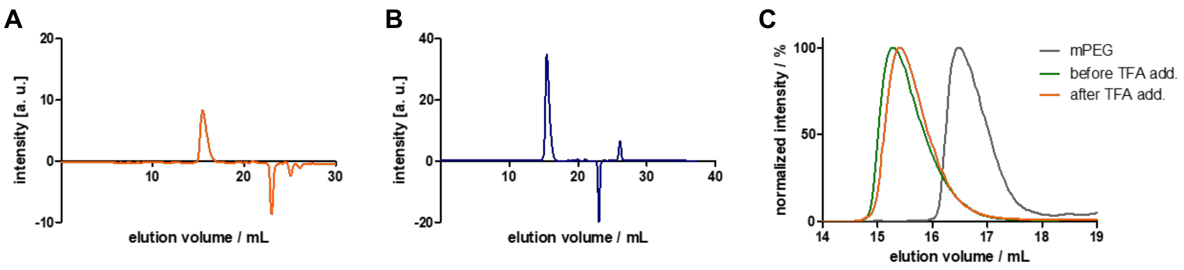

Figure S45: THF-GPC analysis of poly(ethylene glycol)$_{113}$-*b*-poly(MTC-OBn)$_m$ after TFA treatment. **A**: RI-traces and **B**: UV-traces of the received block copolymer. **C**: Section of the overlayed normalized RI-signal of the staring material (green) and the received product (orange) next to mPEG$_{113}$-OH (grey).

Backbone degradation in deuterated PBS

Side group degraded poly(ethylene glycol)$_{113}$-*b*-poly(MTC-OH)$_{19}$ and non-responsive poly(ethylene glycol)$_{113}$-*b*-poly(MTC-OBn)$_{21}$ were dissolved in 0.7 mL acetone at a polymer concentration of 7.14 mg/mL. DMSO (1.00 µL) was added as internal standard. The acetone solutions were mixed with 0.9 mL D_2O in tared vials which were stored uncapped in the fume hood overnight. On the next day, evaporated D_2O was refilled and 0.1 mL deuterated 10xPBS was added to each vial. A 70 µL aliquot of both samples was used for DLS measurement. The remaining solutions were immediately analyzed by ^{1}H NMR spectroscopy. Between each measurement the samples were stored at 37 °C. After 9 d of

repeated ^{1}H NMR measurements, the solutions were lyophilized, dissolved in DCM, filtered and then precipitated in ice-cold diethyl ether. The precipitates were isolated by centrifugation (5000 rpm, 20 °C, 20 min) and freeze dried from benzene (instead of benzene, anhydrous toluene can also be applied for removing traces of water azeotropically[3]). Finally, both samples were again characterized by ^{1}H NMR spectroscopy (in DMSO-d$_6$) and GPC analysis.

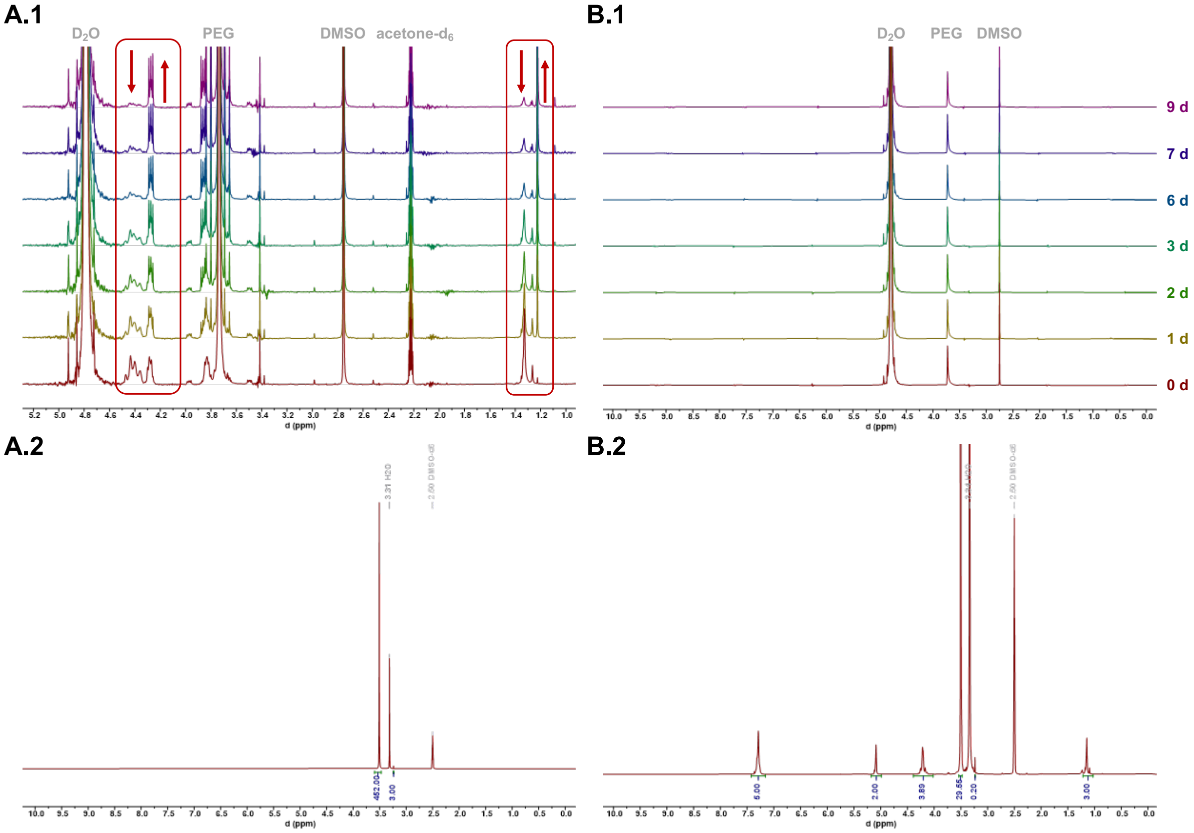

Figure S46: Backbone degradation study of hydrophilized poly(ethylene glycol)$_{113}$-*b*-poly(MTC-OEtOH)$_{19}$ (**A**) and micellar poly(ethylene glycol)$_{113}$-*b*-poly(MTC-OBn)$_{21}$ recorded by ^{1}H NMR spectroscopy in D_2O (**B**). Over 9 d, the polycarbonate backbone signals of the hydrophilized block copolymer decline and small molecule fragment signals appear that correspond to the hydrolyzed carbonate backbone (note that the ethylene glycol ester remains intact, **A.1**). In contrast, no new signals can be found evolving over time for polymeric micelles except for the hydrophilic mPEG corona (**B.1**). After isolation of both polymers the ^{1}H NMR spectrum in DMSO-d$_6$ reveals only PEG signals for the backbone degraded polymer (**A.2**) while the non-degraded polymer provided all polycarbonate signals with backbone (**B.1**).

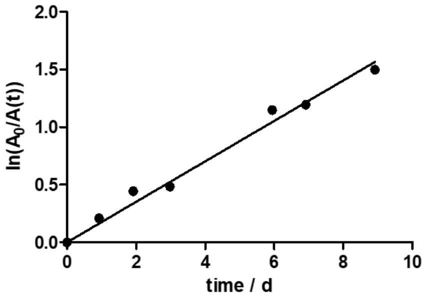

$$k = 0.176$$

$$t_{1/2} = \frac{\ln(2)}{k} = \frac{\ln(2)}{0.176} = 3.94 \text{ d}$$

Figure S47: Poly(carbonate) backbone degradation kinetics of poly(ethylene glycol)$_{113}$-*b*-poly(MTC-OEtOH)$_{19}$ recorded by ^{1}H NMR spectroscopy in D_2O. The exponential decay constant *k* is derived from the slop of the logarithmic plot of relative ^{1}H NMR integrals against time.

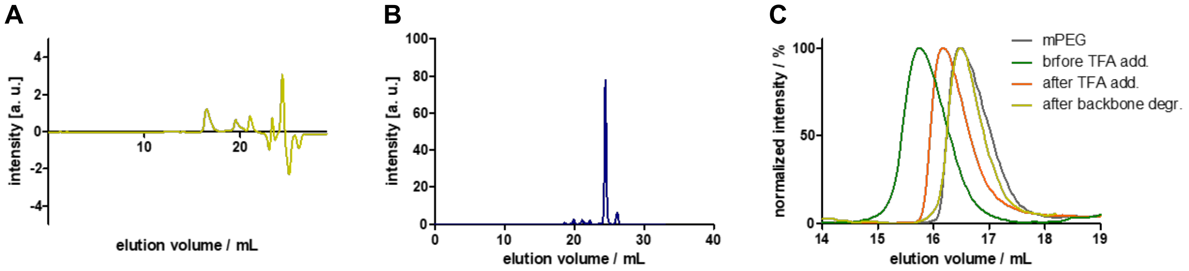

Figure S48: THF-GPC analysis of the hydrophilized block copolymer after backbone degradation. **A**: RI-traces and **B**: UV-traces of the carbonate backbone degraded polymer; since no aromatic groups are present anymore, no polymer UV signal can be found. **C**: Section of the overlayed normalized RI-signal of the staring material (green), the hydrophilized intermediate (orange) and the backbone degraded product (yellow) next to mPEG$_{113}$-OH (grey). It can be seen that the whole polycarbonate block degraded and only the mPEG$_{113}$-OH signal remains after poly(carbonate) backbone degradation.

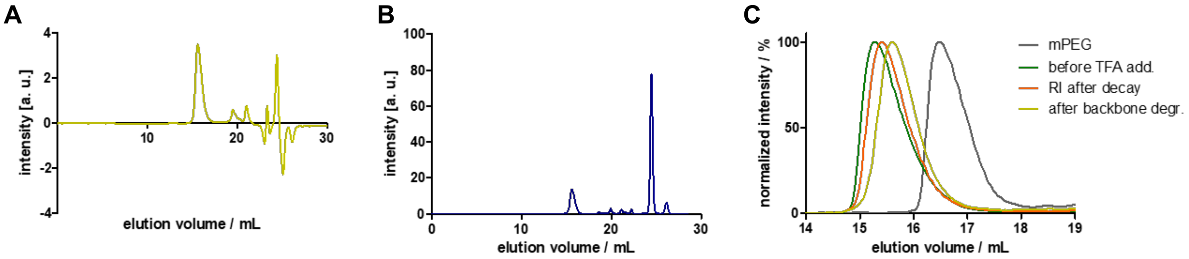

Figure S49: THF-GPC analysis of poly(ethylene glycol)$_{113}$-*b*-poly(MTC-OBn)$_{21}$ after the backbone degradation study. **A**: RI-traces and **B**: UV-traces of the received block copolymer. **C**: Section of the overlayed normalized RI-signal of the staring material (green) the received polymer after TFA treatment (orange) and after the poly(carbonate) backbone degradation study (yellow) next to mPEG$_{113}$-OH (grey).

THERMAL STABILITY OF FORMULATED BENZYL KETAL MICELLES IN PBS

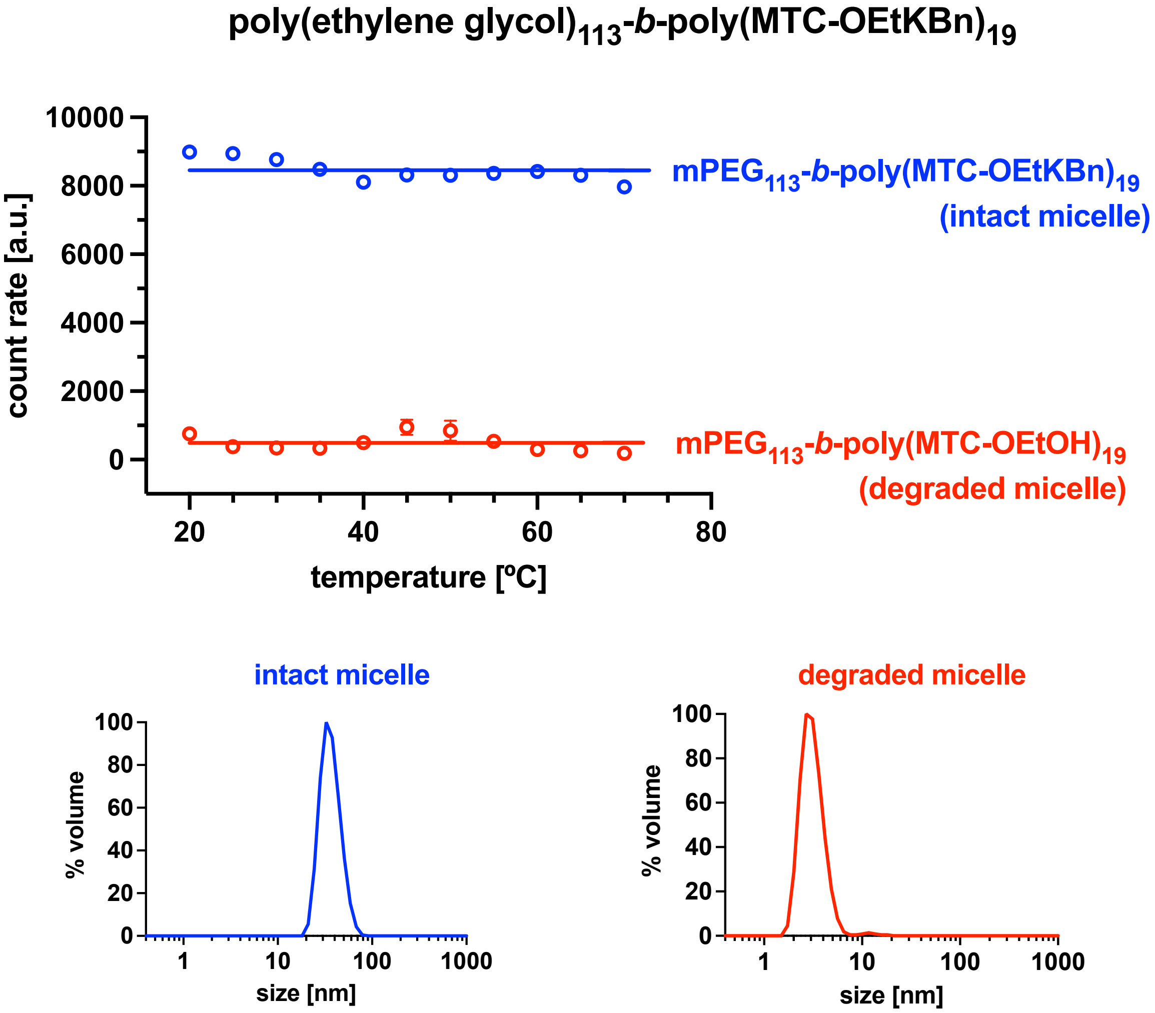

Figure S50: Thermal stability of formulated poly(ethylene glycol)$_{113}$-*b*-poly(MTC-OEtKBn)$_{19}$ block copolymer micelles in PBS and their degraded fully water-soluble block copolymer formulated poly(ethylene glycol)$_{113}$-*b*-poly(MTC-OEtOH)$_{19}$ (after ketal hydrolysis). The light scattering count rate of the samples at 1 mg/mL in PBS was monitored at increasing temperatures while no difference in scattering intensities or related size distributions could be found, confirming also the aqueous stability of the formulated benzyl ketal block copolymer micelles at physiological pH.

CRITICAL MICELLE CONCENTRATION

To determine the critical micelle concentration (CMC) of poly(ethylene glycol)$_{113}$-*b*-poly(MTC-OEtKBn)$_{19}$ and poly(ethylene glycol)$_{113}$-*b*-poly(MTC-OBn)$_{21}$, both polymers were formulated in PBS that contained 0.6 µM pyrene. For this purpose, the two polymers were dissolved in acetone (2.0 mL) at a polymer concentration of 4.0 mg/mL. The acetone solutions were mixed with 2.0 mL pyrene containing PBS (0.6 µM). The uncapped vials were stored in the fume hood overnight allowing the acetone to evaporate. Afterwards, evaporated water was refilled, and dilution series of the polymeric micelles were prepared (4.0 – 0.00004 mg/mL) in PBS that also contained 0.6 µM pyrene. 200 µL of each concentration were added to a black 96 well plate (Thermoscientific Nucleon™ Delta Surface) in triplicates and the pyrene fluorescence was measured using a TECAN Spark® plate reader (λ_{ex} = 333 nm, exbw = 5.0 nm; λ_{em} = 360–460 nm embw = 5.0 nm). The ratio of I_3 (384 nm) and I_1 (373 nm) fluorescence bands was plotted against the decadic logarithm of the polymer concentration, and linear regimes were found for small, medium and large polymer concentrations. The intersection of the linear regression line of the small concentration regime and the medium concentration regime was calculated, yielding the CMC. Afterwards, each well was treated with 10vol% HCl (1 M) and the measurement was repeated.

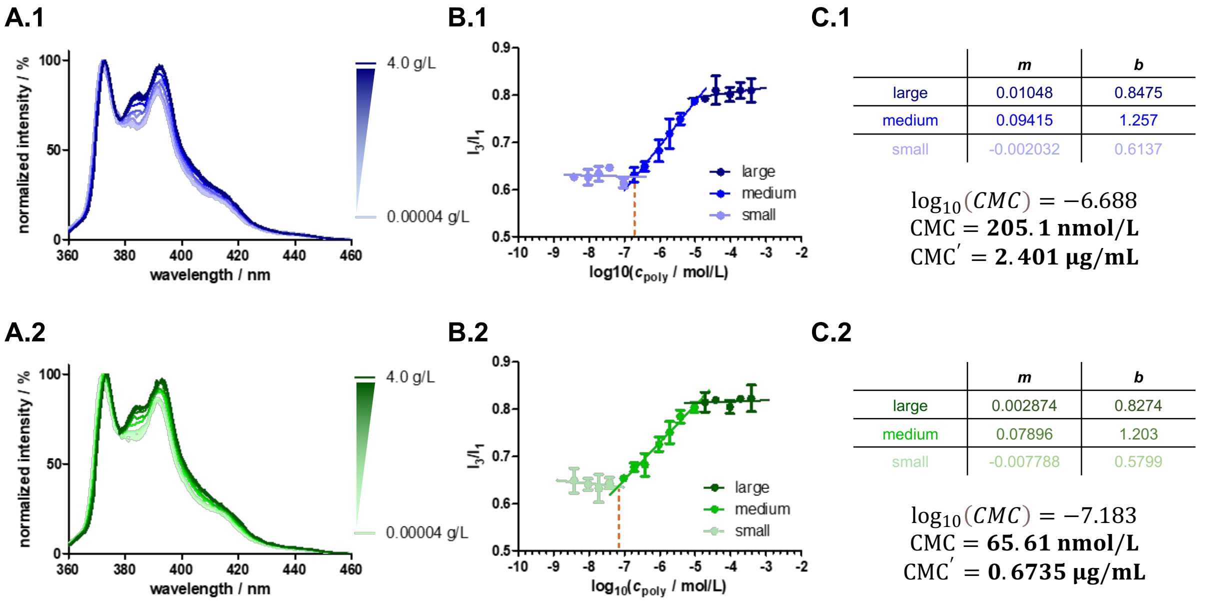

Figure S51: Critical micelle concentration (CMC) determination. **A**: Normalized pyrene fluorescence emission spectra of acid-responsive polymeric micelles (**A.1**) and non-responsive polymeric micelles (**A.2**) in a carrier concentration range of 4.0–0.00004 mg/mL. **B**: The I_3/I_1 ratio plotted against the $\log_{10}$ of the polymer concentration gives three linear regimes (small, medium, large) for the responsive system (**B.1**) and the non-responsive system (**B.2**). The slope *m* and the y-axis intersection *b* of the linear fit functions are summarized in the tables **C.1** (acid-responsive) and **C.2** (non-responsive). Below the tables, the calculations of the intersection of the small and medium regime are shown, giving the respective CMC.

TRANSMISSION ELECTRON MICROSCOPY (TEM) IMAGING

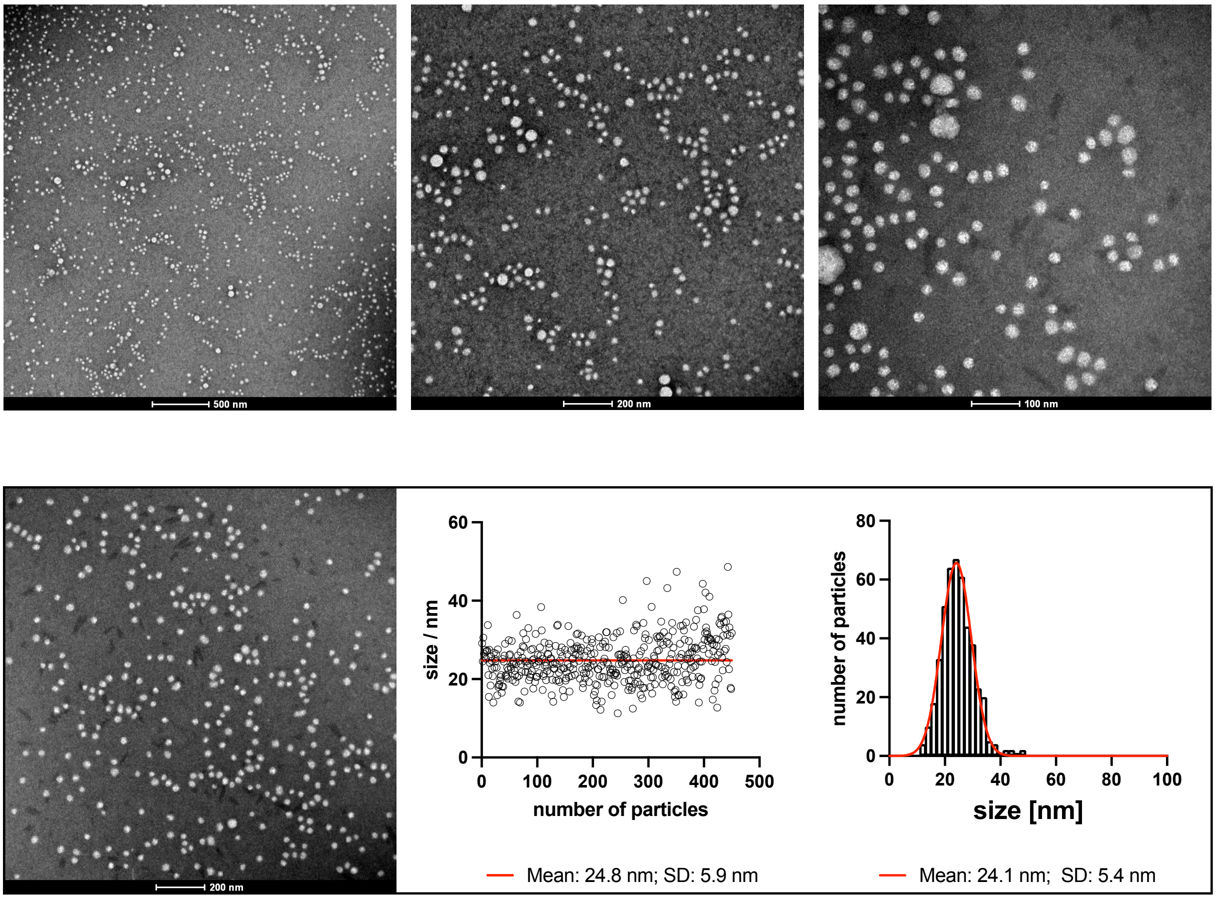

Figure S52: Selection of TEM pictures of polymeric micelles, formulated from mPEG$_{113}$-b-p(MTC-OEtKBn)$_{10}$. The diameters of all particles from the lower image were counted and their average size evaluated by arithmetic mean or histogram analysis.

COMPARISON OF MICELLAR SELF ASSEMBLIES

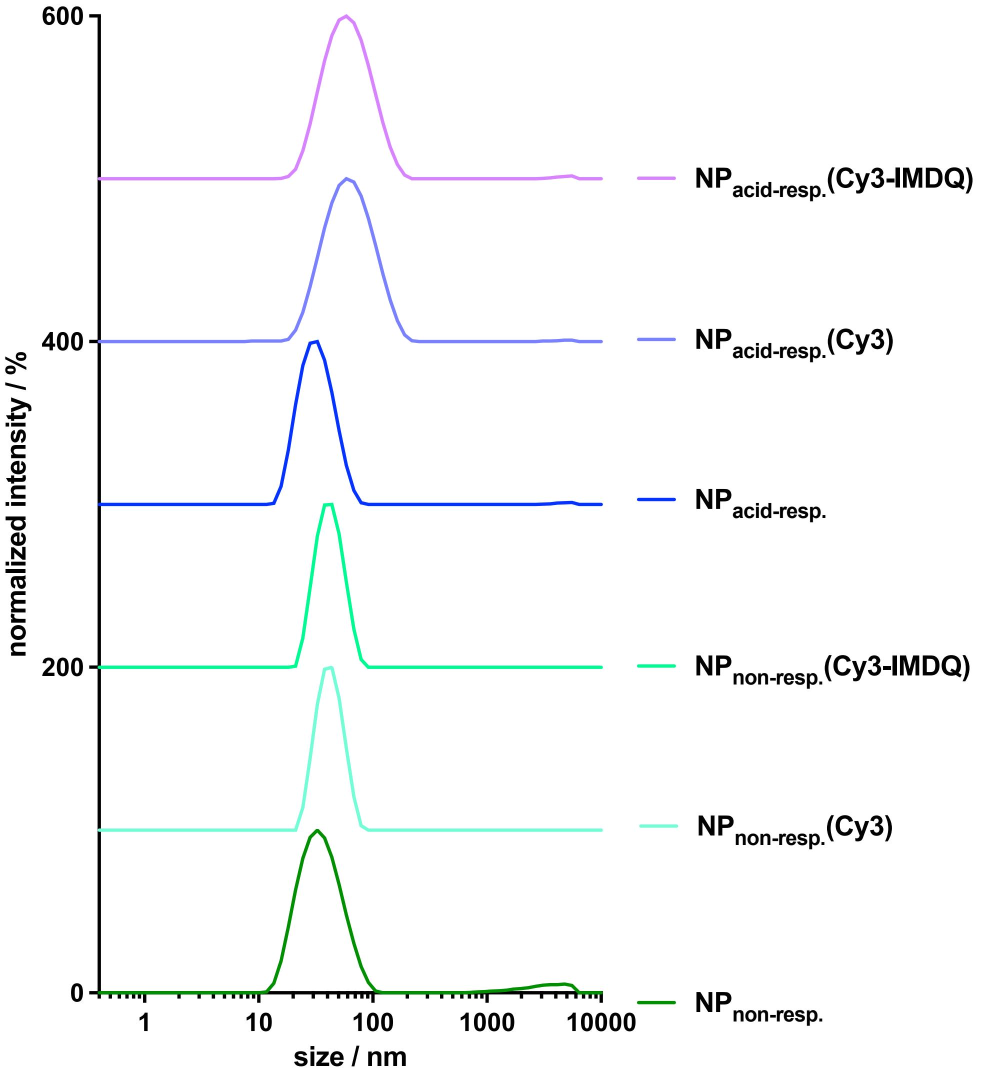

Figure S53: Representative DLS measurements of non-labeled as well as Cy3 alone and Cy3+IMDQ-labeled nanoparticles showing the normalized intensity plot for each sample.

IN VITRO CELL EXPERIMENTS

Cell Uptake by Macrophages

Sample Preparation

Cell uptake of acid-responsive and non-responsive polymeric micelles with and without IMDQ loading was evaluated by flow cytometry (FC) and confocal fluorescence microscopy (CFM). For this purpose, four micelle samples were formulated mixing unlabeled, Cy3-labeled and IMDQ-labeled block copolymers. In all cases, the overall polymer concentration was 1.0 mg/mL and 10 µg/mL dye labeling was targeted. For IMDQ-loaded polymeric micelles a drug loading of 25 µmol/L was targeted. After mixing the respective block copolymers, they were dissolved in acetone and an equivalent volume sterile PBS was added. After evaporation of acetone overnight, additionally evaporated water was quantified by weighing the vials and refilled. After filtration through sterile hydrophilized PTFE syringe filters (0.20 µm pore size, Macherey-Nagel) an aliquot of each sample was analyzed by DLS (Figure S54). Dye and drug loading was determined by UV/vis spectroscopy (Figure S55). Finally, dilution series were prepared with carrier concentrations of 1.0 mg/mL, 0.25 mg/mL and 0.05 mg/mL.

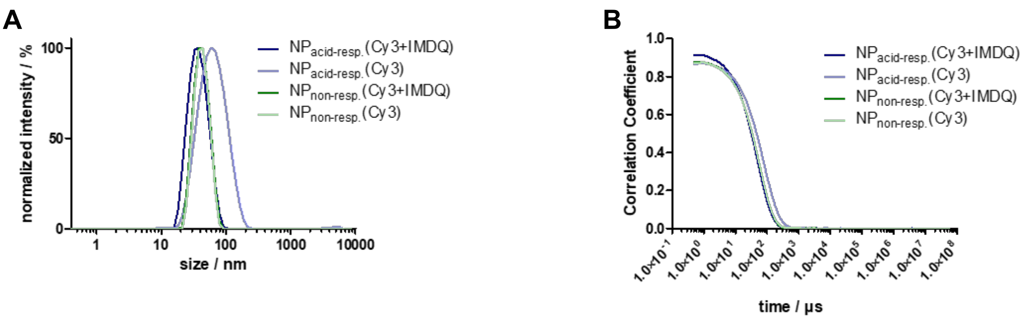

Figure S54: DLS results of Cy3 and Cy3+IMDQ-labeled nanoparticles showing the normalized intensity plot (**A**) and the correlogram (**B**) for each sample.

A.1

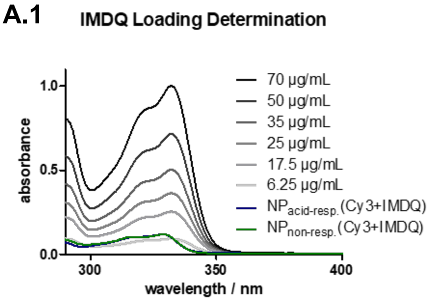

A.2

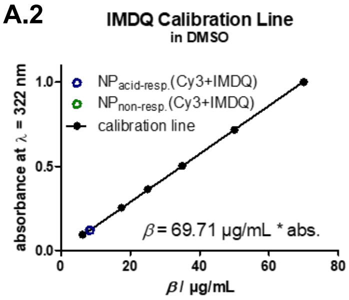

A.3

	Abs.	Loading / µmol/L	Efficiency
**NP$_{acid\text{-}resp.}$ (Cy3+IMDQ)**	0.1196	23.3	93%
**NP$_{non\text{-}resp.}$ (Cy3+IMDQ)**	0.1193	23.2	93%

B.1

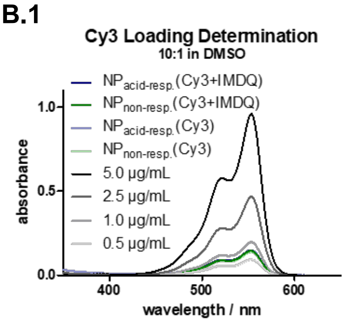

B.2

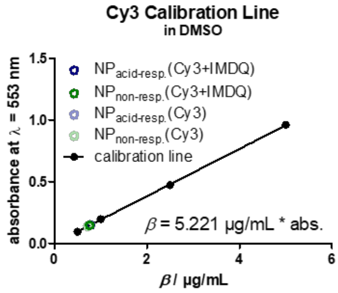

B.3

	Abs.	Loading / µg/mL	Efficiency
**NP$_{acid\text{-}resp.}$ (Cy3)**	0.1498	7.82	78%
**NP$_{non\text{-}resp.}$ (Cy3)**	0.1396	7.29	73%
**NP$_{acid\text{-}resp.}$ (Cy3+IMDQ)**	0.1485	7.75	78%
**NP$_{non\text{-}resp.}$ (Cy3+IMDQ)**	0.1465	7.65	77%

Figure S55: Drug (**A**) and dye (**B**) loading determination by UV/vis spectroscopy. **A**: The absorption spectra of the interesting regime are shown for IMDQ (**A.1**) and Cy3 (**B.1**) next to the absorption spectra of calibration measurements. **B**: The corresponding calibration lines are shown for IMDQ (**A.2**) and Cy3 (**B.2**) together with the respective sample absorptions. **C**: In the tables the values of these absorptions and the resulting loadings and loading efficiencies are summarized for IMDQ loading (**A.3**) and Cy3 labeling (**B.3**) respectively.

Flow Cytometry (FC)

A 24-well plate was seeded with 900 µL of a RAW-Blue™ macrophage cell suspension per well ($0.28\cdot10^6$ cells/mL, 250,000 cells/well). The plate was stored in the incubator for 24 h (37 °C, 5% CO_2,). After the cells adhered to the well bottom 100 µL of the sterile formulated polymeric micelle solution were added resulting in final polymeric micelle concentrations of 100, 25 and 5 µg/mL. As reference, cells were treated with PBS. The cells were incubated for 16 h. After removing the cell culture medium, the cells were washed with 1 mL PBS per well. Subsequently, 500 µL of dissociation buffer (0.5 mM EDTA in PBS, pH = 7.4) were added to each well and the cells were incubated for 20 min. The detached cells were transferred into micro centrifuge tubes and stored on ice. The cells were separated from the dissociation buffer by centrifugation (10 min, 1,000 rpm, 5 °C) and resuspended in 200 µL PBS. Flow cytometry analysis was performed on a BD Accuri™ C6 plus flow cytometer. Each measurement was run until 30,000 cells were counted. Data were processed using FlowJo software package (vX.0.7, FlowJo, LLC, OR, USA). Each sample was measured as triplicate (n = 3).

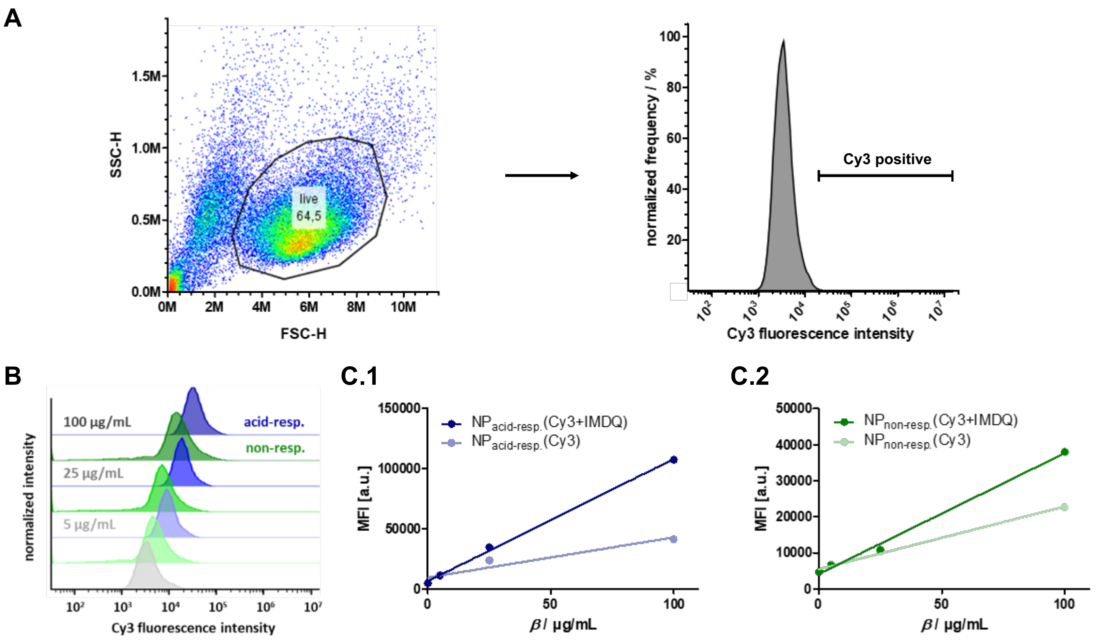

Figure S56: A: Flow cytometry gating strategy demonstrated on a PBS reference sample. Cy3 fluorescence was recorded in the PE channel (λ_{ex} = 488 nm, detection filter 585/40 nm). **B**: Cy3 fluorescence intensity of cells incubated with Cy3 only-labeled nanoparticles at different concentrations. **C**: Linear dependency of the mean fluorescence intensity on the acid-responsive ketal carrier amount (**C.1**) and the non-responsive benzyl carrier amount (**C.2**) with and without IMDQ co-delivery.

Confocal Fluorescence Microscopy (CFM)

RAW-Blue™ macrophages were seeded in an eight-well polystyrene microscopy chamber supplied by SARSTEDT (0.28·10^6 cells/mL, 180 µL, 50,000 cells/well). The eight-well plate was stored in the incubator (37 °C, 5% CO_2,) for 24 h. After the cells adhered to the well bottom 20 µL of the highest concentrated polymeric micelle solutions from the FC experiment were added to each well resulting in a final polymeric micelle concentration of 100 µg/mL. PBS was used as reference. After another 24 h incubation the cell culture medium was removed, and cells were washed with PBS (3 x 200 µl). Subsequently, 200 µl of 4% paraformaldehyde were added and fixed for 15 min (37 °C, 5% CO_2,). Cells were washed again with PBS (3 x 200 µl) and secured in DAPI containing mounting medium (Immunoselect Antifading Mounting Medium DAPI by Dianova). Images were taken using a 20x water immersion objective on a Leica SP8 confocal microscope (Wetzlar, Germany). Leica's software LAS X was used for image evaluation.

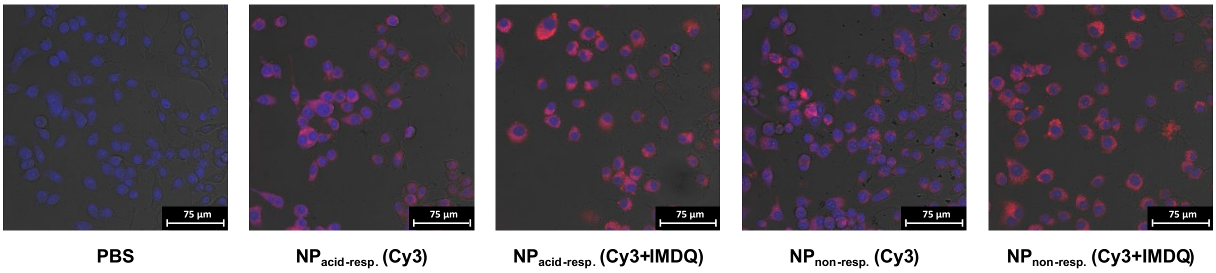

Figure S57: Additional confocal fluorescence microscopy images of Cy3- and Cy3+IMDQ-labeled nanoparticle uptake as well as an image of the PBS reference.

QUANTI-Blue™ and MTT Assay

For the QUANTI-Blue™ and MTT assay, IMDQ-loaded acid-responsive and non-responsive nanoparticle samples were formulated. In addition, the carrier materials without IMDQ-loading were tested as well. For IMDQ-labeled samples a drug loading of 400 µmol/L was targeted, and samples were formulated at a carrier concentration of 1.0 mg/mL. The respective block copolymers were dissolved in acetone and an equal volume sterile PBS was added. After evaporation of acetone overnight, additionally evaporated water was quantified by weighing the vials and refilled. Subsequently, all samples were filtered through hydrophilized PTFE syringe filters (0.20 µm pore size, Macherey-Nagel) and analyzed by UV/vis spectroscopy and DLS (Figure S58). From IMDQ-loaded samples dilution series were prepared in an IMDQ concentration range of 200–0.40 µmol/L. The unlabeled samples were diluted in analog way. As references a dilution series of soluble IMDQ was prepared in a concentration range of 20–0.04 µmol/L.

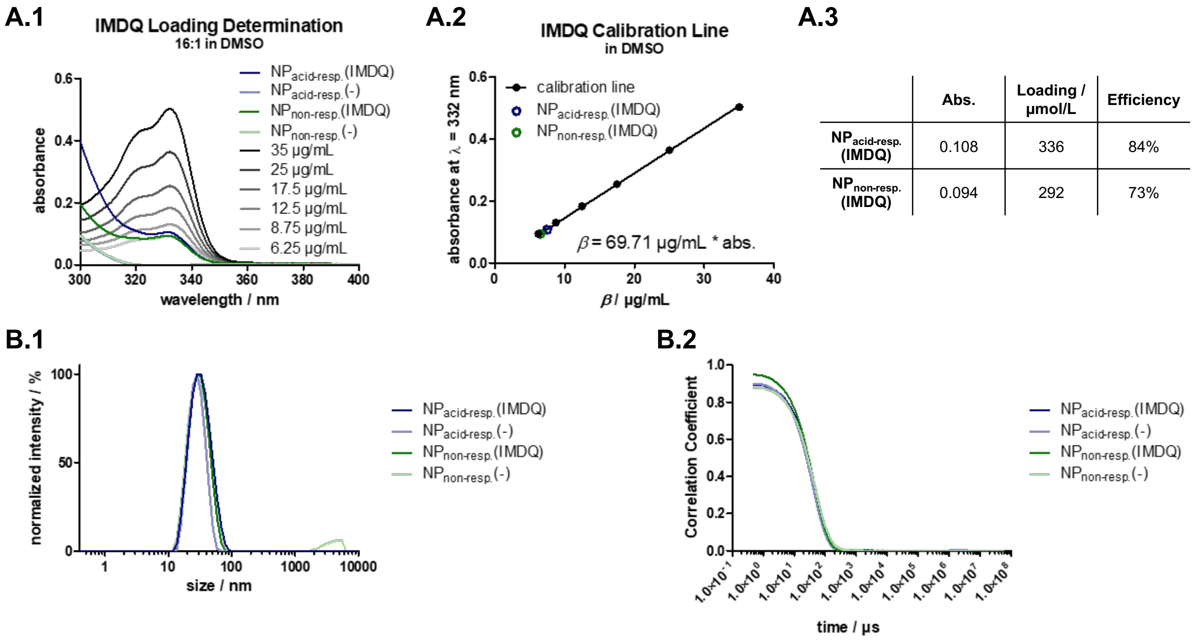

	Abs.	Loading / µmol/L	Efficiency
$NP_{acid\text{-}resp.}$ (IMDQ)	0.108	336	84%
$NP_{non\text{-}resp.}$ (IMDQ)	0.094	292	73%

Figure S58: Characterization of samples for QUANTI-Blue™ and MTT assay. **A**: IMDQ loading determination *via* UV/vis spectroscopy. The spectra are shown in **A.1** together with the spectra for the calibration measurements. The corresponding calibration line is shown in **A.2** together with the respective sample absorptions maxima. **A.3** summarizes the values of these absorptions and the resulting loadings and loading efficiencies. **B**: DLS analysis showing the normalized intensity plots (**B.1**) and the correlograms (**B.2**).

A RAW-Dual™ cell suspension ($c = 0.5 \cdot 10^6$ cells/mL) was seeded into 96-well plats (180 µL per well, 90,000 cells/well). After overnight incubation (37 °C, 5% CO_2), 20 µL of each concentration of the previously prepared samples were added to the wells as quartets ($n = 4$). As references PBS, IMDQ (20 µmol/L) and DMSO were added to each 96-well plate as well ($n = 4$). The treated cells were incubated for 24 h. Afterwards, 150 µL QUANTI-Blue™ solution were added to each well of new 96-well plates. Subsequently, 50 µL of cell supernatant was added to the QUANTI-Blue™ solution and

incubated (37 °C, 5% CO_2) for 1 h. The QUANTI-Blue™ readout was performed at a wavelength of 620 nm using a TECAN Spark® plate reader.

For the MTT assay the remaining cells were treated with 30 µL MTT-solution (2 mg/mL MTT). After 1 h incubation, SDS-solution was added (10wt% SDS, 0.01 M HCl) and the cells were incubated for 24 h (37 °C, 5% CO_2). Afterwards, the MTT readout was performed on a TECAN Spark® plate reader at an absorption wavelength of 570 nm.

Cell Uptake by Bone Marrow Derived Dendritic Cells (BMDC) and Immune Cell Maturation

Cell Uptake

To investigate the uptake by BDMCs, Cy5- and Cy5+IMDQ-labeled nanoparticles were sterile formulated from acid-responsive and non-responsive block copolymers. The targeted Cy5 labeling was 200 µg/mL and for IMDQ containing samples it was aimed for a drug loading of 1.0 mmol/L. The overall carrier concentration was 10 mg/mL. After mixing all ingredients, acetone and sterile PBS was added to the samples, that were stored uncapped in a flow hood overnight. Next day, evaporated water was refilled, and the samples were filtered through hydrophilized PTFE syringe filters (0.20 µm pore size, Macherey-Nagel). An aliquot of each sample was used for dye and drug loading determination by UV/vis spectroscopy.

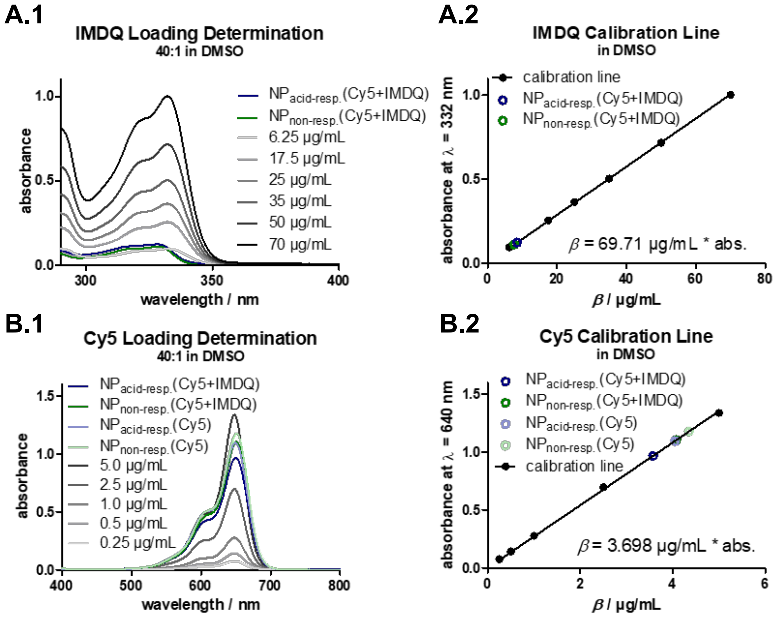

A.3

	Abs.	Loading / µmol/L	Efficiency
$NP_{acid\text{-}resp.}$ (Cy5+IMDQ)	0.122	949.0	95%
$NP_{non\text{-}resp.}$ (Cy5+IMDQ)	0.110	855.6	86%

B.3

	Abs.	Loading / µg/mL	Efficiency
$NP_{acid\text{-}resp.}$ (Cy5+IMDQ)	0.9680	143.2	72%
$NP_{non\text{-}resp.}$ (Cy5+IMDQ)	1.1039	163.3	82%
$NP_{acid\text{-}resp.}$ (Cy5)	1.0955	162.0	81%
$NP_{non\text{-}resp.}$ (Cy5)	1.1768	174.1	87%

Figure S59: Drug (**A**) and dye (**B**) loading determination by UV/vis spectroscopy. The absorption spectra are shown for IMDQ labeling (**A.1**) and Cy5 labeling (**B.1**) next to the respective calibration measurements. The corresponding calibration lines are shown for IMDQ (**A.2**) and Cy5 (**B.2**) together with the respective sample absorption maxima. In the tables the values of the maximum absorptions and the resulting loadings and loading efficiencies are summarized for IMDQ loading (**A.3**) and Cy5 loading (**B.3**) respectively.

Bone marrow cells were isolated from femurs and tibiae of C57BL/6 mice and resuspended in IMDM-based culture medium (5% FBS, 2 mM L-glutamine, 100 IU/ml penicillin, 100 µg/ml streptomycin and 50 µM β-mercaptoethanol; all from SigmaAldrich, Deisenhofen, Germany), supplemented with 10 ng/ml recombinant murine GM-CSF (R&D Systems, Wiesbaden, Germany) to obtain inflammatory BMDCs. The cells were seeded in 12-well plates ($1\cdot10^5$ cells/mL) and the cell culture medium was replenished on days 3 and 6. On days 7–8, the samples were added to the differentiated cells resulting in final IMDQ concentrations of 4 µmol/L. References without IMDQ were added in analogy to the loaded samples. The following day, cells were harvested and analyzed by flow cytometry.

<u>BMDC Maturation</u>

To investigate the maturation of BDMCs upon IMDQ delivery, TAMRA- and TAMRA+IMDQ-labeled nanoparticles were sterile formulated from acid-responsive and non-responsive block copolymers (TAMRA was used instead of Cy5 to prevent fluorescence spillover during flow cytometric assessment). The targeted TAMRA labeling was 50 µg/mL and for IMDQ containing samples it was aimed for a drug loading of 0.5 mmol/L. The overall carrier concentration was 10 mg/mL. After mixing all ingredients, acetone and sterile PBS was added to the samples, that were stored uncapped in a flow hood overnight. Next day, evaporated water was refilled, and the samples were filtered through hydrophilized PTFE syringe filters (0.20 µm pore size, Macherey-Nagel). An aliquot of each sample was used for dye and drug loading determination by UV/vis spectroscopy. BMDCs were seeded as described earlier and samples were added resulting in final IMDQ concentration of 5 µmol/L. References without IMDQ were added in analogy to the loaded samples. The following day, cells were harvested and analyzed by flow cytometry.

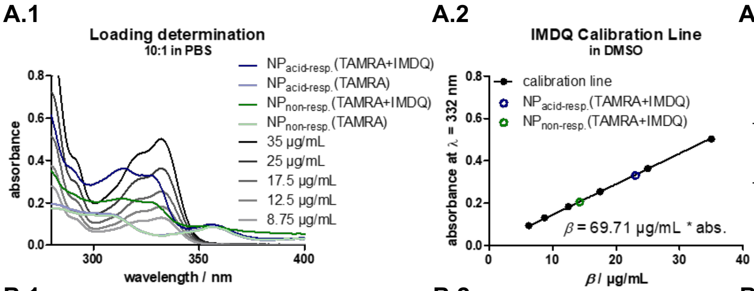

A.3

	Abs.	Loading / µg/mL	Loading / mmol/L	Efficiency
$NP_{acid\text{-}resp.}$ (TAMRA+ IMDQ)	0.267	186	0.52	104%
$NP_{non\text{-}resp.}$ (TAMRA+ IMDQ)	0.141	98.3	0.27	54%

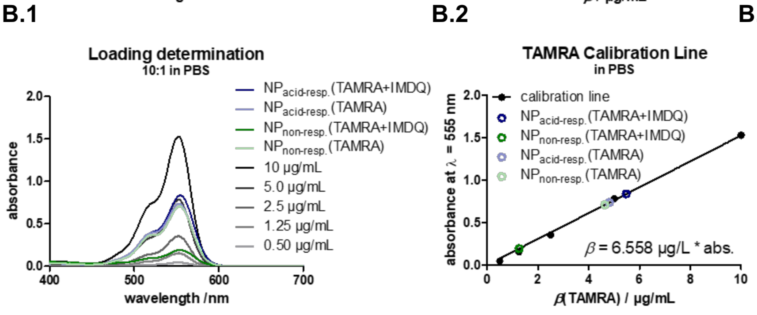

B.3

	Abs.	Loading / µg/mL	Efficiency
$NP_{acid\text{-}resp.}$ (TAMRA+IMDQ)	0.836	54.8	110%
$NP_{non\text{-}resp.}$ (TAMRA+IMDQ)	0.193	12.7	25%
$NP_{acid\text{-}resp.}$ (TAMRA)	0.735	48.2	96%
$NP_{non\text{-}resp.}$ (TAMRA)	0.708	46.4	93%

Figure S60: Drug (**A**) and dye (**B**) loading determination by UV/vis spectroscopy. The absorption spectra are shown for IMDQ labeling (**A.1**) and TAMRA labeling (**B.1**) next to the respective calibration measurements. The corresponding calibration lines are shown for IMDQ (**A.2**) and TAMRA (**B.2**) together with the respective sample absorption maxima. In the tables the values of the maximum absorptions and the resulting loadings and loading efficiencies are summarized for IMDQ loading (**A.3**) and TAMRA loading (**B.3**) respectively.

Cytokine Analysis Using Cytometric Bead Array

Cytokine secretion by the previous used cells were measured using a cytometric bead array (CBA; BD Biosciences, San Jose, CA), following the manufacturer's instructions. Bead populations with distinct fluorescence intensities were conjugated to cytokine-specific capture antibodies. Standard dilutions were prepared using recombinant cytokines. Samples were incubated with capture beads and then with PE-conjugated detection antibodies for 1 h (all at room temperature and protected from light). Flow cytometric analysis was performed, and the results were analyzed with FCAP Array Analysis Software v.1.0.1 (BD Biosciences).

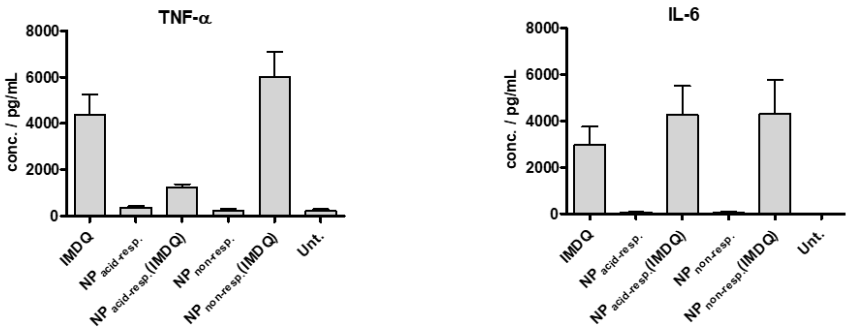

Figure S61: Cytokine secretion of BDMCs after incubation with nanoparticle samples and references.

FRET EXPERIMENTS

FRET Particle Unfolding Study

In order to investigate nanocarrier unfolding profiles using a FRET probe, Cy3-labeled, and Cy5-labeled block copolymers were co-formulated into polymeric micelles. The formulation was conducted with acid degradable and non-degradable block copolymers. In both cases, one part of Cy3-labeled block copolymer was mixed with one part of Cy5-labeled block copolymer and two parts non-labeled carrier material. PBS was added, giving an overall polymer concentration of 1.0 mg/mL. To exclude interference by direct acceptor dye excitation or fluorescence overlap of the two dyes, Cy3 and Cy5 only-labeled samples were also formulated with equal dye loadings and carrier concentrations. Afterwards, a black 96-well plate was filled with 180 µL buffer solutions (PBS: pH 7.4–6.5, acetate buffer: pH 5.5–3.6) per well ($n = 3$). All buffer solutions were mixed with 20 µL sample solutions leading to a final particle concentration of 0.1 mg/mL. To investigate the particle degradation, fluorescence measurements were repeatedly conducted over a period of 38 d using a TECAN Spark® plate reader (λ_{ex} = 500 nm, exbw = 5 nm, embw = 5 nm). The spectra at t_1 = 0.05 h and t_6 = 24 h are shown in Figure S62. For all other time points, only the fluorescence maxima of the two dyes were measured.

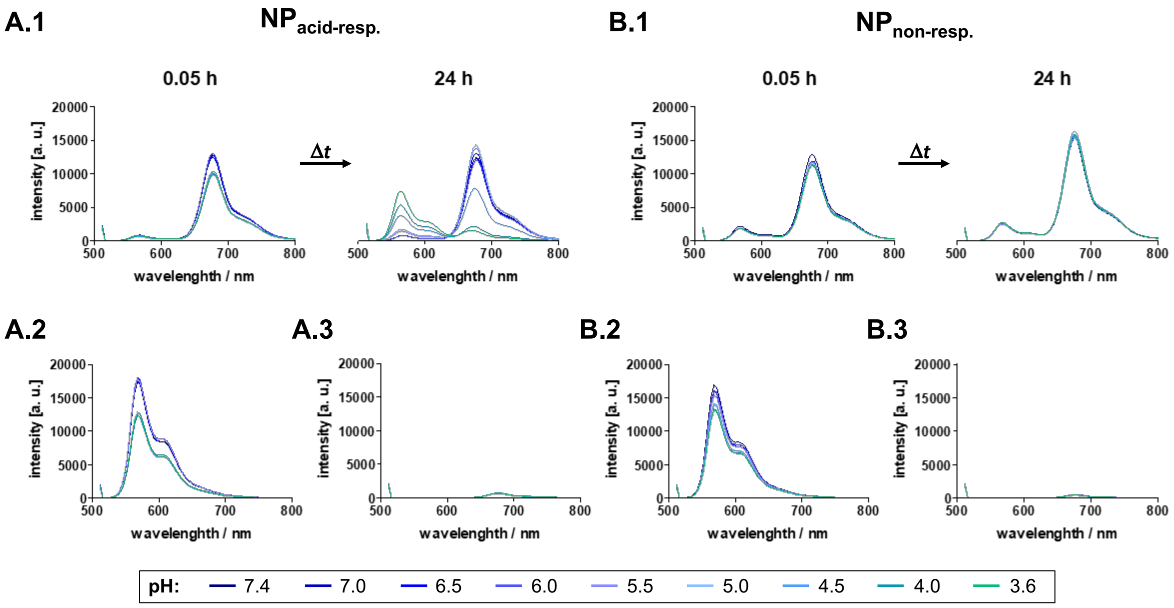

Figure S62: Fluorescence measurements of dye-labeled acid-responsive (**A**) and non-responsive (**B**) micellar nanoparticles. The FRET pair-labeled particles give an intense FRET signal after mixing. After 24 h, the FRET signal of acid-responsive particles in acidic environments declined (**A.1**), while for the non-responsive system no change was observed (**B.1**). The Cy3 only-labeled particles exhibited a Cy3 characteristic fluorescence (**A.2**, **B.2**) that can be distinguished from the FRET signal. Cy5 only-labeled particles showed no significant fluorescence (**A.3**, **B.3**) excluding that the FRET fluorescence is a result of direct Cy5 excitation.

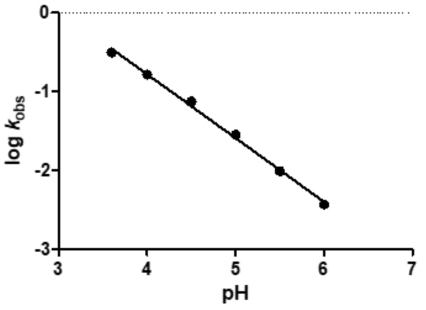

$$k_{obs.} = k_0 + k_H[H^+]$$

$$m = -0.811 \pm 0.02$$

Figure S63: Logarithm of the observed degradation rate constant $\boldsymbol{k_{obs}}$ determined by the fluorescence measurements plotted against the pH value. The observed rate constant $\boldsymbol{k_{obs.}}$ is the linear combination of the first order rate constant for non-catalyzed degradation in water $\boldsymbol{k_0}$ and the second order rate constants for its degradation catalyzed by protons $\boldsymbol{k_H}$. As the slope $\boldsymbol{m}$ of the log($k_{obs.}$)–pH-profile approaches -1 (m = -0.811 ± 0.02), the observed ketal hydrolysis follows a specific single proton catalyzed hydrolysis mechanism.[1]

Single Chain Exchange Study

To test the exchange of individual polymer chains between different micelles Cy3 and Cy5 single dye-labeled micelles were formulated in PBS at a carrier concentration of 1.0 mg/mL separately. For this purpose, two parts of dye-labeled material were mixed with one part of unlabeled material. As reference, FRET pair-labeled micelles were formulated by mixing Cy3, Cy5 and unlabeled polymer in a 1:1:1 ratio. All formulations were prepared with acid-responsive and non-responsive block copolymer material. The single dye-labeled polymeric micelles were mixed in a transparent 96-well plate using 100 µL of each formulation. As reference 200 µL of the FRET pair-labeled micelles were used. Immediately after mixing emission spectra were recorded. The measurements were repeatedly conducted over a period of

one week using a TECAN Spark® plate reader (λ_{ex} = 500 nm, exbw = 5 nm, embw = 5 nm). In between the measurements, the samples were stored at 37 °C.

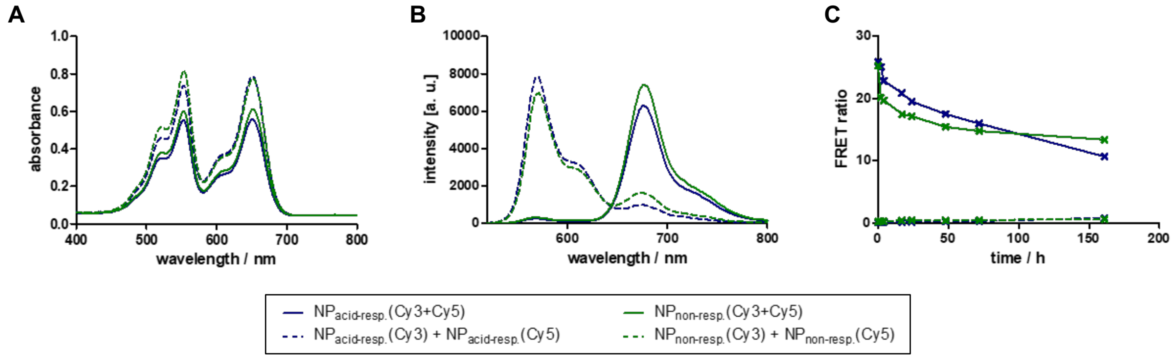

Figure S64: Single chain exchange study of acid-responsive nanoparticles and non-responsive nanoparticles. **A**: Absorption spectra of Cy3 and Cy5 single dye-labeled micelles in PBS mixed after formulation and particles with both dyes co-formulated. **B**: Fluorescence spectra (λex = 500 nm) exhibiting FRET signals for co-formulated dyes and Cy3 emission for mixed single dye-labeled nanoparticles, indicating no polymer chain exchange. **C**: Time dependent FRET ratio of FRET dye-labeled and mixed single dye-labeled polymeric micelles.

FRET Stability Study in Blood Plasma

FRET pair-labeled acid-responsive and non-responsive polymeric micelles from Cy3 and Cy5 dye-labeled co-formulated micelles were used to test micellar stability in human blood plasma. For this purpose, 20 µL of the particle samples were mixed with 180 µL plasma in a transparent 96-well plate. The same procedure was also performed with plasma containing 10vol% HCl (1 M). Emission spectra were recorded, and fluorescence measurements were repeatedly conducted over a period of one week using a TECAN Spark® plate reader (λ_{ex} = 500 nm, exbw = 5 nm, embw = 5 nm). In between the measurements, the samples were stored at 37 °C.

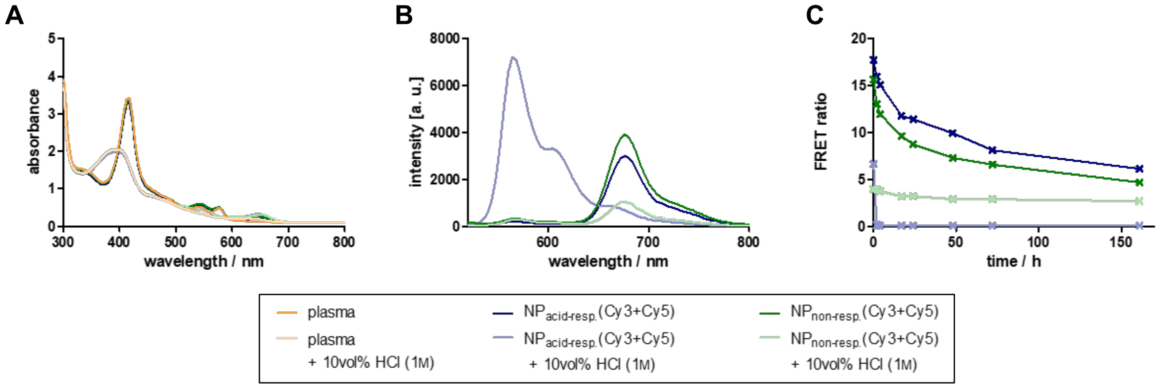

Figure S65: Particle stability study in human blood plasma testing the stability of acid-responsive and non-responsive nanoparticles. **A**: Absorption spectrum of plasma samples incubated with FRET pair-labeled polymeric micelles with and without the 10vol% HCl (1M) added to the plasma. **B**: Fluorescence spectra (λex = 500 nm) exhibiting a FRET signal for both particles under physiological conditions. In the acidic environment a reduced intensity was observed for the non-responsive nanoparticles but no Cy3 emission, while a vast loss of the FRET signal was found for acid-responsive nanoparticles resulting in intense Cy3 fluorescence. **C**: Time dependency of the FRET ratio for both particles under physiological conditions and in the acidic environment. Under physiological conditions both particles reveal a declining but distinctive FRET ratio. Non-responsive particles show a reduced but constant FRET ratio in the acidic environment. In contrast the FRET ratio of acid-responsive nanoparticles quickly approaches zero, indicating immediate particle unfolding.

Flow Cytometry derived FRET-based Intracellular Unfolding

<u>Pre-Study</u>

To evaluate the suitability of the FRET signal as valuable probe for recording micellar unfolding inside cells *via* flow cytometry, a pre-study was conducted with FRET pair-labeled polymeric micelles and the respective single dye-labeled polymeric micelles. For this purpose, polymeric micelles were formulated from acid-responsive and the non-responsive block copolymers. For FRET pair-labeled particles, one part Cy3-labeled block copolymer was mixed with one part Cy5 labeled block copolymer and two parts of unlabeled block copolymer. For the single dye-labeled particles, one part of the respective dye-labeled block copolymer was mixed with three parts unlabeled block copolymer. After the addition of sterile PBS, the final particle concentration was 1.0 mg/mL. All samples were filtered through hydrophilized PTFE syringe filters (0.20 µm pore size, Macherey-Nagel). Dye loading was evaluated by UV/vis spectroscopy and fluorescence spectroscopy using a TECAN Spark® plate reader (Figure S66A–B).

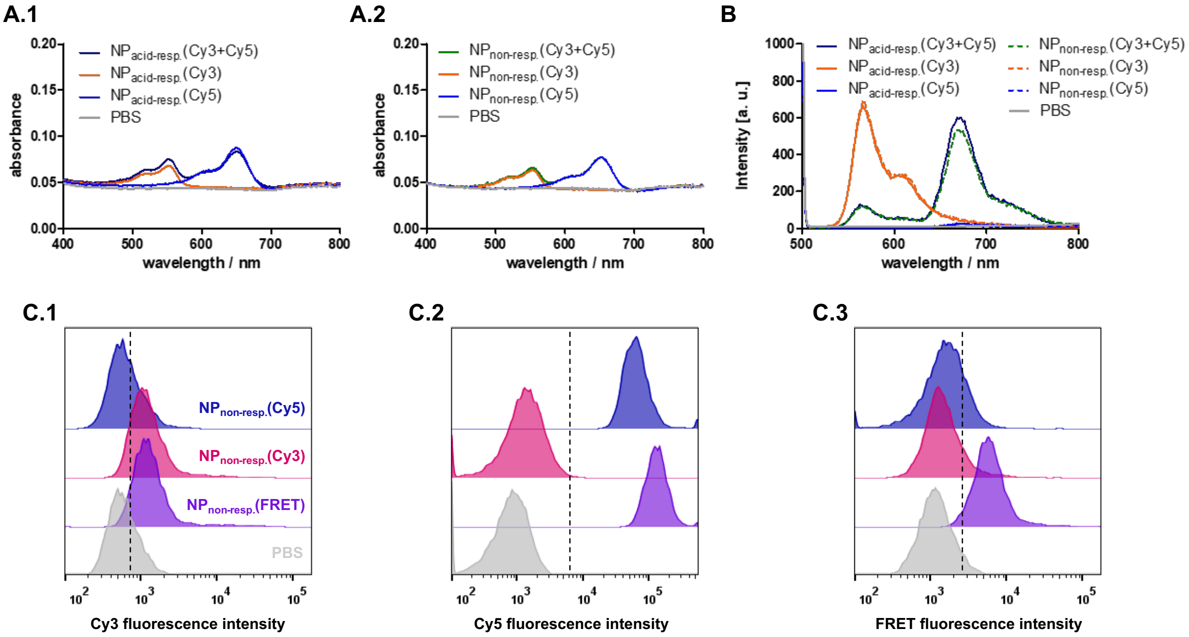

Figure S66: FRET evaluation of micellar integrity by flow cytometry in RAW-Blue™ macrophages. **A**: Dye loading evaluation by UV/vis spectroscopy of acid-responsive (**A.1**) and non-responsive nanoparticles (**A.2**). **B**: The dye labeling and fluorescence behavior at an excitation wavelength of 488 nm was evaluated for all samples by fluorescence spectroscopy. **C**: Mean fluorescence intensity distribution of RAW-Blue™ macrophages after uptake of non-responsive nanoparticles detected by flow cytometry. The different channels were used to detect Cy3 emission (**C.1**) Cy5 emission (**C.2**) and the FRET signal (**C.3**).

A 24-well plate was filled with 900 µL cell suspension per well ($0.28 \cdot 10^6$ cells/mL, 250,000 cells/well). The 24-well plate was stored in an incubator (37 °C, 5% CO_2,) for 24 h. After the cells adhered to the well bottom 100 µL of a sterile prepared polymeric micelle solution was added resulting in a final polymeric micelle concentration of 100 µg/mL. The cells were incubated for 24 h. After removing the

cell culture medium, the cells were washed with 1 mL PBS per well. Subsequently, 500 µL of dissociation buffer (0.5 mM EDTA in PBS, pH = 7.4) was added to each well and the cells were incubated for 20 min. The detached cells were transferred into micro centrifuge tubes and stored on ice. The cells were separated from the dissociation buffer by centrifugation (10 min, 1,000 rpm, 5 °C) and resuspended in 200 µL PBS. Flow cytometry analysis was performed using a BD Accuri™ C6 Plus flow cytometer. To monitor Cy3 fluorescence the FL2 channel was used (λ_{ex} = 488 nm, detection filter 585/40 nm). Cy5 fluorescence was analyzed using the FL4 channel (λ_{ex} = 640 nm, detection filter 675/25 nm). The FRET signal was detected using the FL3 channel (λ_{ex} = 488 nm, detection filter 670nm/LP). The measurements were run until 30,000 cells were counted. Each sample was conducted as triplicate (n = 3). Data were processed using FlowJo software package (vX.0.7, FlowJo, LLC, OR, USA).

Time Dependent FRET Analysis in Cells

For the time dependent FRET micelle unfolding study in macrophages, FRET pair-labeled polymeric micelles were formulated from acid-responsive and non-responsive block copolymers. To ensure sufficient intense dye labeling also after 48 h incubation, the two dye-labeled block copolymers were mixed with the non-labeled block copolymers in a weight ratio of 1:1:1. After the addition of sterile PBS, the final particle concentration was 1.0 mg/mL. Both samples were filtered through hydrophilized PTFE syringe filters (0.20 µm pore size, Macherey-Nagel). Dye loading was evaluated by UV/vis spectroscopy and fluorescence spectroscopy using a TECAN Spark® plate reader (Figure S67A–B).

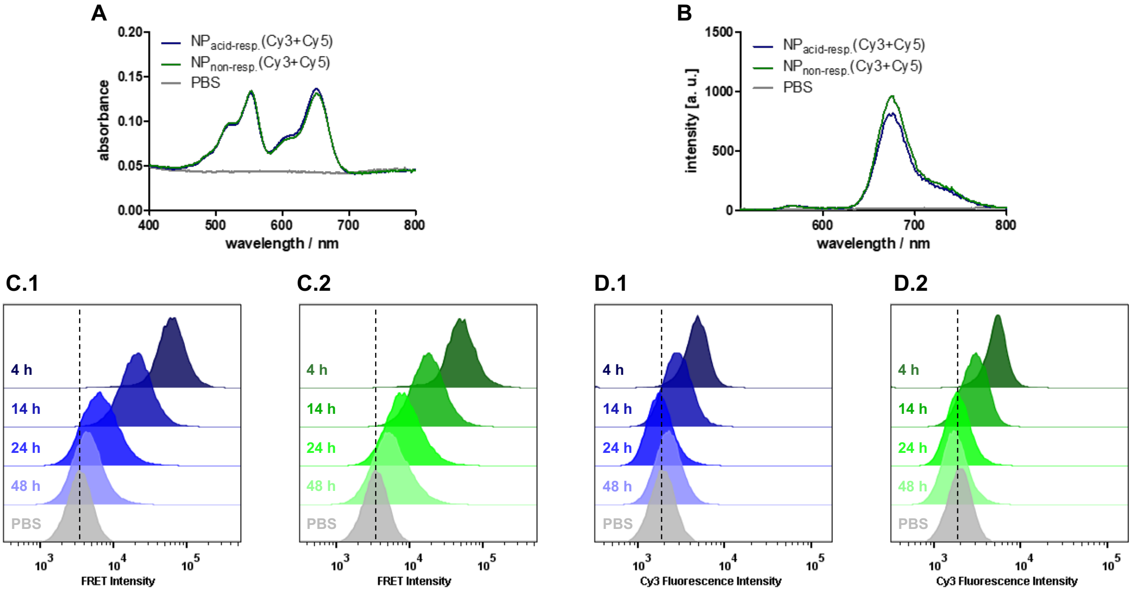

Figure S67: Time Dependent FRET analysis in RAW-Blue™ macrophages. **A**: UV/vis absorption spectra of acid-responsive and non-responsive nanoparticles. **B**: Fluorescence spectra of both samples (λ_{ex} = 488 nm, exbw.: 5 nm, embw: 5 nm). **C**: Fluorescence intensity distributions detected by flow cytometry in the FRET channel of acid-responsive (**C.1**) and non-responsive nanoparticles (**C.2**). **D**: Fluorescence intensity distributions detected in the Cy3 channel of acid-responsive (**D.1**) and non-responsive nanoparticles (**D.2**).

A 24-well plate was filled with 900 µL cell suspension per well ($0.17 \cdot 10^6$ cells/mL, 150,000 cells/well). The 24-well plate was stored in an incubator (37 °C, 5% CO_2,) for 24 h. After the cells adhered to the well bottom 100 µL of a sterile prepared polymeric micelle solution was added resulting in a final polymeric micelle concentration of 100 µg/mL. After pulsing the cells with the FRET pair-labeled nanoparticle samples for 4 h, the supernatant was removed, and the cells were washed with 1 mL PBS per well. Triplicates of cells incubated with each nanoparticle samples or PBS were directly analyzed. Further samples were incubated with 1 mL fresh cell culture medium for further 10, 20 or 44 h. The samples designated for direct analysis were incubated with 500 µL of dissociation buffer (0.5 mM EDTA in PBS, pH = 7.4) for 20 min. The detached cells were transferred into micro centrifuge tubes and stored on ice. The cells were separated from the dissociation buffer by centrifugation (10 min, 1,000 rpm, 5 °C) and resuspended in 200 µL PBS. Flow cytometry analysis was performed using a BD Accuri™ C6 Plus flow cytometer. All other samples were analyzed accordingly at the respective time points. To monitor Cy3 fluorescence the FL2 channel was used (λ_{ex} = 488 nm, detection filter 585/40 nm). The FRET signal was detected using the FL3 channel (λ_{ex} = 488 nm, detection filter 670nm/LP). The measurements were run until 30,000 cells were counted. Each sample was conducted as triplicate (n = 3). Data were processed using FlowJo software package (vX.0.7, FlowJo, LLC, OR, USA). The FRET ratio was calculated from the FRET channel MFI (difference of cells treated with particle samples and PBS) and the Cy3 channel MFI (difference of cells treated with particle samples and PBS).

$$\text{FRET ratio} = (MFI_{FRET}(NP) - MFI_{FRET}(PBS)) / (MFI_{Cy3}(NP) - MFI_{Cy3}(PBS))$$

IN VIVO DISTRIBUTION AND DEGRADATION STUDY

Method Evaluation

To test in vivo probing with the Cy5/IRDye800RS FRET couple, acid-responsive block copolymers labeled with the two fluorescent dyes were co-formulated. In addition, single dye-labeled samples were formulated as well. Dye loading was analyzed by UV/vis spectroscopy and fluorescence spectroscopy (λ_{ex} = 550 nm, exbw = 20 nm, embw = 20 nm) in a transparent 96 well plate using a TECAN Spark® plate reader (Figure S68A). To investigate whether the observed Cy5 quenching can also be detected with a *in vivo* imaging system (IVIS) the samples were imaged in a micro centrifuge tube. The addition of 10vol% HCl (1 M) led to Cy5 fluorescence recovery (Figure S68B).

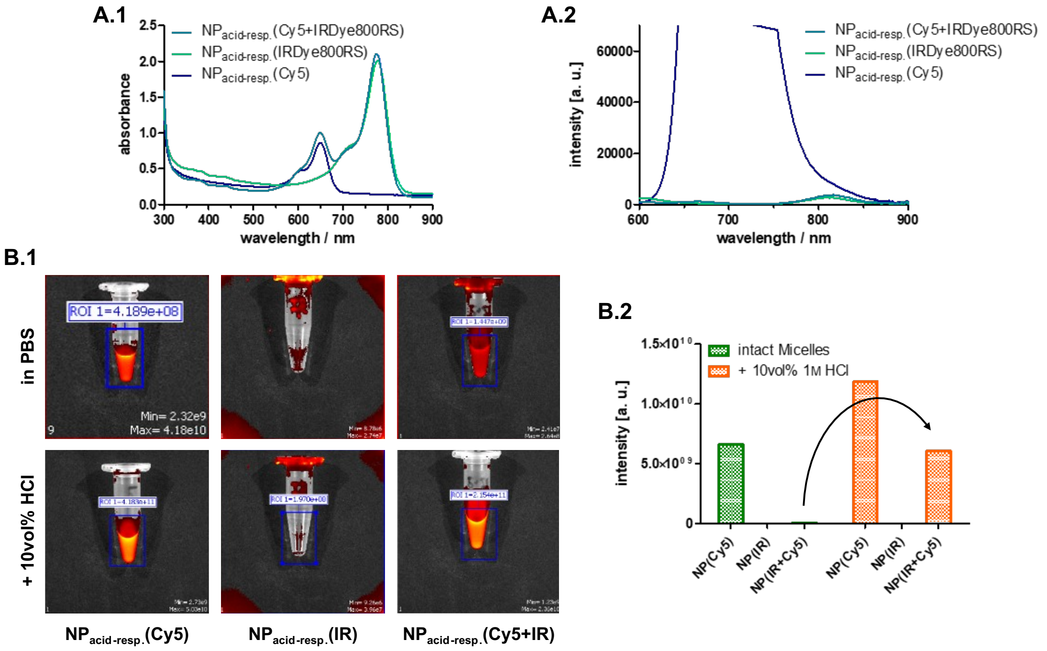

Figure S68: Pre-study demonstrating Cy5 quenching as *in vivo* probe to evaluate micelle unfolding. **A.1**: Absorption spectra of acid-responsive micellar nanoparticles labeled with Cy5, IRDye800RS and the micellar nanoparticle co-formulated with both dyes. **A.2**: Fluorescence spectra of the samples revealing an intense Cy5 fluorescence for the Cy5 only-labeled nanoparticles, no emission for IR dye only-labeled nanoparticles and complete quenching of the Cy5 fluorescence in case of the co-formulated micelles. **B**: IVIS test measurements of these samples before and after the addition of 10vol% HCl (1 M). **B.1**: IVIS images of all samples. **B.2**: Evaluation of the average radiant efficiency (p*s*cm²*sr/µW*cm²) showing the restorage of Cy5 fluorescence for the co-formulated dye sample after acidification (arrow).

In vitro and *in vivo* Sample Preparation

For the *in vivo* distribution and degradation study, micellar nanoparticles were formulated from acid-responsive block copolymers and the non-responsive control system. Three different formulations of the two carriers were tested: 1) Cy5-labeled micelles, 2) Cy5+IRDye800RS-labeled micelles and 3) Cy5+IMDQ-loaded micelles. For all samples the overall carrier concentration was 4.0 mg/mL and the targeted Cy5 loading was 50 µmol/L. For IRDye800RS-labeled samples an equimolar dye labeling was targeted (50 µmol/L) and for IMDQ-labeled samples it was aimed for an adjuvant loading of 100 mg/mL. After mixing the respective end group-labeled block copolymers with non-labeled block copolymer carrier material, the samples were dissolved in sterile PBS and sonicated for 20 min. As references a combination of soluble IMDQ and Cy5 was used as well as PBS. All samples were characterized by UV/vis spectroscopy (Figure S69).

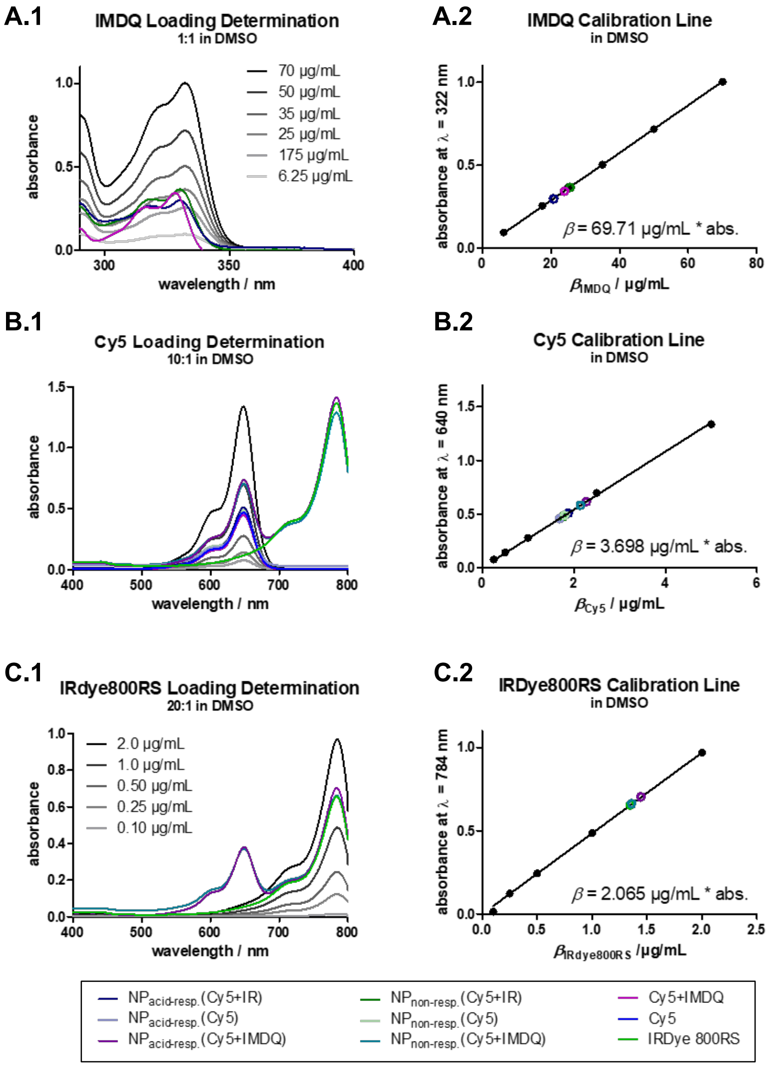

A.3

	Abs.	Loading / µmol/L	Efficiency
$NP_{acid\text{-}resp.}$ (Cy5+IMDQ)	0.297	41.66	42%
$NP_{non\text{-}resp.}$ (Cy5+IMDQ)	0.365	51.20	51%
Cy5+IMDQ	0.342	47.98	48%

B.3

	Abs.	Loading / µg/mL	Loading / µmol/L	Effi-ciency
$NP_{acid\text{-}resp.}$ (Cy5+IR)	0.615	22.74	36.90	74%
$NP_{non\text{-}resp.}$ (Cy5+IR)	0.581	21.49	34.87	70%
$NP_{acid\text{-}resp.}$ (Cy5)	0.459	16.99	27.57	55%
$NP_{non\text{-}resp.}$ (Cy5)	0.487	18.02	29.24	58%
$NP_{acid\text{-}resp.}$ (Cy5+IMDQ)	0.509	18.81	30.52	61%
$NP_{non\text{-}resp.}$ (Cy5+IMDQ)	0.477	17.64	28.62	57%

C.3

	Abs.	Loading / µg/mL	Loading / µmol/L	Effi-ciency
$NP_{acid\text{-}resp.}$ (Cy5+IR)	0.703	28.91	30.49	61%
$NP_{non\text{-}resp.}$ (Cy5+IR)	0.662	27.22	28.71	57%
IRDye800RS	0.655	26.95	28.42	57%

Figure S69: Drug and dye loading determination for the *in vivo* particle distribution, degradation and immunostimulation studies. **A**: IMDQ loading determination. **A.1** shows absorption spectra of IMDQ containing samples next to the calibration spectra. In **A.2** the IMDQ calibration line is shown from which the drug loading was calculated, that is shown in **A.3**. In analogy, Cy5 (**B**) and IRDye800RS (**C**) loading was determined.

Confocal Fluorescence Microscopy (CFM) of RAW Macrophages

RAW-Blue^{TM} macrophages were once more seeded in an eight-well polystyrene microscopy chamber supplied by SARSTEDT ($0.28 \cdot 10^6$ cells/mL, 180 µL, 50,000 cells/well). The eight-well plate was stored in the incubator (37 °C, 5% CO_2,) for 24 h. After the cells adhered to the well bottom, 20 µL of the 1 mg/mL polymeric micelle solutions (either Cy5-labeled micelles, IRDye800RS-labeled micelles or Cy5+IRDye800RS-loaded micelles) were added to each well resulting in a final polymeric micelle concentration of 100 µg/mL. PBS was used as reference. After another 24 h incubation the cell culture medium was removed, and cells were washed with PBS (3 x 200 µl). Subsequently, 200 µl of 4% paraformaldehyde were added and fixed for 15 min (37 °C, 5% CO_2,). Cells were washed again with PBS (3 x 200 µl) and secured in DAPI containing mounting medium (Immunoselect Antifading Mounting Medium DAPI by Dianova). Images were taken using a 63x water immersion objective on a Leica Stellaris8 confocal microscope (Wetzlar, Germany). Leica's software LAS X and ImageJ 2.16.0 software were used for processing.

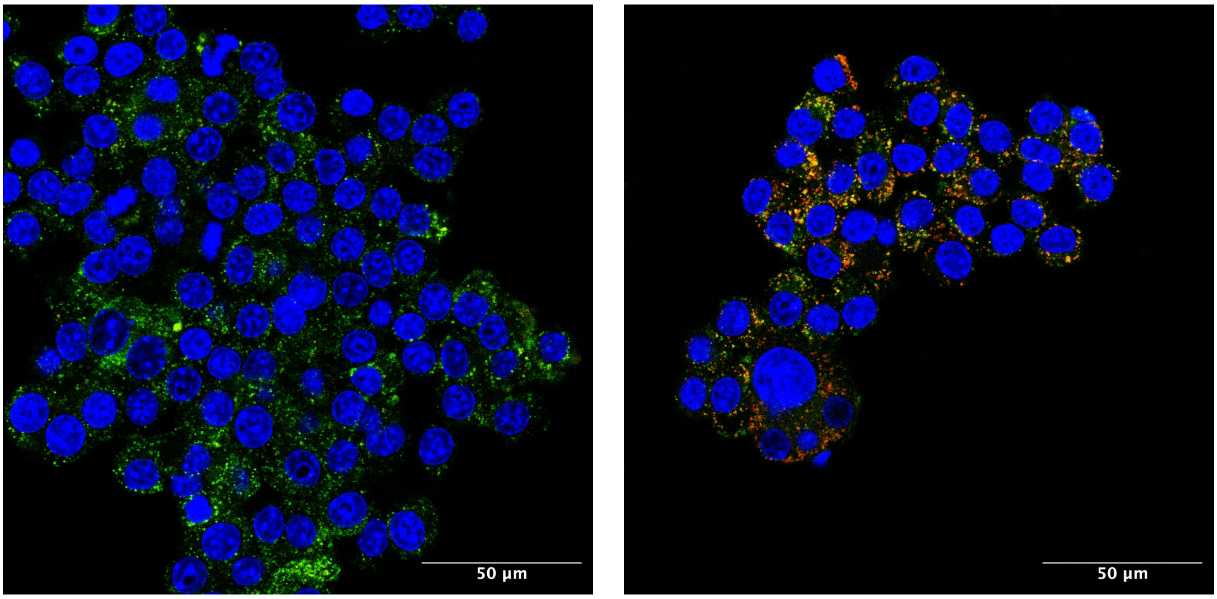

(1) Benzyl Ester Cy5+NIRdye **(2) Benzyl Ketal Cy5+NIRdye**

Figure S70: Confocal fluorescence microscopy images of Cy5 and IRDye800RS (NIRdye) labeled non-responsive benzyl ester block copolymer micelles (1) (left) and acid-responsive benzyl ketal block copolymer micelles (2) (right). Green: IRDye800RS fluorescence. Red: Cy5 fluorescence. Blue: DAPI. While the Cy5 fluorescence for the non-responsive benzyl ester block copolymer micelles (1) is almost absent (left), the Cy5 fluorescence could be recovered for the acid-responsive benzyl ketal block copolymer micelles (2) (right).

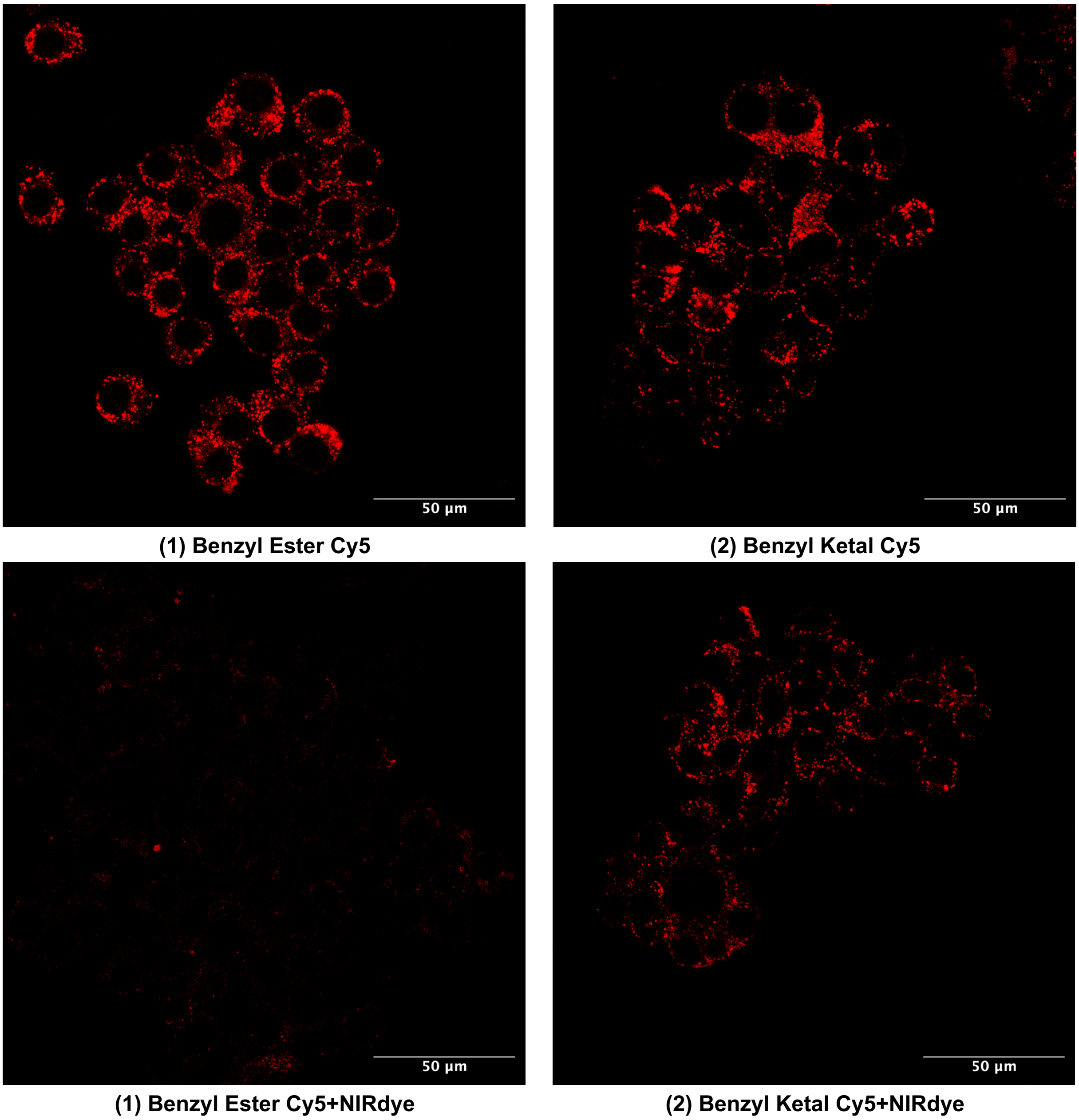

Figure S71: Cy5 fluorescence (in red) recorded by confocal fluorescence microscopy of Cy5 alone or Cy5 and IRDye800RS (NIRdye) labeled non-responsive benzyl ester block copolymer micelles (1) (left) and acid-responsive benzyl ketal block copolymer micelles (2) (right). Green: IRDye800RS fluorescence. While the Cy5 fluorescence for the non-responsive benzyl ester block copolymer micelles (1) is still very much quenched by the IRDye800RS dye (left), the Cy5 fluorescence could be recovered for the acid-responsive benzyl ketal block copolymer micelles (2) (right).

.

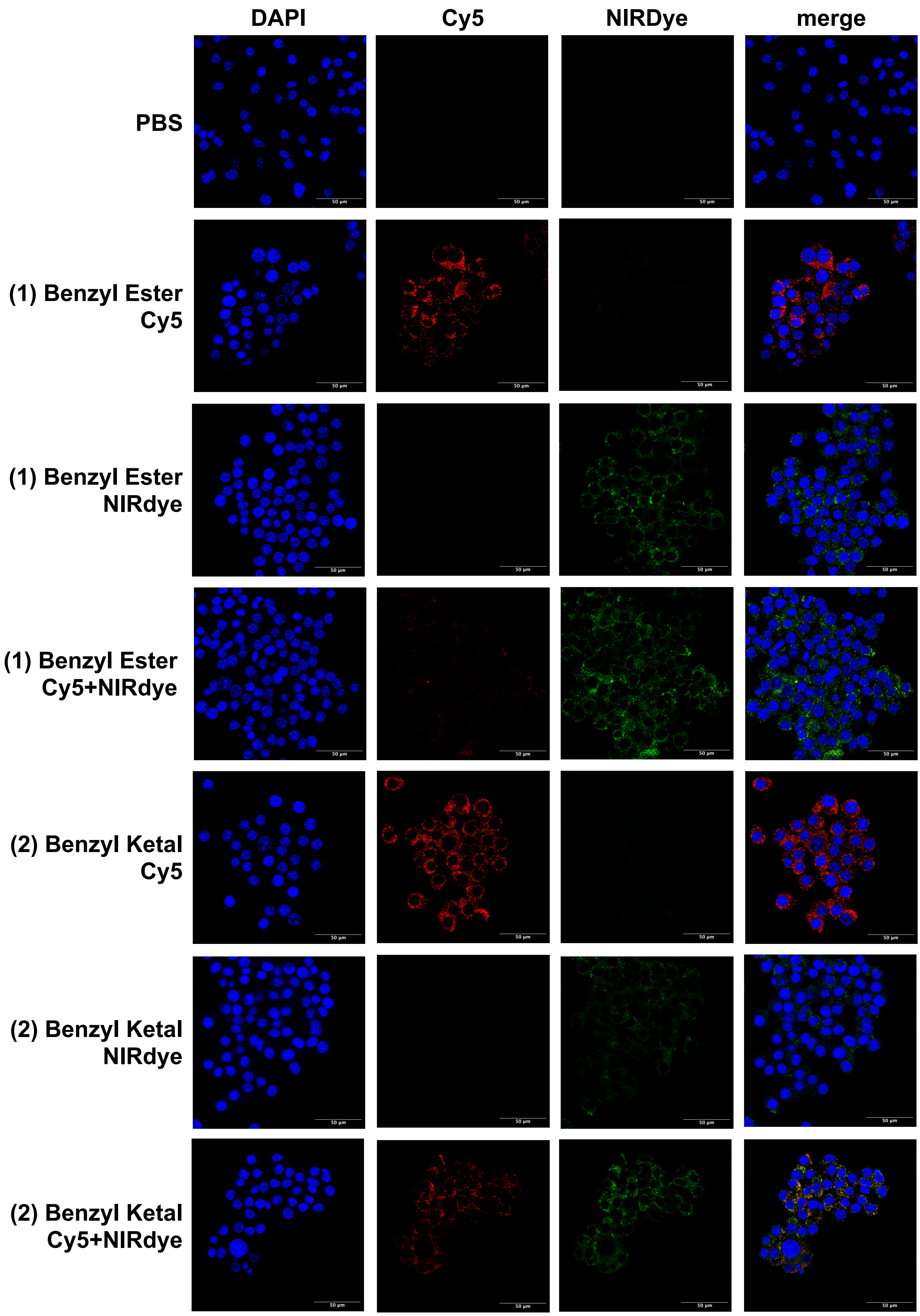

Figure S72: Overview of confocal fluorescence microscopy images for either Cy5 or IRDye800RS (NIRdye) alone as well as double labeled non-responsive benzyl ester block copolymer micelles (1) (left) and acid-responsive benzyl ketal block copolymer micelles (2) (right). Green: IRDye800RS fluorescence. Red: Cy5 fluorescence. Blue: DAPI. While the Cy5 fluorescence for the non-responsive benzyl ester block copolymer micelles (1) is almost absent when the IRDye800RS is co-delivered, the Cy5 fluorescence could be recovered for its corresponding acid-responsive benzyl ketal block copolymer micelles (2), confirming the intracellular unfolding of the block copolymer micelles.

Animals

Animal studies were approved by local authorities (Landesuntersuchungsamt (LUA) Rhineland-Palatinate, Germany) with the reference number G 20-1-123. 5–8-week-old male C57BL/6N-Tyrc-Brd/BrdCrCrl mice were obtained from Charles River Laboratories (Calco, Italy) and kept at the University Medical Center Mainz (Germany) with food and water supply *ad libitum* according to the "Guide for Care and Use of Laboratory Animals" until experimental procedures.

In vivo Biodistribution Study

Nanoparticles (150 μL PBS) were injected intravenously into the tail veins of C57BL/6N-Tyrc-Brd/BrdCrCrl mice (male, 5–8 weeks). After 24 h, blood was taken from the face vein and organs (heart, lung, liver, spleen, kidney, and inguinal lymph nodes) were dissected. Mice were imaged *via* small animal fluorescence imaging (IVIS® SpectrumCT, Perkin Elmer, Waltham, MA) at predetermined timepoints (whole mouse: 3.

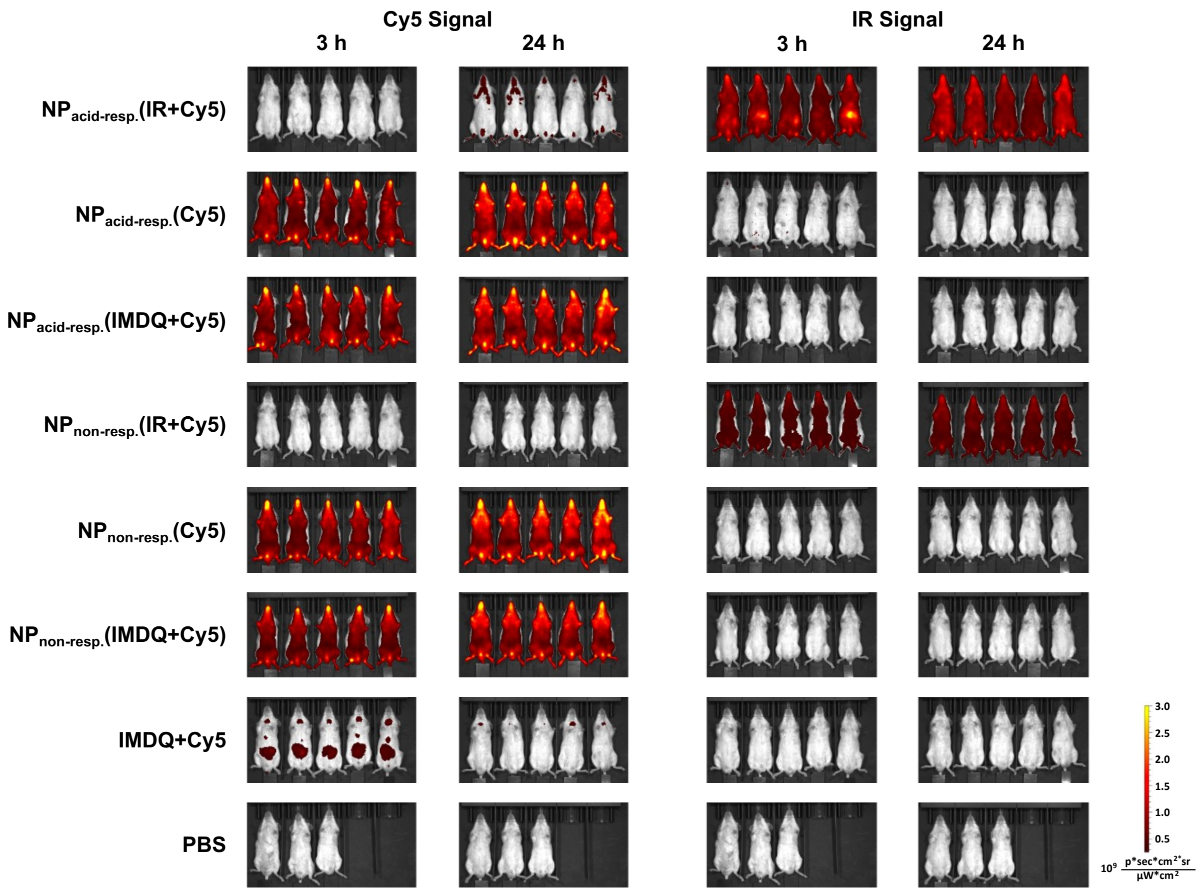

Figure S73: IVIS images of mice treated with all tested samples analyzing the Cy5 signal (λ_{ex} = 640 nm, exbw = 20 nm, λ_{ex} = 680 nm, embw = 20 nm; left) and the IR signal (λ_{ex} = 745 nm, exbw = 20 nm, λ_{ex} = 820 nm, embw = 20 nm; right).

Ex vivo Organ Distribution

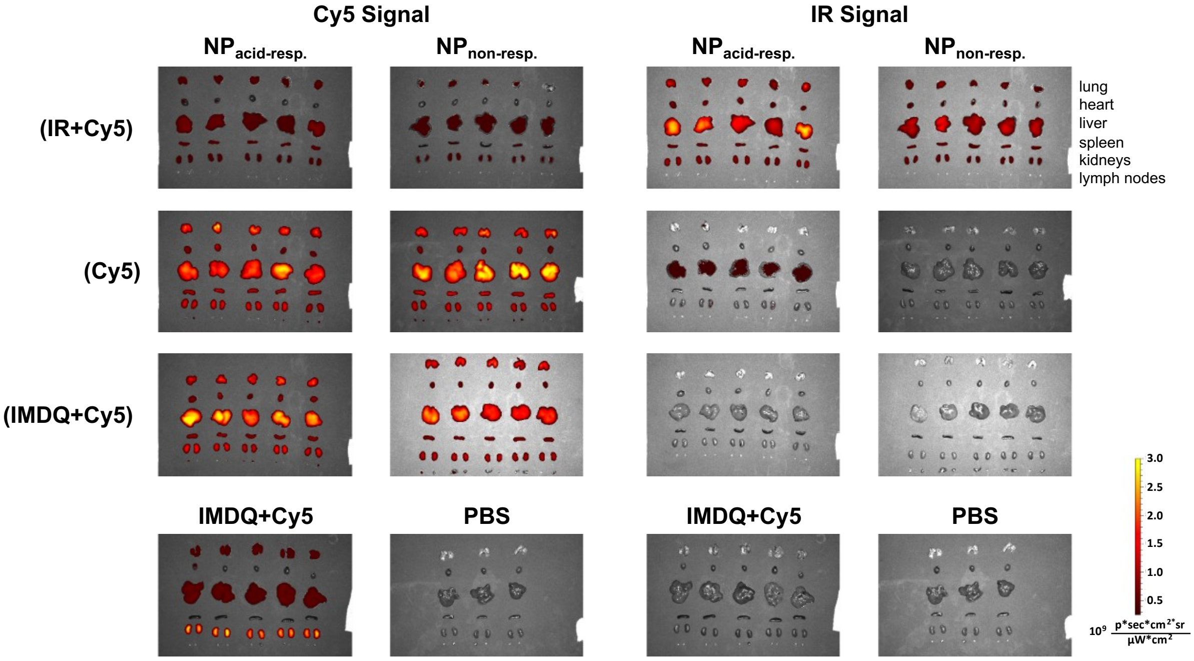

Figure S74: IVIS images of harvested organs analyzing the Cy5 signal (λ_{ex} = 640 nm, exbw = 20 nm, λ_{ex} = 680 nm, embw = 20 nm; left) and the IR signal (λ_{ex} = 745 nm, exbw = 20 nm, λ_{ex} = 820 nm, embw = 20 nm; right).

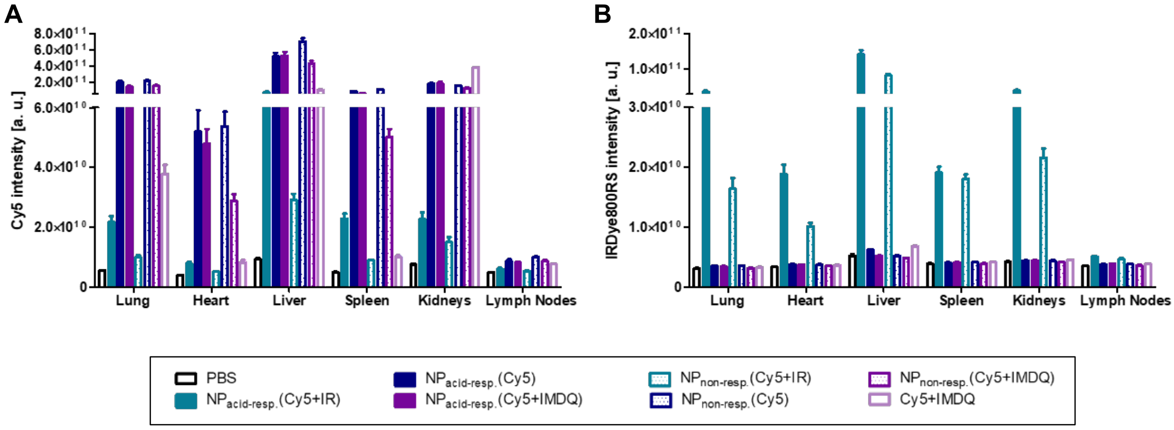

Figure S75: Semiquantitative *ex vivo* analysis of Cy5 (**A**) and IRDye800RS (**B**) organ distribution (n = 5).

Flow Cytometry Analysis of Splenocytes

Isolation of Splenocytes

Spleens of C57BL/6 mice were dissected and mechanically disrupted through a 40 µm cell strainer to obtain a single cell suspension. Erythrocytes were lysed using 2 mL Gey's Red Cell Lysis buffer (H_2O bidest., 100 µM EDTA, 10 mM KHCO3 and 155 mM NH_4Cl) for 1 min at room temperature. Subsequently, cells were washed using an IMDM-based culture medium containing 5% FBS (PAN Biotech, Aidenbach, Germany), 2 mM L-glutamine, 100 IU/mL penicillin, 100 µg/mL streptomycin and 50 µM β-mercaptoethanol. Isolated splenocytes were used for flow cytometric analyses.

Cell Staining

Cells were washed with PBS and Fc receptors were blocked with anti-CD16/CD32 (clone 2.4G2) for 10 min at room temperature to prevent unspecific Fc-mediated binding. Subsequently, spleen cells were incubated with fluorophore-conjugated antibodies for 20 min at 4 °C: anti-CD11c (PE-CF594, clone N418), anti-CD11b (eFluor450, clone M1/70), anti-CD172a/SIRP-a (PE-Cy7, clone P84), anti-I-A/I-E (MHC II) (PerCP-Cy5.5, clone M5/114.15.2), anti-Siglec-H (BV711, clone 440c), anti-CD19 (FITC, clone eBio1B3 (1D3)), anti-CD3ε (FITC, clone 145-2C11), anti-CD14 (FITC, clone rmC5-3), anti-NK1.1 (FITC, clone PK136), anti-Ly6G (FITC, clone 1A8). Dead cells were stained using Fixable Viability Dye (eFluor506, 1:1000 in PBS; Thermo Fisher Scientific) for 20 min at 4 °C. Subsequently, the samples were washed and fixed (4% paraformaldehyde, 2 mM EDTA).

Flow Cytometry

Flow cytometric experiments were performed on an *Attune^{TM} NxT* flow cytometer equipped with *Attune^{TM} NxT* Software v3.1.1 (both from Thermo Fisher). Signals were recorded using the VL1 channel for eFluor450 (λ_{ex} = 405 nm, band pass filter 440/50 nm), VL4 channel for BV711 (λ_{ex} = 405 nm, band pass filter 710/50 nm), BL1 channel for FITC (λ_{ex} = 488 nm, band pass filter 530/30 nm), BL3 channel for PerCP-5.5 (λ_{ex} = 488 nm, band pass filter 695/40 nm), YL2 channel for PE-CF594 (λ_{ex} = 561 nm, band pass filter 620/15 nm), YL4 channel for PE-Cy7 (λ_{ex} = 561 nm, band pass filter 780/60 nm), and the RL1 channel for Cy5 (λ_{ex} = 638 nm, emission filter 670/14 nm), respectively. Data were evaluated following the gating strategies shown below (Figure S76). The corresponding median fluorescent intensity (MFI) were considered for evaluation. Moreover, relative frequencies of counted cell populations were identified by referencing them to mice treated with PBS.

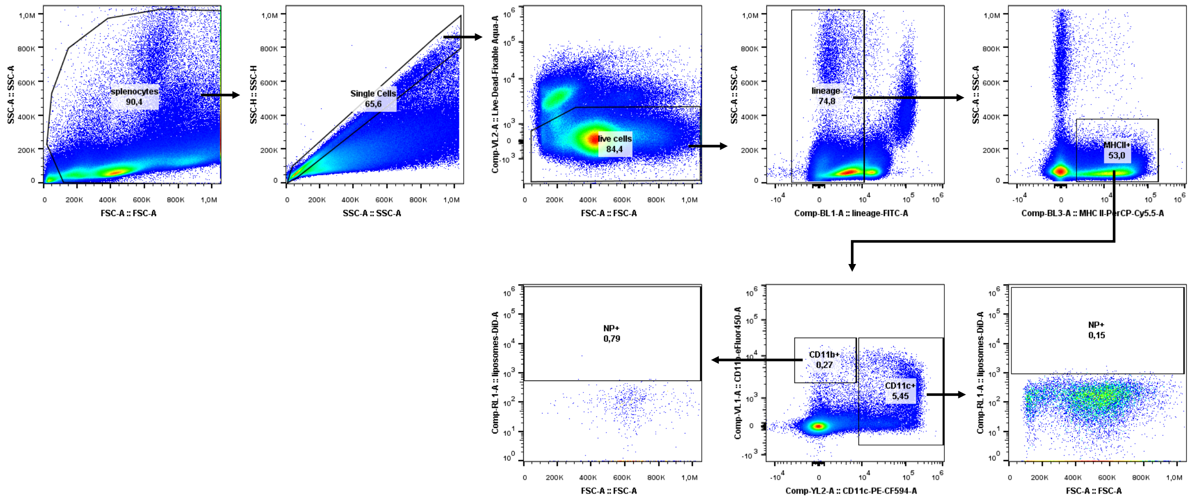

Figure S76: Gating strategy for single cell fluorescence cytometry of splenocytes. First, debris was excluded by gating on FSC and SSC before identification of single cells. FVD was used to exclude dead cells and specific markers or combinations of markers were used to gate on subpopulations of dendritic cells and macrophages. Lineage: anti-CD19, anti-CD3ε, anti-CD14, anti-NK1.1 and anti-Ly6G.

Flow Cytometry of Blood Sera

Cytokine concentrations from the collected blood samples were measured using a multiplex bead-based immunoassay (LEGEND-plex Mouse Anti-Virus Repsone Panel (13-Plex), BioLegend, San Diego, CA, USA) according to the manufacturer's instructions. Samples were analyzed by flow cytometry, and data were evaluated using LEGENDplex^{TM} Qognit (v8.0, BioLegend, San Diego, CA, USA).

Table S1: Mean cytokine and chemokine levels measured from serum samples of mice from all groups (n=5; PBS group n=3) with their associated standard deviation. Concentrations lower than the detection range are written in *italics*. Shown molecules are INF-γ, CXCL1, TNF-α and CCL2.

	INF-γ	CXCL1	TNF-α	CCL2
Data / Group	MW ± SD [pg/mL]	MW ± SD [pg/mL]	MW ± SD [pg/mL]	MW ± SD [pg/mL]
Ketal (IR+Cy5)	*5,43 ± 2,10*	51,67 ± 15,62	*0,00 ± 0,00*	*15,69 ± 3,29*
Ketal (Cy5)	*5,06 ± 0,63*	42,00 ± 4,98	*0,00 ± 0,00*	*12,84 ± 2,78*
Ketal (IMDQ+Cy5)	*7,90 ± 1,78*	130,09 ± 69,10	*2,55 ± 5,11*	*41,19 ± 16,38*
Benzyl (IR+Cy5)	*9,67 ± 4,91*	20,35 ± 7,48	*6,08 ± 12,17*	*18,62 ± 13,61*
Benzyl (Cy5)	*6,68 ± 3,01*	20,95 ± 4,15	*0,00 ± 0,00*	*13,95 ± 2,87*
Benzyl (IMDQ+Cy5)	*5,87 ± 2,21*	21,61 ± 5,00	*0,00 ± 0,00*	*13,85 ± 4,15*
IMDQ+Cy5 (free)	*30,21 ± 3,63*	693,10 ± 370,42	*76,28 ± 6,00*	*219,09 ± 61,66*
PBS	*12,60 ± 6,26*	69,45 ± 13,54	*4,26 ± 6,02*	*16,52 ± 4,30*

Table S2: Mean cytokine and chemokine levels measured from serum samples of mice from all groups (n=5; PBS group n=3) with their associated standard deviation. Concentrations lower than the detection range are written in *italics*. Shown molecules are IL-12, CCL5, IL-1 and CXCL10.

	IL-12	CCL5	IL-1	CXCL10
Data / Group	MW ± SD [pg/mL]	MW ± SD [pg/mL]	MW ± SD [pg/mL]	MW ± SD [pg/mL]
Ketal (IR+Cy5)	*1,67 ± 1,43*	*7,87 ± 2,85*	*2,85 ± 1,65*	49,13 ± 8,45
Ketal (Cy5)	*4,15 ± 3,15*	*6,79 ± 1,61*	*3,47 ± 1,80*	37,87 ± 6,67
Ketal (IMDQ+Cy5)	*3,15 ± 1,44*	*8,09 ± 0,80*	*10,81 ± 15,09*	818,91 ± 363,40
Benzyl (IR+Cy5)	*2,69 ± 0,91*	*4,58 ± 1,18*	53,38 ± 99,40	34,86 ± 23,48
Benzyl (Cy5)	*2,68 ± 1,36*	*5,40 ± 1,77*	*4,77 ± 4,11*	26,92 ± 8,26
Benzyl (IMDQ+Cy5)	*2,54 ± 0,89*	*4,49 ± 2,10*	*2,80 ± 1,38*	25,11 ± 16,61
IMDQ+Cy5 (free)	*4,95 ± 0,50*	89,71 ± 18,00	*16,36 ± 5,71*	2827,16 ± 539,91
PBS	*2,64 ± 1,47*	*16,89 ± 5,55*	*11,46 ± 5,99*	162,19 ± 77,29

Table S3: Mean cytokine and chemokine levels measured from serum samples of mice from all groups (n=5; PBS group n=3) with their associated standard deviation. Concentrations lower than the detection range are written in *italics*. Shown molecules are GM-CSF, IL-10, INF-β, INF-α and IL-6.

	GM-CSF	IL-10	INF-β	INF-α	IL-6
Data / Group	MW ± SD [pg/mL]	MW ± SD [pg/mL]	MW ± SD [pg/mL]	MW ± SD [pg/mL]	MW ± SD [pg/mL]
Ketal (IR+Cy5)	*4,13 ± 1,29*	*7,71 ± 1,23*	*0,68 ± 0,35*	*4,95 ± 2,44*	*1,90 ± 1,44*
Ketal (Cy5)	*5,46 ± 0,41*	*8,23 ± 1,60*	*2,47 ± 0,61*	*6,46 ± 2,21*	*2,16 ± 0,58*
Ketal (IMDQ+Cy5)	*5,45 ± 1,01*	*10,21 ± 4,89*	*1,90 ± 0,53*	*4,64 ± 1,74*	*4,08 ± 3,06*
Benzyl (IR+Cy5)	*4,10 ± 1,23*	*6,04 ± 3,62*	*1,55 ± 0,53*	*4,89 ± 2,15*	*3,07 ± 2,02*
Benzyl (Cy5)	*4,34 ± 0,84*	*6,74 ± 2,55*	*1,51 ± 0,80*	*4,49 ± 0,84*	*1,58 ± 0,59*
Benzyl (IMDQ+Cy5)	*4,38 ± 1,46*	*6,24 ± 2,84*	*1,84 ± 0,50*	*4,65 ± 2,04*	*1,86 ± 0,89*
IMDQ+Cy5 (free)	*7,50 ± 1,62*	*25,40 ± 8,46*	*4,02 ± 0,72*	46,72 ± 10,68	38,37 ± 9,06
PBS	*6,43 ± 1,68*	*23,49 ± 2,41*	*2,87 ± 1,79*	*7,06 ± 3,20*	*7,01 ± 2,31*

STATISTICS

Data is shown as means ± SD. Comparison was done using one way ANOVA method followed by Bonferroni selected pairs of columns comparisons tests for post-hoc analysis. For all tests a $\alpha = 0.05$ level was defined as statistically relevant deviations from the respective null hypotheses. Data were analyzed using GraphPad Prism Version.5.02 (GraphPad Software, California, US).

LITERATURE

[1] A. V. Hauck, P. Komforth, J. Erlenbusch, J. Stickdorn, K. Radacki, H. Braunschweig, P. Besenius, S. Van Herck, L. Nuhn, *Biomater. Sci.* **2025**, *13*, 1414–1425.

[2] C. Czysch, C. Medina-Montano, N.-J. K. Dal, T. Dinh, Y. Fröder, P. Winterwerber, K. Maxeiner, H.-J. Räder, D. Schuppan, H. Schild, M. Bros, B. Biersack, F. Fenaroli, S. Grabbe, L. Nuhn, *Macromol. Rapid Commun.* **2022**, *43*, 2200095.

[3] L. Bixenmann, J. Stickdorn, L. Nuhn, *Polym. Chem.* **2020**, *11*, 2441–2456.

[4] F. G. K. Baucke, *J. Phys. Chem. B.* **1998**, *102*, 4835–4841.
